# Does TENS Reduce the Intensity of Acute and Chronic Pain? A Comprehensive Appraisal of the Characteristics and Outcomes of 169 Reviews and 49 Meta-Analyses

**DOI:** 10.3390/medicina57101060

**Published:** 2021-10-04

**Authors:** Carole A. Paley, Priscilla G. Wittkopf, Gareth Jones, Mark I. Johnson

**Affiliations:** 1 Centre for Pain Research, Leeds Beckett University, Leeds LS1 3HE, UK; carole.paley@nhs.net (C.A.P.); p.wittkopf@leedsbeckett.ac.uk (P.G.W.); g.j.Jones@leedsbeckett.ac.uk (G.J.); 2 Research and Development Department, Airedale National Health Service (NHS) Foundation Trust, Skipton Road, Steeton, Keighley BD20 6TD, UK

**Keywords:** transcutaneous electric nerve stimulation (TENS), pain, pain management, analgesia, efficacy, neuromodulation, systematic review, meta-analysis

## Abstract

*Background and Objectives:* Uncertainty about the clinical efficacy of transcutaneous electric nerve stimulation (TENS) to alleviate pain spans half a century. There has been no attempt to synthesise the entire body of systematic review evidence. The aim of this comprehensive review was to critically appraise the characteristics and outcomes of systematic reviews evaluating the clinical efficacy of TENS for any type of acute and chronic pain in adults. *Materials and Methods:* We searched electronic databases for full reports of systematic reviews of studies, overviews of systematic reviews, and hybrid reviews that evaluated the efficacy of TENS for any type of clinical pain in adults. We screened reports against eligibility criteria and extracted data related to the characteristics and outcomes of the review, including effect size estimates. We conducted a descriptive analysis of extracted data. *Results*: We included 169 reviews consisting of eight overviews, seven hybrid reviews and 154 systematic reviews with 49 meta-analyses. A tally of authors’ conclusions found a tendency toward benefits from TENS in 69/169 reviews, no benefits in 13/169 reviews, and inconclusive evidence in 87/169 reviews. Only three meta-analyses pooled sufficient data to have confidence in the effect size estimate (i.e., pooled analysis of >500 events). Lower pain intensity was found during TENS compared with control for chronic musculoskeletal pain and labour pain, and lower analgesic consumption was found post-surgery during TENS. The appraisal revealed repeated shortcomings in RCTs that have hindered confident judgements about efficacy, resulting in stagnation of evidence. *Conclusions*: Our appraisal reveals examples of meta-analyses with ‘sufficient data’ demonstrating benefit. There were no examples of meta-analyses with ‘sufficient data’ demonstrating no benefit. Therefore, we recommend that TENS should be considered as a treatment option. The considerable quantity of reviews with ‘insufficient data’ and meaningless findings have clouded the issue of efficacy. We offer solutions to these issues going forward.

## 1. Introduction

Transcutaneous electrical nerve stimulation (TENS) is a non-pharmacological technique used across the world for the management of acute and chronic pain irrespective of cause, including pain related to cancer and its treatment [1]. There has been a longstanding debate about the clinical efficacy of TENS since its introduction into mainstream healthcare in the early 1970s [2]. The first systematic reviews on TENS were published in mid 1990s and raised uncertainty about the clinical efficacy of TENS for acute and for chronic pain [3,4,5]. This uncertainty remains unresolved to this day.

In 2020, an overview of Cochrane reviews on TENS for chronic pain included a descriptive analysis of 51 RCTs (2895 participants) from eight reviews and was unable to conclude with confidence whether TENS was beneficial or harmful when used to manage pain [6]. It was observed that the quality of the eight Cochrane reviews scored high on a checklist to assess multiple systematic reviews (A MeaSurement Tool to Assess systematic Reviews or AMSTAR), whereas the quality of individual RCTs was low due to inadequate sample sizes and risks of bias [7]. Generally, the authors of Cochrane reviews on TENS are reluctant to pool data for meta-analysis due to concern about heterogeneity undermining the precision of the estimates of effect.

Moore et al., suggested that meta-analyses with pooled pain intensity data fewer than 500 participants (or events) in each trial arm are at a high risk of bias and likely to produce effect estimates that are imprecise [8,9]. Authors of non-Cochrane systematic reviews have undertaken meta-analyses, although pooled data is often below 500 events per trial arm. We are aware of two meta-analyses that have come close to the threshold of acceptability suggested by Moore et al., and both found superiority for TENS over placebo; for alleviating chronic musculoskeletal pain [10], and for reducing acute post-operative analgesic consumption [11].

Recently, a review published in *The Lancet*, evaluated the benefits and harms of neuromodulation for chronic pain and reported low-quality evidence of short-term benefit from TENS for neuropathic pain and conflicting evidence for non-neuropathic conditions [12]. The reviewers claimed that they *“…searched MEDLINE, Embase, and Cochrane databases for a 16-year period beginning in December 2004, to December 2020, to identify randomised clinical trials, observational studies, systematic reviews, meta-analyses, and narrative reviews in English for inclusion in the text and tables*” ([12], p. 2111). However, they based their conclusion on only three reviews; one Cochrane review on TENS pain in adults [13]; one overview of Cochrane Reviews on TENS for chronic pain [6]; and a literature review and meta-analysis of TENS for chronic back pain [14]. Despite being recent and conducted using rigorous methodologies, these reviews represent a small selection of available reviews. A free text search of PubMed (25 June 2021) reveals over 100 potential systematic reviews including 500 clinical trials. Evidence syntheses that do not fully or accurately evaluate and/or report all available literature can generate findings that are not reproducible and misrepresent the status of knowledge.

### Aim

The aim of this comprehensive review is to critically appraise the characteristics and outcomes of systematic reviews evaluating the clinical efficacy of TENS for any type of acute and chronic pain in adults.

## 2. Methods

We have published a protocol for a systematic review and meta-analysis of RCTs to evaluate TENS versus placebo TENS on pain intensity. The protocol included a literature search to identify systematic reviews and meta-analysis of TENS in order to conduct a descriptive analysis of the characteristics and outcomes of previously published systematic reviews and meta-analyses as described herein (the Meta-TENS study, Prospero registration number CRD42019125054 [15]).

### 2.1. Literature Search Methods

The purpose of the search was to provide comprehensive coverage of a wide variety of pain conditions (broadly based on the World Health Organisation’s (WHO) International Classification of Disease (ICD-11) categories for pain), at various stages (e.g., acute, chronic) and from various settings (e.g., palliative, community, primary, secondary, tertiary). We searched the following electronic databases using a combination of controlled vocabulary (i.e., medical subject headings (MeSH) and free-text terms) to identify published systematic reviews from inception to the date of the search: Cochrane Library (CENTRAL); MEDLINE (via PubMed); Embase (via OVID); CINAHL (via EBSCO); PsycINFO (via EBSCO); LILACS (via Birme); PEDRO; Web of Science; AMED (via OVID); SPORTDiscus (via EBSCO).

We tailored searches to the individual databases by adapting the MEDLINE search strategy for the other databases listed. Search terms included combinations of the terms Transcutaneous Electric* Nerve Stimulation, Pain, Systematic review, and Meta-analysis (Supplementary Materials Section S1. Search Terms: Systematic Reviews). There were no language restrictions and we identified all relevant systematic reviews irrespective of language and translated articles where possible. The original search was conducted during July 2019 and updated on 8 June 2021.

### 2.2. Eligibility Criteria

We pre-specified TENS as non-invasive electrical stimulation of the skin using surface electrodes with the intention of stimulating peripheral nerves to alleviate pain using a standard TENS device. A standard TENS device “…generates pulsed electrical current delivered in a repetitive manner, with a maximum peak-to-peak amplitude of approximately 60 milliamperes (mA) into a 1 kilohm load” ([1], p. 12) and regardless of the device manufacturer. We considered an intervention TENS if pulsed electrical current was delivered in a repetitive manner using monophasic or biphasic waveforms and pulse frequencies no greater than 250 pulses per second (pps), pulse durations no greater than 1 millisecond (1000 microseconds) and any type of pulse pattern.

We included journal publications of full reports of reviews that included a systematic search for research and evaluation of efficacy using tallies of study outcome or pooling data for meta-analysis for any type of clinical pain in adults. We included systematic reviews of studies, overviews of systematic reviews of studies, and hybrid reviews that included studies and systematic reviews. We included reviews that evaluated TENS treatment on its own or as part of a broader review.

We defined TENS as treatments described by authors as ‘TENS’ and administered using a standard TENS device with the primary intention of stimulating peripheral nerves to alleviate pain; irrespective of technique (e.g., conventional TENS, acupuncture-Like TENS, high-frequency-low-intensity, low-frequency-high intensity, etc.), electrical characteristics of currents (pulsed current width, amplitude, frequency or pattern), dosage and regimen.

We excluded reports:

- that did not undertake an evaluation of TENS using systematic methodology in the broadest sense (e.g., comprehensive reviews, opinion pieces, commentaries);
- That evaluated invasive nerve stimulation techniques (e.g., percutaneous electrical nerve stimulation and electro-acupuncture);
- where TENS was not a primary treatment (i.e., where TENS was a possible comparator);
- where the primary intention of TENS was not to stimulate peripheral nerves to alleviate pain (e.g., TENS for bladder dysfunction, constipation, dementia);
- focused on ‘TENS-like’ currents that considered output specifications of a standard TENS device (e.g., interferential current, microcurrent).

### 2.3. Selection of Reviews

Two review authors (PGW and MIJ) screened abstracts and titles against our eligibility criteria (Supplementary Materials Section S2; Operational Aide Memoires). Duplicates and records that clearly did not satisfy inclusion criteria were removed and full text reports obtained and screened for inclusion in our review. We did not anonymise records of systematic reviews during screening. Disagreements at any stage of the process were resolved by consensus using a third review author as arbiter (CAP or GJ). Violation of any of the following criteria resulted in exclusion from our review:

- report was not a review;
- report did not evaluate ‘standard TENS’;
- report did not evaluate TENS as a primary comparator i.e., TENS was a comparator rather than the primary treatment;
- report did not systematically search for RCTs;
- report did not evaluate pain intensity;
- report did not evaluate clinical pain, i.e., evaluation of healthy human participants.

To overcome double counting of characteristics, we only included the most recent update of a review. We included the most recent Cochrane review unless there had been a subsequent journal publication of a Cochrane review that included additional RCTs. Generally, Cochrane reviews provided greater detail than journal reports. We only included the most recent update of non-Cochrane reviews conducted by the same review team.

### 2.4. Extraction and Management of Information

Two review authors (PGW and MIJ) extracted and tabulated information about:

- clinical condition;
- type and scope of review;
- number of TENS studies, and/or reviews used in the evaluation;
- whether a meta-analysis was undertaken;
- effect size estimates for pain intensity, if calculated;
- conclusion stated by authors as a direct quote from their manuscript.

### 2.5. Data Analysis

#### Analysis of Characteristics of Reviews

We extracted statements of conclusion from each review and categorised them as claiming evidence; tending to favour TENS (+), tending not to favour TENS (i.e., superior or inferior to control/placebo (−)), tending to be conflicting/inconclusive or insufficient to make a judgement (?).

In addition, we independently judged review outcome using criteria based on research by Dechartres et al. [16,17] that suggests meta-analyses based on small to moderately sized trials produce stronger effect estimates than meta-analyses based on large trials. Moore et al. [9] have argued that in meta-analyses of pain outcomes credible estimates of effect need to be based on large trials or from pooling at least 500 events from many moderately sized trials. RCTs may be considered adequately powered with ≥200 patients per treatment arm, moderately powered with 100–199 patients per treatment arm and underpowered with <100 patients per treatment arm [18,19]. Thus, we independently judged outcome according to the following criteria:

- sufficient evidence in favour of TENS (+)—pooled analysis of ≥500 events or at least one RCT with ≥200 participants in each arm of the trial
- sufficient evidence in favour of control/placebo (−)—pooled analysis of ≥500 events or at least one RCT with ≥200 participants in each arm of the trial
- sufficient evidence that is conflicting/inconclusive (=)—no analysis of pooled data and at least two RCTs with ≥200 participants in each arm of the trial that are conflicting
- insufficient evidence to make a judgement (?)—pooled analysis of <500 events or no RCTs with ≥200 participants in each arm of the trial

Finally, we extracted and compared effect size estimates of meta-analyses of data pooled from two or more RCTs for pain intensity for continuous data (i.e., mean difference or standardised mean difference).

## 3. Results

### 3.1. Search Findings

**Figure 1:**
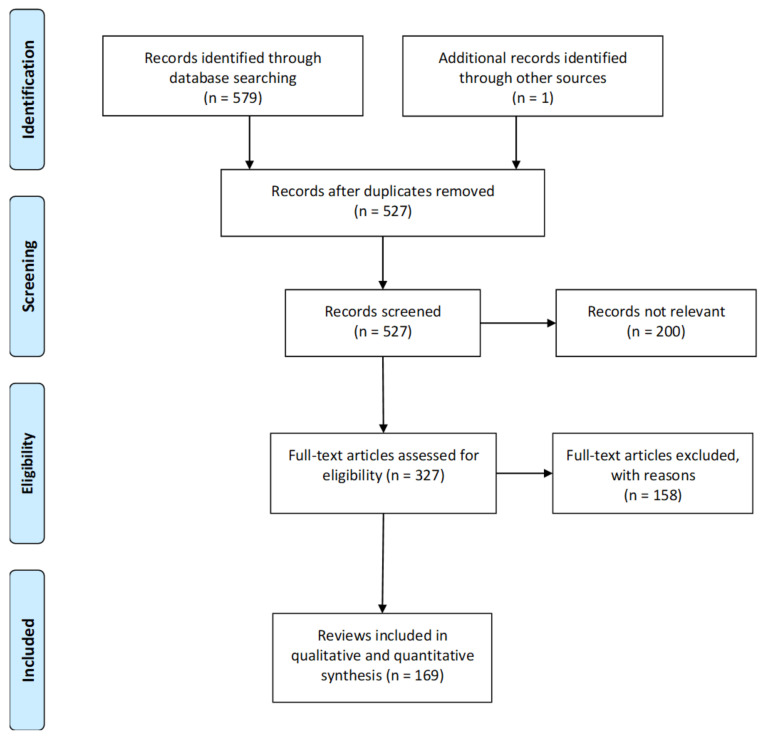
Flow chart of search and screening process.

Our original search yielded 579 records of which, after removal of duplicates, we screened 527 records and reviewed 327 full text reports/Of these 158 were excluded and 169 were included in the review (Figure 1).

### 3.2. Description of Excluded Records

The most common reason for excluding records was that they were narrative reviews on TENS that did not contain a systematic search of literature or that the systematic review had been updated (Supplementary Materials Table S1 Excluded Records with Reasons). Occasionally, we excluded more recent reports or updates of reviews because:

- TENS was in scope in the earlier review but out of scope in a later review. For example, TENS was in-scope in the 2007 report of the European Federation of Neurological Societies (EFNS) guidelines on neurostimulation therapy for neuropathic pain [20], but out of scope in the 2016 report [21].
- the earlier review was more comprehensive and included more studies that the later review. For example, we included a Cochrane review on TENS for labour pain published in 2009 by Dowswell et al. [22] rather than a later journal report of the Cochrane report published in 2011 by the same team (Bedwell et al. [23]).

### 3.3. Characteristics of Included Reviews

**Figure 2:**
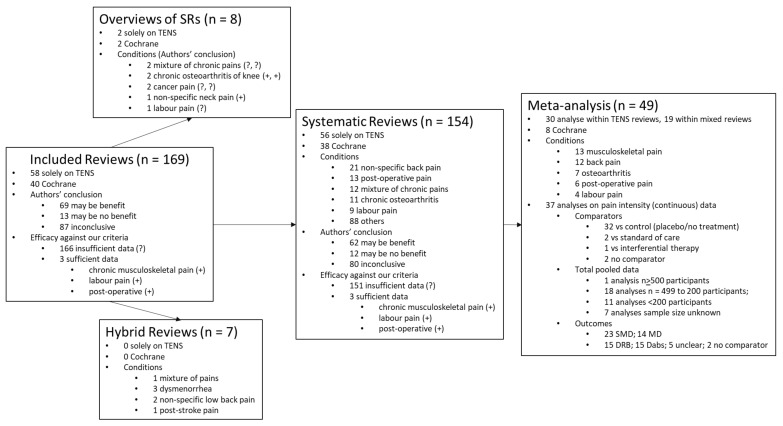
Flow chart summarising the sequence of analyses. Key: SMD, standardised mean difference; MD, mean difference; DRB, difference relative to baseline; Dabs, difference absolute (not relative to baseline); (+) authors’ judgement—evidence tends toward benefit, (−) authors’ judgement—evidence tends toward no benefit (?) authors’ judgement—inconclusive.

Figure 2 presents a flow chart summarising the sequence and outcomes of analyses of the characteristics of the reviews included in our appraisal.

**Figure 3:**
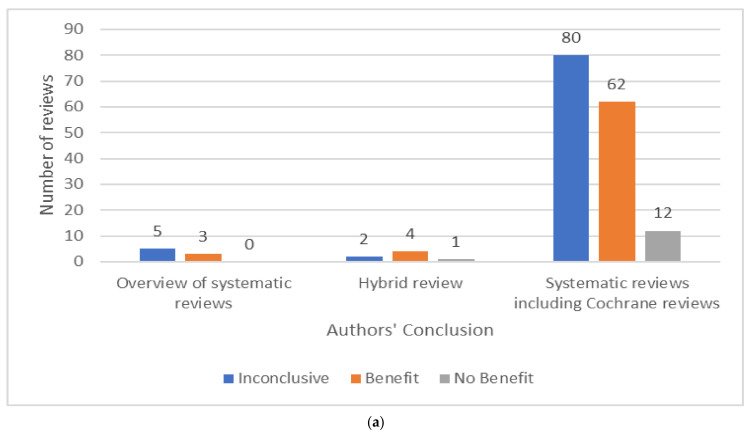

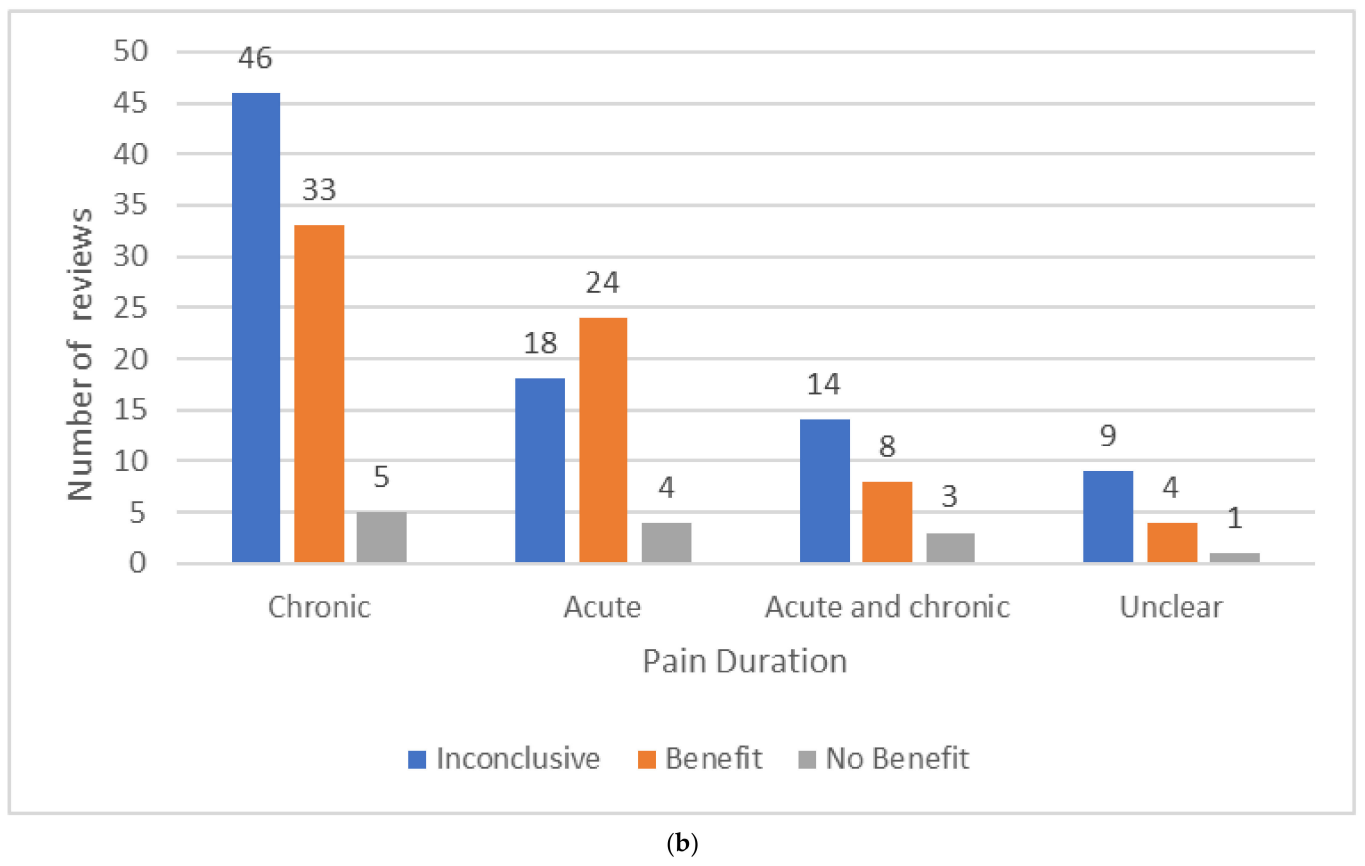

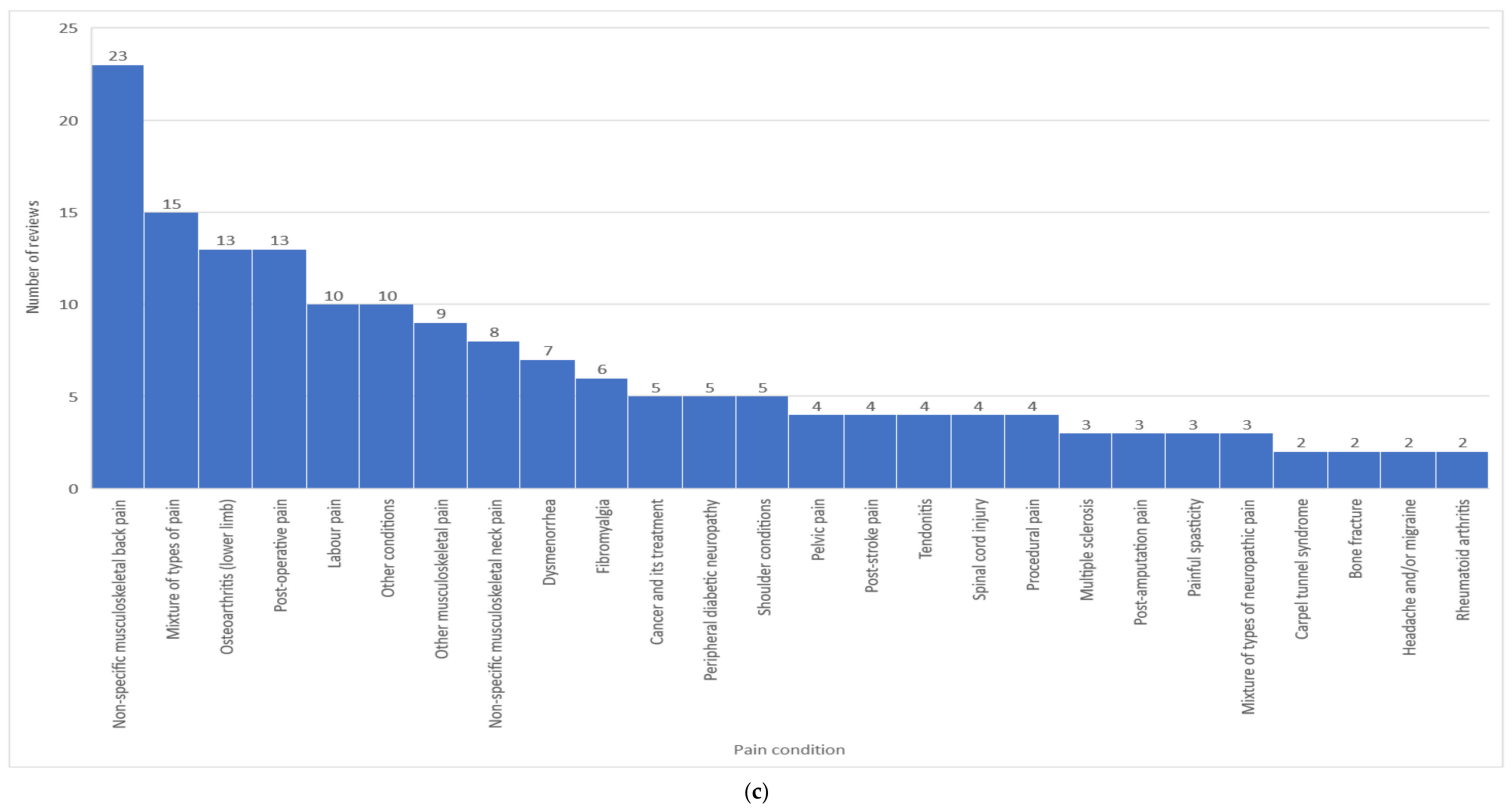
Characteristics of the 169 included reviews. Tally of (**a**) types of review according to our categorization of conclusions of authors, (**b**) duration of pain according to our categorization of conclusions of authors, and (**c**) pain condition.

We categorised 8/169 reports as overviews of systematic reviews that only evaluated previously published systematic reviews; 7/169 reports as hybrid reviews that evaluated clinical studies and previously published systematic reviews; and 154/169 reports as systematic reviews of clinical studies (Figure 3a). There were 37/169 reports produced by Cochrane of which 35 were systematic reviews and two were overviews of Cochrane reviews. There were 84/169 reviews that evaluated TENS for chronic pain, 46/169 for acute pain, 25/169 for both acute and chronic and 14 reports were unclear (Figure 3b).

There were 40/169 reviews that evaluated TENS for ‘non-specific’ musculoskeletal pain excluding arthritic pain or pain associated with tendinopathy (tendinitis). Of the ‘non-specific’ musculoskeletal pain reviews 21 focused on chronic non-specific back pain and eight on chronic non-specific neck pain (Figure 3c). There were 13/169 reviews for osteoarthritis (10 of the knee only, three of knee and hip), 10/169 for labour pain, 13/169 for post-operative pain and 15/169 for a mixture of types of pain. The remainder of the reviews included evaluations of TENS for dysmenorrhea (*n* = 7), fibromyalgia (*n* = 6), cancer (*n* = 5), peripheral diabetic neuropathy (*n* = 5), pelvic pain (*n* = 4), post-stroke pain (*n* = 4), tendonitis (*n* = 4), spinal cord injury (*n* = 4), multiple sclerosis (*n* = 3), shoulder impingement (*n* = 3), post-amputation pain (*n* = 3), neuropathic pain (*n* = 3), procedural pain (*n* = 4), carpel tunnel syndrome (*n* = 2), bone fracture (*n* = 2), headache and/or migraine (*n* = 2) and rheumatoid arthritis (*n* = 2). There were a variety of other conditions with only one review.

#### 3.3.1. Overviews of Systematic Reviews

There were eight reports of overviews of systematic reviews, of which two focussed solely on TENS [6,24], and two were published by Cochrane [6,25]. There were five overviews on chronic pain (two on a mixture of pain conditions, two on osteoarthritis of the knee, and one on cancer pain), one overview on acute pain (labour pain), one overview on acute and chronic cancer pain, and one on non-specific neck pain of uncertain duration. None of the overviews conducted a meta-analysis of pooled data.

#### 3.3.2. Hybrid Reviews

There were seven reports of hybrid reviews of clinical studies and systematic reviews, none of which focused solely on TENS nor were published by Cochrane. There were two hybrid reviews on chronic pain (non-specific low back pain and post stroke pain), three on acute pain (dysmenorrhea), and two on both acute and chronic pain (non-specific back pain and a variety of types of pain). None of the hybrid reviews conducted a meta-analysis of pooled data.

#### 3.3.3. Systematic Reviews

**Figure 4:**
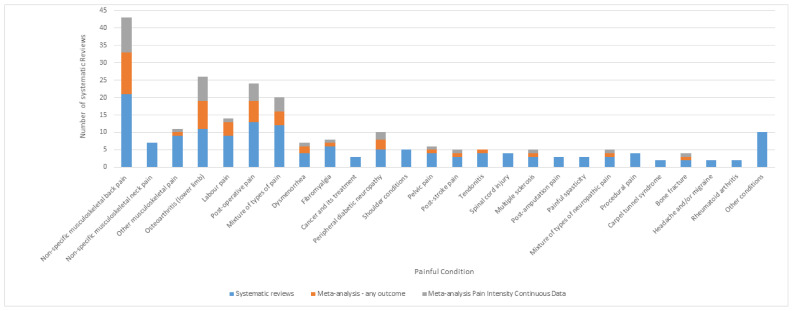
Tally of systematic reviews and meta-analyses for any outcome and for pain intensity (continuous data) according to pain condition (*n* = 154).

There were 154/169 reports of systematic reviews; 56 reviews focused solely on TENS and 98 evaluated multiple treatments including TENS. There were 38 systematic reviews published by Cochrane. There were 77 systematic reviews on chronic pain, 42 on acute pain, 22 on both acute and chronic pain and 13 on pain of uncertain duration. The majority of systematic reviews were conducted on non-specific musculoskeletal pain (*n* = 40), of which non-specific back pain was most common (*n* = 21). A tally of the meta-analyses for all outcomes and for pain intensity continuous data is shown in Figure 4. Generally, less than half of the systematic reviews included a meta-analysis of pooled data and when they did, they meta-analysed pain intensity continuous data.

#### 3.3.4. Meta-Analyses

There were 49 systematic reviews that included a meta-analysis that pooled data from at least two clinical studies; 30 were within systematic reviews that focused solely on TENS and 19 were within reviews of a variety of treatments. There were eight meta-analyses reported as part of a Cochrane review. There were 27 meta-analyses for chronic pain conditions, 16 for acute pain, and six for both acute and chronic. The majority of meta-analyses were for non-specific musculoskeletal pain (*n* = 13) of which 12 were for back pain. There were seven meta-analyses of osteoarthritis, six for post-operative pain, four for labour pain and four for various types of pain (Figure 4).

##### Characteristics of the Analysis of Pain Intensity: Continuous Data

There were 37 analyses that estimated effect size for pain intensity from continuous data (i.e., visual analogue scales or numerical rating scales). The remaining 12 meta-analyses estimated effect sizes for pain intensity as dichotomous outcomes (e.g., relative risk, risk ratio), or analysed other pain outcomes (e.g., analgesic consumption or pain free days), or unclear reporting made it impossible to ascertain the analytical methodology.

Two of the meta-analyses that did not estimate effect size for pain intensity from continuous data met our criteria for sufficient data to judge efficacy for other outcomes. Bjordal et al. [11] pooled data from 1350 participant and found a significant mean reduction in analgesic consumption after TENS to be 26.5% (range 6% to 51%) when compared with placebo. Thuvarakan et al. [26] conducted a responder analysis of pooled data from 11 studies evaluating TENS for labour pain with 700 participants receiving TENS and 626 receiving a control (placebo) intervention. Thuvarakan et al., calculated the risk ratio for participants experiencing moderate (≥30%) or a strong reduction in pain intensity (≥50%) as 1.52 (95% CI, 1.35, 1.70) in favour of TENS.

Of the 37 meta-analyses of pain intensity (continuous data), 10 were for back pain, seven for osteoarthritis, five for post-operative pain, two for peripheral diabetic neuropathy, one for labour pain, and four for a mixture of types of pain (Figure 4).

**Figure 5:**
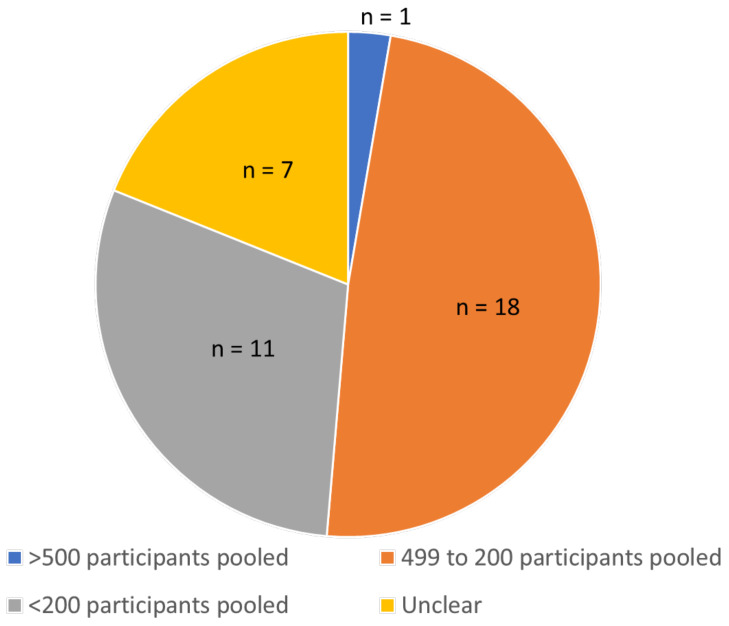
Tally of the size of the total sample of pooled data points in meta-analyses of pain intensity continuous data (*n* = 37).

The mean ± SD number of studies included in the 37 meta-analyses of pain intensity from continuous data was 5.73 + 4.87 (minimum = two studies, maximum = 28 studies, *n* = 37 analyses). There were seven reports that did not state the sample size of pooled data entered into the meta-analysis. The mean ± SD total sample of pooled data was 278 ± 281 participants (minimum = 42, maximum = 1692, *n* = 30 analyses). Only one meta-analysis pooled data from at least 500 participants events; 18 analyses pooled data from 499 to 200 participants; and 11 analyses pooled fewer than 200 participants (Figure 5).

**Figure 6:**
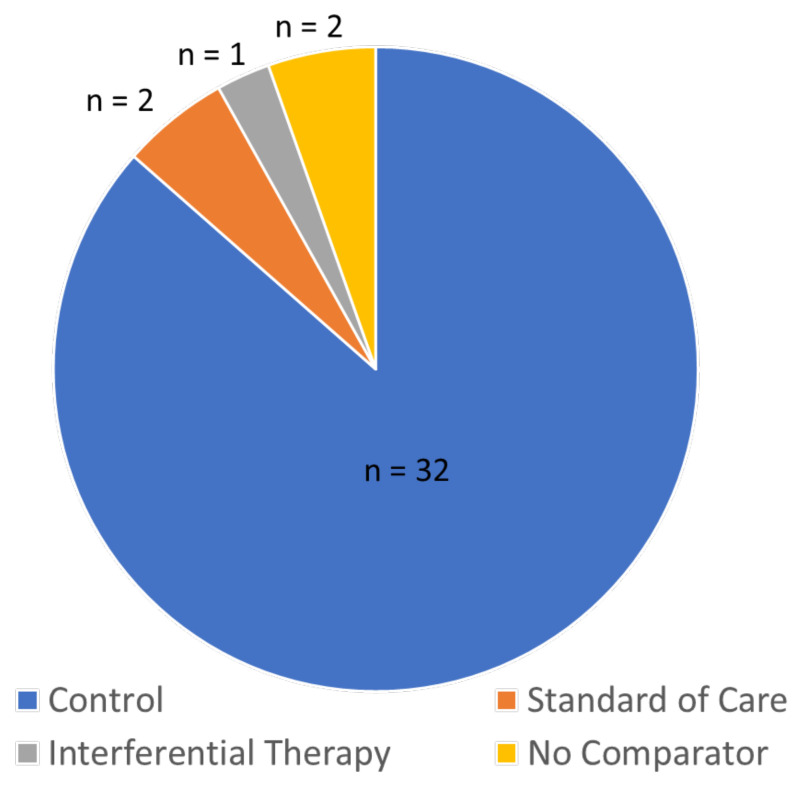
Tally of comparators in meta-analyses of pain intensity continuous data (*n* = 37).

Of the 37 analyses that estimated effect size for pain intensity from continuous data, 32 compared with a ‘control’ group which was generally placebo TENS, two compared TENS with standard of care, one compared TENS with interferential therapy, and two did not include a comparator intervention (i.e., they were effect size estimates for pre-post only) (Figure 6).

**Figure 7:**
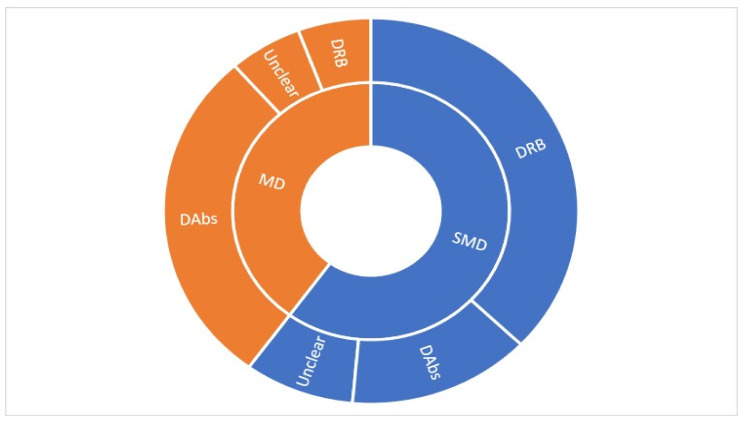
Characteristics of the 35 analyses of pain intensity (continuous data) versus a comparator. Inner ring: Tally of type of effect size estimate (SMD = standardised mean difference, MD = mean difference). Outer ring: Tally of type of outcome (DAbs = absolute difference between groups, DRB = relative difference between groups i.e., difference in change from baseline).

Of the 35 analyses of TENS versus a comparator, 21 calculated SMD and 14 calculated MD (Figure 7) and 15 calculated effect size as absolute difference (i.e., difference in pain intensity at a single time point during or post TENS), 15 as relative difference (i.e., difference in pain intensity during or post TENS as a change from pre-intervention baseline), and five reports were unclear (Figure 7).

There were 18 analyses reporting standardised mean difference (SMD) for TENS versus a control intervention of a placebo/routine care. Of these, 10 calculated SMD as the change in pain intensity during or post TENS compared with pre-intervention baseline (i.e., relative to baseline), five calculated SMD as a difference in pain intensity at a single time point during or post TENS (i.e., absolute difference), and three were unclear about which method was used.

We explored the consistency of inclusion of studies in meta-analyses by identifying studies included in the four reviews on non-specific low back pain published within the previous 10 years (Table 1). There was inconsistency in both the inclusion of studies and in whether study data was extracted for meta-analysis. Only one primary study was included in all reviews Topuz et al. [27]. There were instances of studies being included in some reviews but not others; and instances of pain intensity (continuous) data being extracted from studies in some reviews but not others.

### 3.4. Analysis of Outcomes Irrespective of Condition

#### 3.4.1. All Included Reviews

**Figure 8:**
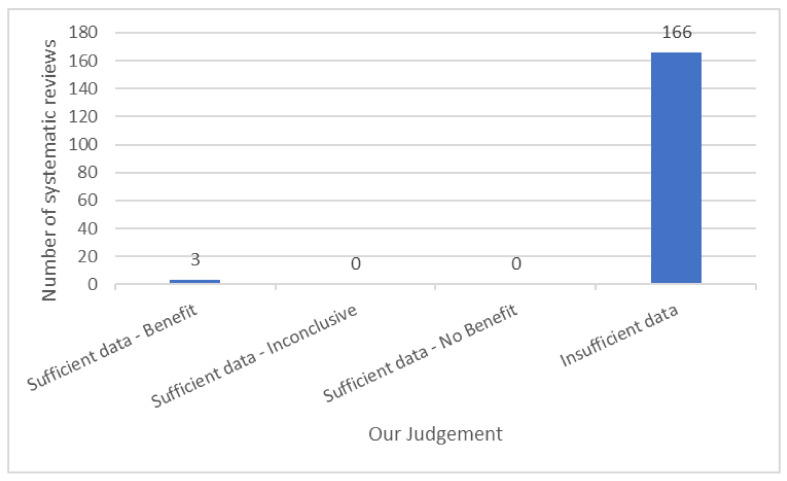
Tally of our judgement of outcome based on our criteria for sufficient data for all reviews (*n* = 169).

The tally of the conclusions of authors of all of the included reviews found that TENS may be efficacious in 69/169 reviews, evidence of no efficacy in 13/169 reviews, and inconclusive evidence in 87/169 reviews (Figure 3a). However, when judged against our criteria, 165/169 reviews had insufficient data to make a judgement (Figure 8). There were no reviews with sufficient data to support evidence of no benefit, no reviews with sufficient data to support evidence that was conflicting (inconclusive) and 3/169 reviews with sufficient data to support evidence of benefit [10,11,26]

#### 3.4.2. Overviews of Systematic Reviews

The tally of the conclusions of authors of the eight overviews found evidence that TENS may be efficacious in three reports; osteoarthritis of the knee [54,55] and non-specific neck pain [56]. The authors of five overviews judged evidence to be inconclusive. When judged against our criteria, there were no overviews with sufficient data to make a judgement.

#### 3.4.3. Hybrid Reviews

The tally of the conclusions of authors of the seven hybrid reviews found evidence that TENS may be efficacious in four reports; paretic upper limb of stroke survivors [57] and dysmenorrhea [58,59,60]. The authors of one hybrid review concluded that evidence suggested no benefit (for non-specific back pain) [61]. When judged against our criteria, there were no hybrid reviews with sufficient data to make a judgement.

#### 3.4.4. Systematic Reviews

**Figure 9:**
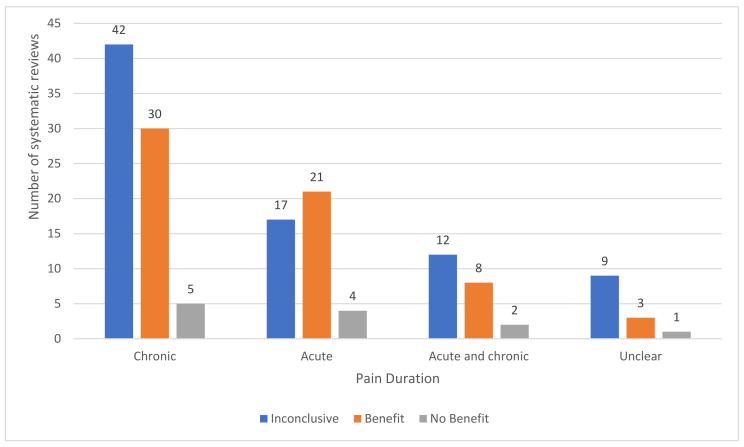
Conclusions of systematic reviews. Tally of our categorisation of conclusions of authors according to pain duration (*n* = 154).

The tally of the conclusions of authors of the 154 systematic reviews found evidence that TENS may be efficacious in 62 reports, evidence of no benefit in 12 reports and inconclusive evidence in 80 reports (Figure 9).

**Figure 10:**
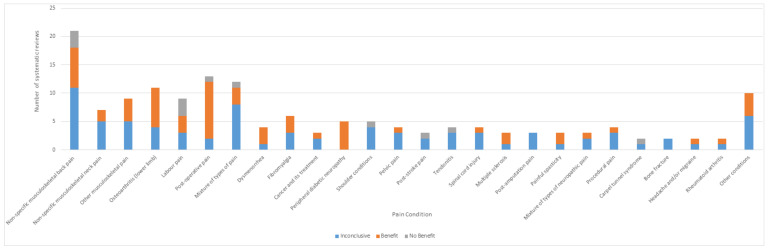
Conclusions of systematic reviews. Tally of our categorisation of conclusions of authors according to pain condition (*n* = 154).

The tally of the conclusions of authors of systematic reviews were inconclusive across the majority of pain conditions except for post-operative pain, osteoarthritis and peripheral diabetic neuropathy where the majority of authors judged evidence to tend toward benefit (Figure 10). However, when judged against our criteria, there were 151 systematic reviews with insufficient data and/or quality to make judgements about efficacy. The three systematic reviews with sufficient data provided evidence that TENS reduced chronic musculoskeletal pain [10], labour pain [26] and post-operative analgesic consumption [11].

#### 3.4.5. Meta-Analyses TENS versus Control for Pain Intensity (Continuous Data)

It was difficult to compare effect size estimates between reviews due to inconsistency in analysis methodologies.

Standardised mean differences are shown in Table 2. One of the SMDs did not include a comparator (i.e., pre-post TENS estimate for chronic back pain [52]). Visual inspection of the 19 SMD estimates versus a comparator revealed one idiosyncratic SMD estimate by Brosseau et al. [62] who reported an unusually high SMD relative to baseline for chronic low back pain (i.e., SMD = −4.32 (95% CI −10.36, −1.72) in favour of TENS). The remaining 18 estimates of SMD versus a comparator lay between −1.65 and 1.27; there were no noticeable differences in the magnitude of absolute SMDs and SMDs relative to baseline.

There were 12 of 19 SMDs where confidence intervals did not bisect the line of no difference suggesting greater efficacy for TENS compared with the comparator, irrespective of whether calculated as absolute differences or differences relative to baseline.

Inconsistency in analytical approaches made it difficult to compare SMD estimates across most pain conditions. All SMDs for pain associated with osteoarthritis and for post-operative pain had upper limit confidence intervals that were in favour of TENS (i.e., did not bisect the line of no difference). Only one of the four SMDs relative to baseline for chronic back pain had an upper confidence interval that was in favour of TENS (i.e., did not bisect the line of no difference).

Mean differences are shown in Table 3. All estimates of mean differences were against a comparator, although it was not possible to estimate data from some reports because findings were presented graphically without numerical equivalents. Estimates of MD versus a comparator lay between −26.3 and 0.59 relative to baseline and between −44.41 and 6.13 absolute difference on a 100 mm scale; there were no noticeable differences in the magnitude of absolute SMDs and SMDs relative to baseline.

There were 9/11 MDs where confidence interval did not bisect the line of no difference suggesting greater efficacy for TENS compared with the comparator, irrespective of whether calculated as absolute differences or differences relative to baseline. Inconsistency in analytical approaches made it difficult to compare MD estimates across most pain conditions.

**Figure 11:**
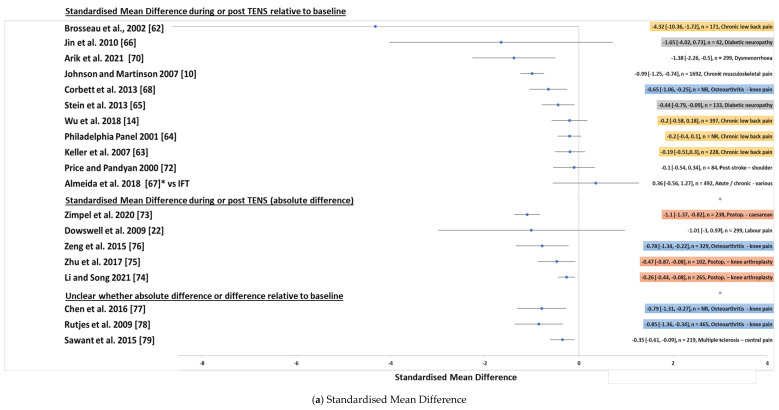

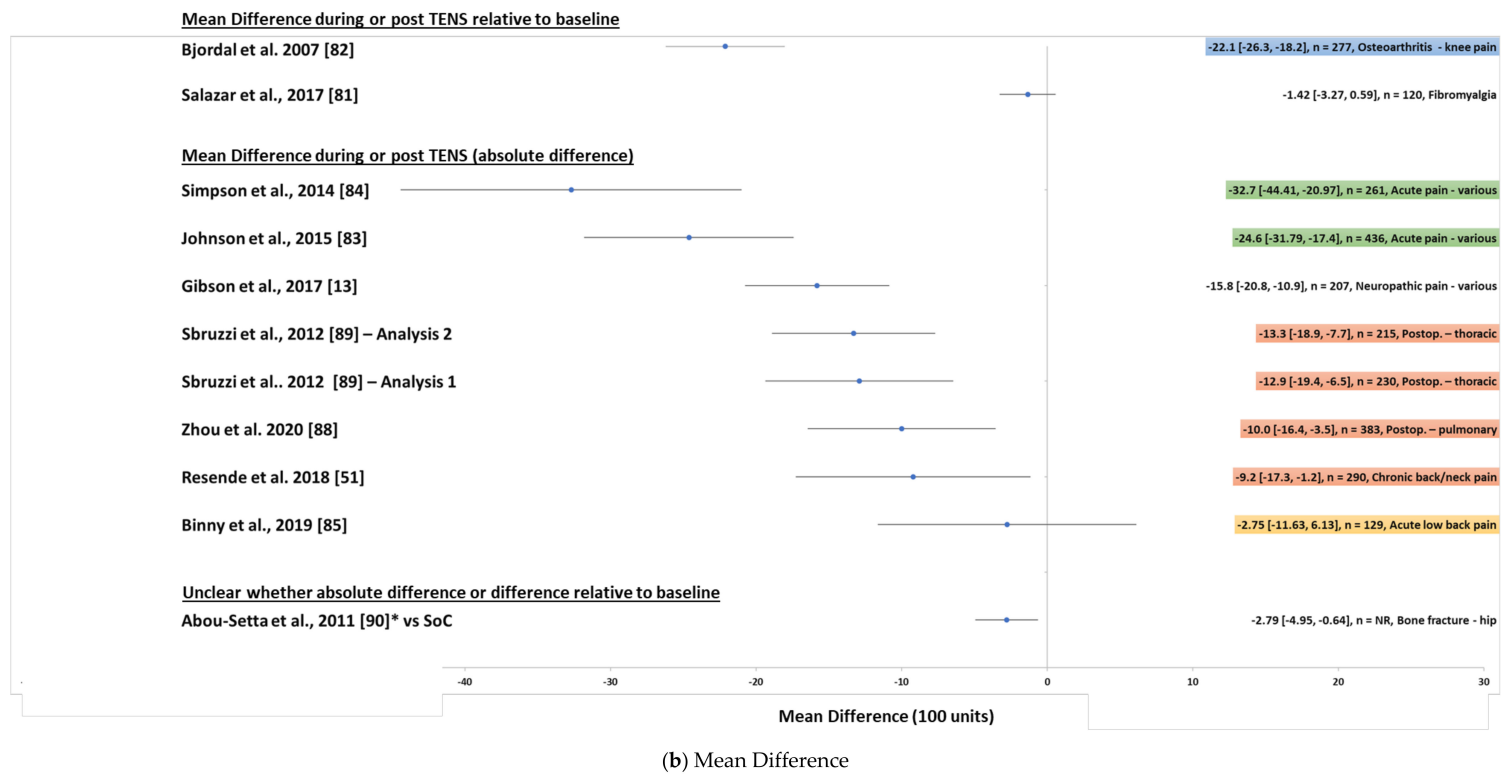
Plots of effect sizes and 95% confidence intervals for different pain conditions during or immediately post TENS. Overall effect size SoC = Standard of care; IFT = interferential therapy; * indicates not versus placebo control. Pain conditions have been highlighted in colour (right hand column).

A summary of effect size estimates grouped according to pain condition are shown in Figure 11a,b. Reporting of meta-analyses methods and findings was sometimes superficial, unclear and inconsistent. There were instances of authors presenting forest plots labelled as mean differences but stating standardised mean differences in figure captions and uncertainty whether MDs were representing scores on 100- or 10-unit scales.

There was one instance of inconsistency in reporting. Dowswell et al. [22] reported a SMD for the difference in pain intensity during/post between TENS and placebo/routine care of −1.01 (95% CI −3.0, 0.97) from data pooled from two studies (TENS = 143, placebo = 156, random effects model). In an updated report published two years later which was excluded from our review, the same data was pooled but reported the SMD was stated to be −0.16 (95% CI, −0.39, 0.07) possibly because they had calculated SMD using a fixed-effects rather than random effects model [23].

### 3.5. Analysis of Outcomes Specific for Specific Pain Conditions

In the following section we appraise outcomes according to specific pain conditions.

#### 3.5.1. Mixtures of Painful Conditions (15 Reviews)

We included 10 reviews that evaluated TENS for a mixture of types of chronic pain, three reviews that evaluated TENS for a mixture of types of acute pain, and two reviews that evaluated TENS for a mixture of types of acute and chronic pain (Table 4).

**Table 2:** Standardised mean differences reported in reviews and calculated from pooled data for pain intensity for continuous data.

Reference	Title	Condition	Comparison	No. Pooled Studies	Number of Participants Pooled TENS	Number of Participants Pooled Comparison	SMD	Lower CI	Upper CI	Comment
**Standardised Mean Difference during or post TENS relative to baseline**										
Wu et al., 2018 [14]	Literature Review and Meta-Analysis of Transcutaneous Electrical Nerve Stimulation in Treating Chronic Back Pain	Chronic low back pain	Control	9	TENS = 238	Control = 159	−0.2	−0.58	0.18	NOTE: Data also presented for SMD TENS vs. other nerve stimulation therapies = 0.86 (95%CI 0.15, 1.57), TENS = 122 NST = 105, 5 trials
Keller et al., 2007 [63]	Effect sizes of non-surgical treatments of non-specific low-back pain	Chronic low back pain	Placebo	2	Not reported Total sample = 114	Not reported Total sample = 114	−0.19	−0.51	0.13	NOTE: of the 2 studies one recorded improvement of pain on a 6 point Likert scale and the other pain intensity VAS
Philadelphia Panel 2001 [64]	Philadelphia Panel Evidence-Based Clinical Practice Guidelines on Selected Rehabilitation Interventions for Low Back Pain	Chronic low back pain	Unclear	3	Not reported	Not Reported	−0,2	−0,4	0.1	NOTE: MA for chronic back pain only. SMD reported but not trial arm sample sizes
Brosseau et al., 2002 [62]	Efficacy of the transcutaneous electrical nerve stimulation for the treatment of chronic low back pain—A meta-analysis.	Chronic low back pain	Placebo	3	TENS = 89	Placebo = 82	−4.32	−10.36	−1.72	NOTE: We used data from Figure 1 of the report at 1 month
Stein et al., 2013 [65]	Electrical stimulation and electromagnetic field use in patients with diabetic neuropathy: systematic review and meta-analysis	Diabetic neuropathy	Sham	5	TENS = 76	Sham TENS = 57	−0.44	−0.79	−0.09	
Jin et al., 2010 [66]	Effect of transcutaneous electrical nerve stimulation on symptomatic diabetic peripheral neuropathy: A meta-analysis of randomized controlled trials	Diabetic neuropathy	Sham	2	TENS = 26	Sham TENS = 16	−1.65	−4.02	0.73	NOTE: Forest plot has multiple counts from the same study. Figure within the report calculated an overall SMD with data extracted from different time points from the same study. We have extracted data at 12 weeks because other SMDs represented 1 study e.g., at 4 weeks SMD TENS vs. sham = −5.37 (95%CI −6.97, −3.77) pain intensity TENS = 18 sham = 13
Almeida et al., 2018 [67]	Transcutaneous electrical nerve stimulation and interferential current demonstrate similar effects in relieving acute and chronic pain: a systematic review with meta-analysis	Acute and chronic pain—various	IFT	8	TENS = 249	IFT = 243	0.36	−0.56	1.27	NOTE: Pain intensity VAS relative to baseline VAS, values pre-and post-intervention and results
Johnson and Martinson 2007 [10]	Efficacy of electrical nerve stimulation for chronic musculoskeletal pain: A meta-analysis of randomized controlled trials	Chronic musculoskeletal pain	Placebo	28	TENS/ES = 869	Placebo = 823	−0.99	−1.25	−0.74	NOTE: Forest plot has multiple counts from the same study. Figure 2 within the report estimated overall SMD using data extracted from different time points from the same study. Also includes PENS interventions and data duplicates in analysis
Corbett et al., 2013 [68]	Acupuncture and other physical treatments for the relief of pain due to osteoarthritis of the knee: network meta-analysis	Osteoarthritis—knee pain	Standard care	12	Nor reported	Not reported	−0.65	−1.06	−0.25	
Philadelphia Panel 2001 [69]	Clinical practice guidelines on selected rehabilitation interventions for knee pain	Osteoarthritis—knee pain	Placebo	5	TENS = 113	Placebo = 111	Not reported	Not reported	Not reported	NOTE: There is a forest plot but SMD not reported
Arik et al., 2021 [70]	The effect of TENS for pain relief in women with primary dysmenorrhea: A systematic review and meta-analysis	Dysmenorrhoea	Sham	2	TENS = 143	Sham TENS = 156	−1.38	−2.26	−0.5	
Cottrell et al., 2014 [71]	Benefits and Harms of Electrical Neuromodulation for Chronic Pelvic Pain: A Systematic Review	Chronic pelvic pain	Control	(a) 2 RCT (b) 4 nonRCT	Not reported (a) Total sample = 87 (b) Total sample = 131	Not reported (a) Total sample = 87 (b) Total sample = 131	Not reported	Not reported	Not reported	NOTE: Figure 2a from the report is a forest plot that provides pain scores in RCTs and (c) and Forest plot provides pain scores in non-RCTs. Neither states overall effect size for the TENS trials as overall effect size calculated from data pooled with other neuromodulation techniques
Price and Pandyan 2000 [72]	Electrical stimulation for preventing and treating post-stroke shoulder pain	Post-stroke—shoulder	Control	2	TENS = 46	Control = 38	−0.1	−0.54	0.34	NOTE: Extracted Electrical Stimulation (Functional electrical stimulation or TENS) vs. sham. There was only 1 SMD TENS vs. control = −0.44 (CI −1.05, −0.16), TENS = 26, control = 18
**Standardised Mean Difference during or post TENS (absolute difference)**										
Zimpel et al., 2020 [73]	Complementary and alternative therapies for post-caesarean pain	Postoperative pain—caesarean	Placebo	3	TENS = 119	Control = 119	−1.1	−1.37	−0.82	SMD TENS (+ analgesia) vs. placebo (+ analgesia) = −1.10 (CI −1.37, −0.82)
Li and Song 2021 [74]	Transcutaneous electrical nerve stimulation for postoperative pain control after total knee arthroplasty: A meta-analysis of randomized controlled trials	Postoperative pain—knee arthroplasty	Control	5	TENS = 136	Control = 131	−0.26	−0.44	−0.08	
Zhu et al., 2017 [75]	Effect of Transcutaneous Electrical Nerve Stimulation for Pain Control after Total Knee Arthroplasty: A Systematic Review and Meta-Analysis	Postoperative pain—knee arthroplasty	Control	2	TENS = 51	Control = 51	−0.47	−0.87	−0.08	
Zeng et al., 2015 [76]	Electrical stimulation for pain relief in knee osteoarthritis systematic review and network meta-analysis	Osteoarthritis—knee pain	Control	9	Not reported Total sample = 329	Not reported Total sample = 329	−0.78	−1.34	−0.22	NOTE: Used data for SMD h-TENS vs. control; Also reported: h-TENS vs. IFC = −0.14 (CI −1, 0.74), total sample = 56, 1 trial; h-TENS vs. l-TENS = −0.64 (CI −1.53, 0.32), total sample = 75. 2 trials; l-TENS vs. control = −0.14 (CI −1.03, 0.78), total sample = 123, 3 trials. This was a network meta-analysis.
Dowswell et al., 2009 [22]	Transcutaneous electrical nerve stimulation (TENS) for pain management in labour (Review)	Labour pain	Placebo/routine care	2	TENS = 143	Placebo/routine care = 156	−1.01	−3.0	0.97	NOTE: This is using the same study data as (Bedwell et al., 2011) but gets a different SMD. This used a random effects model
**Standardised Mean Difference—Unclear whether absolute difference or difference relative to baseline**										
Chen et al., 2016 [77]	Transcutaneous Electrical Nerve Stimulation in Patients with Knee Osteoarthritis Evidence from Randomized-controlled Trials	Osteoarthritis—knee pain	Control	12	Not reported	Not reported	−0.79	−1.31	−0.27	NOTE: Needed to manually calculate sample sizes. Exact time points for data extracted was unclear
Rutjes et al., 2009 [78]	Transcutaneous electrostimulation for osteoarthritis of the knee (Review)	Osteoarthritis—knee pain	Sham or no treatment	11	TENS = 275	Control = 190	−0.85	−1.36	−0.34	NOTE: Post, but when post was not available, they pooled DRB
Sawant et al., 2015 [79]	Systematic review of efficacy of TENS for management of central pain in people with multiple sclerosis	Multiple sclerosis—central pain	Control	4	TENS = 109	Control = 110	−0.35	−0.61	−0.09	
**Standardised Mean Difference—No comparator (i.e., pre-post only)**										
Jauregui et al., 2016 [52]	A Meta-Analysis of Transcutaneous Electrical Nerve Stimulation for Chronic Low Back Pain	Chronic low back pain	None	12	Not reported	No control	0.84	0.44	1.24	
Cherian et al., 2016 [80]	The effects of various physical non-operative modalities on the pain in osteoarthritis of the knee	Osteoarthritis—knee pain	None	7	TENS = 107	No control	1.702	1.17	2.23	

Key: IFT = interferential therapy; CI = 95% Confidence Interval DRB = difference relative to baseline, DAbs = absolute difference.

**Table 3:** Mean differences reported in reviews and calculated from pooled data for pain intensity for continuous data.

Reference	Title	Condition	Comparison	No. Pooled Studies	Number of Participants Pooled TENS	Number of Participants Pooled Comparison	Measure	MD	Lower CI	Upper CI	Comment
**Mean Difference during or post TENS relative to baseline**											
Salazar et al., 2017 [81]	Electric Stimulation for Pain Relief in Patients with Fibromyalgia: A Systematic Review and Meta-analysis of Randomized Controlled Trials	Fibromyalgia	Non-TENS	5	TENS = 63	Non-TENS = 57	Difference during/post TENS relative to baseline	−1.34	−3.27	0.59	Appears to be inconsistency in reporting of whether this is a mean difference or a standardised mean difference
Bjordal et al., 2007 [82]	Short-term efficacy of physical interventions in osteoarthritic knee pain. A systematic review and meta-analysis of randomised placebo-controlled trials.	Osteoarthritis—knee pain	Placebo	7	TENS (IFT) = 163	Placebo = 114	Difference during/post TENS relative to baseline	−22.1	−26.3	−18.12	
**Mean Difference during or post TENS (absolute difference)**											
Johnson et al., 2015 [83]	Transcutaneous electrical nerve stimulation for acute pain	Acute pain—various	Placebo	6	TENS = 218	Placebo 218	Absolute Difference during/post TENS	−24.6	−31.79	−17.4	
Simpson et al., 2014 [84]	Transcutaneous electrical nerve stimulation for relieving acute pain in the prehospital setting	Acute pain—various in prehospital setting	Sham	4	TENS = 128	Sham TENS = 133	Absolute Difference during/post TENS	−32.7	−44.41	−20.97	
Binny et al., 2019 [85]	Transcutaneous electric nerve stimulation (TENS) for acute low back pain: systematic review	Acute low back pain	Control	2	TENS = 64	Control = 65	Absolute Difference during/post TENS	−2.75	−11.63	6.13	
Resende et al., 2018 [51]	Meta-analysis of transcutaneous electrical nerve stimulation for relief of spinal pain	Chronic back and/or neck pain	Control	6	TENS/IFT 148	Control = 142	Absolute Difference during/post TENS	−9.2	−17.3	−1.2	
Machado et al., 2009 [86]	Analgesic effects of treatments for non-specific low back pain: a meta-analysis of placebo-controlled randomized trials	Chronic low back pain	Placebo	4	Not reported Total sample 178	Not reported Total sample 178	Absolute Difference during/post TENS	Not reported	Not reported	Not reported	NOTE: Data was pooled and forest plot presented without numbers. Effect size not reported
Poitras and Brosseau 2008 [87]	Evidence-informed management of chronic low back pain with transcutaneous electrical nerve stimulation, interferential current, electrical muscle stimulation, ultrasound, and thermotherapy	Chronic low back pain	Control	2	Not reported	Not reported	Absolute Difference during/post TENS	Not reported	Not reported	Not reported	NOTE: There is a forest plot but MD data not reported—Figure 1 from the report—we used HF TENS (*n* = 2 studies) rather than LF TENSA (*n* = 3 studies) MD for High frequency = 2 studies but MD data not given on figure
van Middelkoop et al., 2011 [53]	A systematic review on the effectiveness of physical and rehabilitation interventions for chronic non-specific low back pain	Chronic low back pain	Control	4	Not reported	Not reported	Absolute Difference during/post TENS	Not possible to isolate TENS effects	Not possible to isolate TENS effects	Not possible to isolate TENS effects	NOTE: Not possible to isolate effects due to TENS alone as TENS as part of a combination therapy of therapeutic ultrasound, low level laser and massage.
Gibson et al., 2017 [13]	Transcutaneous electrical nerve stimulation (TENS) for neuropathic pain in adults (Review)	Neuropathic pain—various	Sham	5	TENS = 111	Sham TENS = 96	Absolute Difference during/post TENS	−15.8	−20.8	−10.9	
Zhou et al., 2020 [88]	Efficacy of Transcutaneous Electronic Nerve Stimulation in Postoperative Analgesia After Pulmonary Surgery: A Systematic Review and Meta-Analysis	Postoperative pain—pulmonary surgery	Control	7	TENS = 193	Control = 190	Absolute Difference during/post TENS	−10	−16.4	−3.5	
Sbruzzi et al., 2012 [89]—Analysis 1 surgery with posterolateral thoracotomy approach	Transcutaneous electrical nerve stimulation after thoracic surgery: systematic review and meta-analysis of randomized trials	Postoperative pain—thoracic surgery	Sham	4	TENS = 117	Sham TENS = 113	Absolute Difference during/post TENS	−12.9	−19.4	−6.5	
Sbruzzi et al., 2012 [89]—Analysis 2 surgery with posterolateral thoracotomy approach	Transcutaneous electrical nerve stimulation after thoracic surgery: systematic review and meta-analysis of randomized trials	Postoperative pain—thoracic surgery	Control	6	TENS = 108	Control = 107	Absolute Difference during/post TENS	−13.3	−18.9	−7.7	
**Mean Difference—unclear whether absolute difference or difference relative to baseline**											
Abou-Setta et al., 2011 [90]	Comparative Effectiveness of Pain Management Interventions for Hip Fracture: A Systematic Review	Bone fracture—hip	Standard of care	2	Not reported	Not reported	Unclear	−2.79	−4.95	−0.64	
Malone and Strube 1988 [91]	Meta-analysis of non-medical treatments for chronic pain	Chronic pain—various	No treatment	2	Not reported	Not reported	Unclear	0.46	Not reported	Not reported	MD TENS vs. no treatment control = 0.46 (SD = 0.07)

Key: IFT = interferential therapy; CI = 95% Confidence Interval.

**Table 4:** Summary of reviews that include evaluation of TENS for a mixture of painful conditions. The column ‘Authors’ Conclusion’ contains statements taken from reports.

Ref.	Title	Condition	Acute/Chronic Pain	Review Type	Number of TENS Studies	Meta-Analysis	Authors’ Conclusion	Authors’ Judgement	Our Judgement	Comment
**Mixtures of different types of Chronic Pain**										
Gibson et al. [6]	Transcutaneous electrical nerve stimulation (TENS) for chronic pain—an overview of Cochrane Reviews (Review)	Chronic pain—various	Chronic	OCR	51	N	We were therefore unable to conclude with any confidence that, in people with chronic pain, TENS is harmful, or beneficial for pain control, disability, health-related quality of life, use of pain-relieving medicines, or global impression of change	?	+/−	The most comprehensive review without meta-analysis to date
Axon et al. [92]	Use of multidomain management strategies by community dwelling adults with chronic pain: evidence from a systematic review	Chronic pain—various	Chronic	SR	6	N	No statement of conclusion for TENS	?	?	
Baird et al. [93]	Interventions for treating persistent pain in survivors of torture	Chronic pain—various	Chronic	CR	1	N	No statement of conclusion for TENS	?	?	Only 1 RCT—TENS as part of combination therapy
Crawford et al. [94]	Physically Oriented Therapies for the Self-Management of Chronic Pain Symptoms	Chronic pain—various	Chronic	SR	2	N	…no recommendation could be made for or against the usage of TENS as a self-management technique for chronic pain symptoms without more research	?	?	
Park et al. [95]	Nonpharmacological Approaches to the Management of Chronic Pain in Community-Dwelling Older Adults: A Review of Empirical Evidence	Chronic pain—various	Chronic	SR	3	N	Although the findings of the effectiveness of TENS are inconsistent in the reviewed studies, there was a trend toward greater pain reduction with active TENS than with placebo or the combination of TENS and acupuncture	?	?	
Nnoham et al. [96]	TENS for chronic pain	Chronic pain—various	Chronic	CR	25	N	Despite the widespread use of TENS machines, the analgesic effectiveness of TENS still remains uncertain	?	?	Update of Carroll et al. [97] by different team so included
Claydon et al. [24]	Does transcutaneous electrical nerve stimulation (TENS) produce ‘dose-responses’? A review of systematic reviews on chronic pain	Chronic pain—various	Chronic	OSR	28	N	Data from chronic pain trials that use these outcome measures show that any dose related responses of TENS cannot be conclusively demonstrated as a result of the number of confounding variables (e.g., inadequate design, low statistical power and differences in TENS protocols)	?	?	[28 RCTs described in 6 SRs]
Carroll et al. [97]	Transcutaneous electrical nerve stimulation (TENS) for chronic pain	Chronic pain—various	Chronic	CR	19	N	There is insufficient evidence to draw any conclusions about the effectiveness of TENS for the treatment of chronic pain in adults	?	?	Updated in 2008 (Nnoaham and Kumbang, 2008) [19 RCTs from 18 reports]
Reeve et al. [98]	Transcutaneous electrical nerve stimulation (TENS): a technology assessment	Chronic pain—various	Chronic	SR	10	N	…there is little evidence of the effectiveness of TENS in treating chronic pain	?	?	One report containing 3 separate SRs
Malone et al. [91]	Meta-analysis of non-medical treatments for chronic pain	Chronic pain—various	Chronic	SR	7	Y	Effect sizes for operant training and TENS were no larger than the estimated effect size for control conditions	−	?	
**Acute Pain (Various) 3 reviews**										
Johnson et al. [83]	Transcutaneous electrical nerve stimulation for acute pain	Acute pain—various	Acute	CR	26	Y	The analysis provides tentative evidence that TENS reduces pain intensity over and above that seen with placebo (no current) TENS when administered as a stand-alone treatment for acute pain in adults	+	?	Comprehensive review—only assessed TENS as a stand-alone treatment
Simpson et al. [84]	Transcutaneous electrical nerve stimulation for relieving acute pain in the prehospital setting	Acute pain—various in prehospital setting	Acute	SR	4	Y	When administered by medics in the prehospital setting to patients with acute pain, TENS appears to be an effective and safe nonpharmacological analgesic modality that should be considered by emergency medical services organizations in which pharmacological pain management is restricted or unavailable	+	?	
Reeve et al. [98]	Transcutaneous electrical nerve stimulation (TENS): a technology assessment	Acute—various	Acute	SR	24	N	…published evidence is equivocal in acute pain treatment	?	?	One report containing 3 separate SRs
**Mixed Chronic/Acute Pain (Various) 2 reviews**										
Almeida et al. [67]	Transcutaneous electrical nerve stimulation and interferential current demonstrate similar effects in relieving acute and chronic pain: a systematic review with meta-analysis	Various—acute and chronic	Both	SR	8	Y	Transcutaneous electrical nerve stimulation and interferential current have similar effects on pain outcome	+	?	There was no comparison with control/placebo
Samuel et al. [99]	Application of Low Frequency and Medium Frequency Currents in the Management of Acute and Chronic Pain—A Narrative Review	Various—acute and chronic	Both	MR	3 9 SRs	N	We found through this review that even though TENS and IFT are used in management of pain, there is limited amount of high-quality research available…	?	?	

Key: OSR = overview of systematic reviews; SR = systematic review; CR = Cochrane review; MR = mixed review; Y = yes; *n* = no; The column ‘Authors’ judgement’: + = evidence tending to favour TENS, − = evidence tending not to favour TENS, ? = evidence tending to be conflicting, inconclusive or insufficient to make a judgement; The column ‘Our Judgement’: + = Sufficient evidence to judge—TENS beneficial; - = Sufficient evidence to judge—TENS not beneficial; +/− = Sufficient evidence to judge—inconclusive; ? = Insufficient evidence to judge.

#### Mixtures of Chronic Pain Conditions

There were two overviews of systematic reviews. In 2008, Claydon and Chesterton [24] conducted a review of six systematic reviews and suggested that three of these reviews provided evidence that TENS was superior to placebo for any type of chronic pain; and two of the six reviews found that high intensity TENS was more effective than low intensity TENS, when compared with placebo. Claydon and Chesterton [24] extracted information from 24 trials that compared TENS with placebo and judged 14 of these to show positive outcome, of which eight were deemed to be of high quality. In 2019, a comprehensive overview Cochrane overview of eight Cochrane reviews published in 2019 evaluated the efficacy of TENS for any type of chronic pain [6]. Gibson et al. [6] judged evidence to be inconclusive based on a descriptive analysis of 51 RCTs (with 2895 participants). The reviewers were reluctant to meta-analyse data due to methodological and clinical heterogeneity. Readers are directed to this overview as a comprehensive appraisal of evidence to date.

We included 12 systematic reviews and one hybrid review. The earliest attempt to meta-analyse data on TENS was published by Malone and Strube in 1988 [91] in a report that described an evaluation of non-medical treatments for chronic pain. They claimed that effect sizes for TENS were no larger than the estimated effect size for control conditions although this conclusion was based on pooling of insufficient data from two studies. The first systematic reviews that focused solely on TENS for chronic pain were conducted in the mid to late 1990s, including the first Cochrane review of TENS for chronic pain [97], and they revealed a plethora of issues that compromised methodological quality of RCTs on TENS that remain unresolved to the present time. The Cochrane review was updated by Khadilkar et al., in 2008 [100] and more recently converted into an overview of Cochrane reviews by Gibson et al., in 2019 [6] as described previously.

When judged against our criteria, there were no systematic reviews with sufficient data to make a judgement. Estimates of SMD or MD for TENS versus a control in meta-analyses are likely to be imprecise and uncertain. In summary, we judge that evidence within reviews is inconclusive. Recent NICE guidelines for chronic pain do not recommend that TENS should be offered for chronic primary pain.

#### Mixtures of Acute Pain Conditions

We included three systematic reviews. There were no overviews of systematic reviews or hybrid reviews. The earliest attempt to systematically review TENS for review a mixture of types of acute pain was published by Reeve et al., in 1996 [98], included predominantly post-operative pain and labour pain and was inconclusive. The most recent Cochrane review by Johnson et al., in 2015 [83] included studies that mostly evaluated TENS on various painful procedures including cervical laser surgery, venepuncture, and sigmoidoscopy as well as pain associated with post-partum uterine contractions and rib fractures and found tentative evidence of benefit. Reviews on specific painful procedural pains are discussed in Section 3.5.14.

When judged against our criteria, there were no systematic reviews with sufficient data to make a judgement and estimates of SMD or MD for TENS versus a control in meta-analyses are likely to be imprecise and uncertain. In summary, we judged evidence within reviews to be inconclusive. The NICE have not published guidelines for the management of acute pain per se but have published guidelines for management of acute pain for specific conditions.

### 3.5.2. Musculoskeletal Pain (40 Reviews)

We included 40 reviews that evaluated TENS for non-specific musculoskeletal pain, and we categorised two of these as acute non-specific back pain, 21 as chronic non-specific spinal back and/or neck pain, eight as non-specific neck pain (but not back) and nine as various types of chronic musculoskeletal pain (Table 5).

There was one overview of systematic reviews that did not include any data for TENS, although concluded that TENS could provide symptom relief of non-specific neck pain [56]; one mixed review that concluded that TENS was not shown to be effective for either chronic or subacute or acute low back pain based on four RCTs [61]; and one mixed review that summarised the Cochrane review by Khadilkar et al. [100] and concluded that there was no evidence to support efficacy of TENS for the treatment of non-specific chronic low back pain [111]. The systematic review by Johnson and Martinson in 2007 [10], evaluated TENS for a mixture of types of chronic non-specific musculoskeletal pain and is the largest meta-analysis of pooled data for TENS published to date. They pooled data from TENS and invasive peripheral nerve stimulation from a variety of different musculoskeletal conditions (29 RCTs, 32 comparisons) and found a significant reduction in pain during TENS compared with control [85]. The authors concluded that electrical nerve stimulation was effective treatment modality for chronic musculoskeletal pain. We judged there to be sufficient data to support efficacy for TENS against our criteria.

#### Non-Specific Back Pain (21 Reviews on Chronic, Two Reviews on Acute)

We included 21 systematic reviews of TENS for non-specific back pain and 2 hybrid reviews (Table 5). Good quality systematic reviews and meta analyses included Jauregui et al., published in 2016 [52], Wu et al., published in 2018 [14] and Resende et al., published in 2018 [51]. Jauregui et al. [52] suggested that their meta-analysis provided evidence that TENS reduced pain and medication; Wu et al. [14] that TENS improved functional disability but not lower back pain; and Resende et al. [51] that evidence was inconclusive based on large heterogeneity between studies. Their meta-analysis of 290 participants found that pain intensity was lower during TENS or interferential current therapy compared with placebo/control with an overall standardised mean difference of −0.92 (95% CI −1.73, −0.12; *p* < 0.02). The most recent Cochrane review by Khadilkar et al. [100], published in 2008, included five studies and concluded that evidence does not support the use of TENS in the routine management of chronic low back pain, although the review has been withdrawn and updated by the overview of Cochrane reviews for chronic pain by Gibson et al. [6]. When judged against our criteria, there were no systematic reviews with sufficient data to make a judgement. Also of note, was a comparison of the efficacy of 34 treatments for non-specific chronic low back pain published by Machado et al., in 2009 [86] which estimated that TENS reduced pain intensity between 10 and 20 percent of baseline and the magnitude of this effect was comparable with other treatments including muscle relaxants and non-steroidal anti-inflammatory drugs (NSAIDs).

In summary, the evidence suggests that there are insufficient high-quality RCTs to judge the efficacy of TENS for chronic non-specific back pain. The NICE guidelines for chronic non-specific back pain recommend that TENS should not be offered.

#### Non-Specific Neck Pain (Eight Reviews)

There was one overview of systematic reviews on a variety of treatments for non-specific neck pain published in 2007 by Jensen and Harms-Ringdahl [56] and it did not find any previous systematics reviews that focused solely on TENS (Table 5). The most robust systematic review was published in 2019 by Martimbianco et al. [125] and was an update of an earlier Cochrane review by Kroeling et al. [132]. Martimbianco et al. [125] included seven studies but did not undertake a meta-analysis and concluded that there was very low-certainty evidence from two trials about the effects of conventional TENS when compared to sham TENS at short-term.

When judged against our criteria, there were no systematic reviews with sufficient data to make a judgement. In summary, the evidence suggests that there are insufficient high-quality RCTs to judge the efficacy of TENS for non-specific neck pain.

### 3.5.3. Osteoarthritis (13 Reviews)

There were 11 systematic reviews and two overviews of systematic reviews that included evaluations of TENS for pain associated with osteoarthritis (Table 6). An overview of systematic reviews of physical therapy interventions for patients with osteoarthritis of the knee published in 2008 by Jamtvedt et al. [54] concluded that there is moderate-quality evidence that TENS reduced pain based on the findings of one Cochrane review on TENS by Osiri et al. [133] published in 2000. The development of Osteoarthritis Research Society International (OARSI) recommendations included a systematic review evidence of treatments to manage osteoarthritis of the hip and knee by Zhang et al. [55] but did not include any systematic reviews or studies on TENS. Nevertheless, Zhang et al. [55] concluded that TENS can help with short-term pain control in some patients.

There were nine systematic reviews included in our appraisal. The most recent Cochrane review published in 2009 by Rutjes et al. [78] included 12 studies on TENS, including interferential current therapy, and “could not confirm that transcutaneous electrostimulation is effective for pain relief” (Abstract). In 2016, Chen et al. [77] published a review of 23 studies on TENS for knee osteoarthritis and concluded that TENS might relieve pain due to knee osteoarthritis based on a meta-analysis that did not report the size of the pooled data sample. In 2017, Ferronato et al. [134] published a systematic review of physical modalities on functional performance in knee osteoarthritis and concluded that “…*TENS seems to be as or more effective than other analgesic therapies*”.

When judged against our criteria, there were no systematic reviews with sufficient data to judge the efficacy of TENS. The NICE guidelines recommend that TENS should be offered for as an adjunct to core treatment to manage pain associated with osteoarthritis [135].

### 3.5.4. Post-Operative Pain (13 Reviews)

There were 13 systematic reviews but no overviews or hybrid reviews evaluating TENS for post-operative pain (Table 7). One of the first systematic reviews of TENS for post-operative pain was published by Carroll et al. [5] in 1996 and remains a seminal piece of work. Carroll et al. [5] tallied study outcome and found no difference in pain intensity between TENS and sham TENS in 14 of 17 RCTs. In contrast, 17 of 19 non-randomised studies reported lower pain intensity for TENS compared with sham TENS. Carroll et al., concluded that TENS did not alleviate post-operative pain and that non-randomized studies overestimated treatment effects.

In 2003, Bjordal et al. [11] argued that measurements of pain intensity may be compromised when participants are concurrently consuming analgesic medication. Bjordal et al. [11] conducted a meta-analysis of 21 RCTs that found a mean reduction in analgesic consumption after TENS to be 26.5% (range 6% to 51%) when compared with placebo. Importantly, TENS technique moderated the effect with optimal reduction in analgesic consumption occurring in the presence of strong, sub noxious TENS sensation at the site of pain. When judged against our criteria, this review had sufficient data to support benefit of TENS for reducing opioid consumption.

**Table 6:** Summary of reviews that include evaluation of TENS for pain associated with osteoarthritis (OA). The column ‘Authors’ Conclusion’ contains statements taken from reports.

Ref.	Title	Condition	Acute/Chronic Pain	Review Type	Number of TENS Studies	Meta-Analysis	Authors’ Conclusion	Authors’ Judgement	Our Judgement	Comment
**OA Knee 10 reviews**										
Ferronato et al. [134]	Physical modalities on the functional performance in knee osteoarthritis: a systematic review	Osteoarthritis—knee	Chronic	SR	13	N	The use of TENS seems to be as or more effective than other analgesic therapies. From 11 articles included in this review, 10 articles evaluated the beneficial effects of TENS and 1 article didn’t notice increases in comparison with the placebo group	+	?	
Chen et al. [77]	Transcutaneous Electrical Nerve Stimulation in Patients with Knee Osteoarthritis Evidence from Randomized-controlled Trials	Osteoarthritis—knee	Chronic	SR	23	Y	TENS might relieve pain due to knee osteoarthritis. Further randomized-controlled trials should focus on large-scale studies and a longer duration of follow-up.	?	?	
Cherian et al. [80]	The effects of various physical non-operative modalities on the pain in osteoarthritis of the knee	Osteoarthritis—knee	Chronic	SR	7	Y	In conclusion, all four non-operative modalities reduced the pain of OA of the knee: neuromuscular electrical stimulation and TENS were the most effective.	+	?	
Zeng et al. [76]	Electrical stimulation for pain relief in knee osteoarthritis systematic review and network meta-analysis	Osteoarthritis—knee	Chronic	SR	12	Y	…the recommendation level of the other electrical stimulation therapies is either uncertain (h-TENS) or not appropriate (l-TENS…) for pain relief…Interferential currents seems to be the most promising pain relief treatment for the management of knee OA	?	?	
Corbett et al. [68]	Acupuncture and other physical treatments for the relief of pain due to osteoarthritis of the knee: network meta-analysis	Osteoarthritis—knee	Chronic	SR	18	Y	End of treatment results showed that eight interventions: interferential therapy, acupuncture, TENS, pulsed electrical stimulation, balneotherapy, aerobic exercise, sham acupuncture, and muscle-strengthening exercise produced a statistically significant reduction in pain when compared with standard care…Our analyses found little evidence (of significant differences from standard care, let alone clinically-relevant differences) to support such guidance with respect to treating pain, other than for TENS, where the evidence was of poor quality and likely to be unreliable.	+	?	
Rutjes et al. [78]	Transcutaneous electrostimulation for osteoarthritis of the knee (Review)	Osteoarthritis—knee	Chronic	CR	12	Y	…we could not confirm that TENS is effective for pain relief. The current systematic review is inconclusive, hampered by the inclusion of only small trials of questionable quality	?	?	Update of Osiri et al. [133]. We included both reviews because conducted by different teams. There were 4 studies on interferential therapy included in the analysis
Jamtvedt et al. [54]	Physical therapy interventions for patients with osteoarthritis of the knee: an overview of systematic reviews.	osteoarthritis—knee	Chronic	OSR	0 1 SR	N	There is moderate-quality evidence that acupuncture, TENS, and low-level laser therapy reduce pain….	+	?	
Bjordal et al. [82]	Short-term efficacy of physical interventions in osteoarthritic knee pain. A systematic review and meta-analysis of randomised placebo-controlled trials.	osteoarthritic knee pain	Chronic	SR	11	Y	TENS, electroacupuncture and low level laser therapy administered with optimal doses in an intensive 2–4 week treatment regimen, seem to offer clinically relevant short-term pain relief for OAK	+	?	
Philadelphia Panel [69]	Clinical practice guidelines on selected rehabilitation interventions for knee pain	Knee pain—various	Both	SR	6	Y	TENS and therapeutic exercises were beneficial for knee osteoarthritis…TENS for Post-surgery Rehabilitation, Level I (RCT), Grade C for Pain (No Evidence of Clinically Important Benefit)	+	?	
Osiri et al. [133]	Transcutaneous electrical nerve stimulation for knee osteoarthritis (Review)	Osteoarthritis—knee	Chronic	CR	9	Y	TENS and AL-TENS are shown to be effective in pain control over placebo in this review.	+	?	Subsequently updated by Rutjes et al.,(Rutjes et al., 2009)
**OA knee and hip 3 reviews**										
Zhang et al. [55]	OARSI recommendations for the management of hip and knee osteoarthritis, Part I: Critical appraisal of existing treatment guidelines and systematic review of current research evidence	Osteoarthritis—knee and hip	Chronic	OSR	0	N	TENS can help with short-term pain control in some patients with hip or knee OA	+	?	Conclusion based on expert consensus rather than systematic review evidence
Brosseau et al. [136]	Efficacy of transcutaneous electrical nerve stimulation for osteoarthritis of the lower extremities: A meta-analysis	Osteoarthritis—Knee and hip	Chronic	SR	6	N	All modes of TENS (CTENS, ALTENS, BTENS, and MIX TENS) showed a significant benefit for pain relief in the treatment of osteoarthritis involving the knee and/or hip. This was true regardless of duration and repetition of intervention.	+	?	
Puett et al. [137]	Published trials of nonmedicinal and non-invasive therapies for hip and knee osteoarthritis	Osteoarthritis—knee and hip	Chronic	SR	3	N	More data are needed to…evaluate the role of topical capsaicin, laser therapy, acupuncture, TENS, and pulsed electromagnetic fields.	?	?	

Key: OSR = overview of systematic reviews; SR = systematic review; CR = Cochrane review; MR = mixed review; Y = yes; *n* = no; The column ‘Authors’ judgement’: + = evidence tending to favour TENS, − = evidence tending not to favour TENS, ? = evidence tending to be conflicting, inconclusive or insufficient to make a judgement; The column ‘Our Judgement’: + = Sufficient evidence to judge—TENS beneficial; − = Sufficient evidence to judge—TENS not beneficial; +/− = Sufficient evidence to judge—inconclusive; ? = Insufficient evidence to judge.

**Table 7:** Summary of reviews that include evaluation of TENS for pain associated with post-operative pain. The column ‘Authors’ Conclusion’ contains statements taken from reports.

Ref.	Title	Condition	Acute/Chronic Pain	Review Type	Number of TENS Studies	MA	Authors’ Conclusion	Authors’ Judgement	Our Judgement	Comment
Zimpel et al. [73]	Complementary and alternative therapies for post-caesarean pain	Postoperative pain—caesarean	Acute	CR	10	Y	TENS plus analgesia, compared with placebo plus analgesia, may reduce pain, heart rate and respiratory rate…	+	?	
Zhou et al. [88]	Efficacy of Transcutaneous Electronic Nerve Stimulation in Postoperative Analgesia After Pulmonary Surgery: A Systematic Review and Meta-Analysis	Postoperative pain—pulmonary	Acute	SR	10	Y	Transcutaneous electronic nerve stimulation might be an effective supplementary analgesic regimen in multimodal analgesia to decrease pain intensity after pulmonary surgery	+	?	
Terracina et al. [138]	Prevention and Treatment of Postoperative Pain after Lumbar Spine Procedures: A Systematic Review	Postoperative—lumbar	Acute	SR	2	N	No statement of conclusion for TENS	?	?	
Yue et al. [139]	Systematic Review of Three Electrical Stimulation Techniques for Rehabilitation After Total Knee Arthroplasty	Postoperative pain—knee arthroplasty	Acute	SR	7	N	As adjunct modalities, neuromuscular electrical stimulation and TENS can effectively improve rehabilitation after total knee arthroplasty	+	?	Analysis of post-operative and short-term rehabilitation to 12 weeks
Zhu et al. [75]	Effect of TENS for pain control after post op knee arthroplasty	Postoperative pain—knee arthroplasty	Acute	SR	6	Y	Compared with control intervention, TENS supplementation intervention was found to significantly reduce pain and morphine requirement over a period of 24 h and to promote functional recovery in patients who have undergone total knee arthroplasty	+	?	
Li et al. [74]	Transcutaneous electrical nerve stimulation for postoperative pain control after total knee arthroplasty: A meta-analysis of randomized controlled trials.	Postoperative pain—knee arthroplasty	Acute	SR	5	Y	TENS could significantly reduce pain and opioid consumption after total knee arthroplasty. In addition, there were fewer adverse effects in the TENS groups	+	?	
Kerai et al. [140]	Role of transcutaneous electrical nerve stimulation in post-operative analgesia	Postoperative pain	Acute	SR	8	N	Most of the studies have demonstrated clinically significant reduction in pain intensity and supplemental analgesic requirement. However, these trials vary in TENS parameters used that is, duration, intensity, frequency of stimulation and location of electrodes.	+	?	
Caley et al. [141]	The effects of transcutaneous electrical nerve stimulation (TENS) in postoperative cardiothoracic pain: a systematic review.	Postoperative cardio-thoracic pain cardiothoracic	Acute	SR	4	N	All studies concluded TENS to have no adverse effects and to be beneficial in post-operative pain…firm conclusions on the use of TENS in this setting cannot be made from this systematic review	?	?	
Sbruzzi et al. [89]	Transcutaneous electrical nerve stimulation after thoracic surgery: systematic review and meta-analysis of randomized trials	Postoperative pain—thoracic	Acute	SR	11	Y	TENS associated with pharmacological analgesia provides pain relief compared to the placebo TENS in postoperative thoracic surgery patients both approached by thoracotomy and sternotomy. In sternotomy it also provides more effective pain relief compared to pharmacological analgesia alone, but it has no significant effect on pulmonary function	+	?	Includes two meta-analyses for different surgeries but does not combine as one
Freynet et al. [142]	Is transcutaneous electrical nerve stimulation effective in relieving postoperative pain after thoracotomy?	Postoperative pain—thoracotomy	Acute	SR	9	N	Hence, current evidence shows TENS associated with postoperative medications to be safe and effective in alleviating postoperative pain and in improving patient recovery, thus enhancing the choice of available medical care and bettering outcome after thoracic surgery	+	?	
Sabino et al. [143]	Transcutaneous Electrical Nerve Stimulation in Thoracic or Abdominal Postoperative Conditions	Postoperative pain—thoracic and abdominal	Acute	SR	6	N	TENS demonstrated specific effectiveness for different outcomes. The results of this systematic review presented no evidences to recommend or reject the use of TENS for functional recovery in the postoperative period. The use of distinct TENS parameters, chosen in a random and unjustified form, made it impossible to determine optimal stimulation patterns	+	?	
Bjordal et al. [11]	Transcutaneous electrical nerve stimulation (TENS) can reduce postoperative analgesic consumption A meta-analysis with assessment of optimal treatment parameters for postoperative pain.	Postoperative pain—various	Acute	SR	21	Y	TENS, administered with a strong, subnoxious intensity at an adequate frequency in the wound area, can significantly reduce analgesic consumption for postoperative pain	+	+	
Carroll et al. [5]	Randomization is important in studies with pain outcomes: systematic review of transcutaneous electrical nerve stimulation in acute postoperative pain	Postoperative pain—various	Acute	SR	17	N	Fourteen of the 17 included RCTs compared TENS with sham TENS; no differences were found. In 17 of these 19 [non-randomised] TENS studies, the authors concluded that TENS had appositive analgesic effect.	−	?	Non-randomized studies overestimated treatment effects. Some of this data also reported by McQuay et al. [3]

Key: OSR = overview of systematic reviews; SR = systematic review; CR = Cochrane review; MR = mixed review; Y = yes; *n* = no; The column ‘Authors’ judgement’: + = evidence tending to favour TENS, − = evidence tending not to favour TENS, ? = evidence tending to be conflicting, inconclusive or insufficient to make a judgement; The column ‘Our Judgement’: + = Sufficient evidence to judge—TENS beneficial; − = Sufficient evidence to judge—TENS not beneficial; +/− = Sufficient evidence to judge—inconclusive; ? = Insufficient evidence to judge.

More recently, meta-analyses have suggested that TENS is of benefit at relieving postoperative pain after thoracotomy and sternotomy [89,142], total knee arthroplasty [139], pulmonary surgery [88] and post-caesarean pain [73], although none of these had sufficient data to make a judgement according to our criteria. Zimpel et al. [73] claimed that TENS plus analgesia (versus placebo plus analgesia) reduced the intensity of post-caesarean pain (visual analogue scale) at one hour with a SMD of −1.10 (95% CI −1.37 to −0.82; 3 studies; 238 women; low-certainty evidence). In summary, the evidence suggests that TENS is of benefit for postoperative pain.

### 3.5.5. Labour Pain (10 Reviews)

There were 10 reviews that included an evaluation for TENS for labour (Table 8). The earliest reviews of TENS for labour pain published in the mid-1990s were robust and reported that TENS did not alleviate pain nor reduce analgesic consumption [98,144]. In 2012, Jones et al. [25] published an overview of Cochrane reviews that evaluated pain management for women in labour including TENS and concluded that there was insufficient evidence to make a judgement based on one Cochrane review published by Dowswell et al. [22] in 2009. Dowswell et al. [22] included 18 studies and conducted a meta-analysis that pooled data two studies resulting in 143 participants in the TENS arm and 156 in the placebo/routine care arm with the SMD estimated to be −1.01 (95% CI, −3.00, 0.97) for mean pain intensity during labour.

A report by the same team published three years later by Bedwell et al. [23] presented the same analysis, but SMD was reported to be −0.16 (95% CI −0.39, 0.07) for mean pain intensity during labour. We suspect that the substantial difference in the magnitude of the effect sizes estimates is due to reporting the findings of a random effects model by Dowswell et al. [22] and a fixed effects model by Bedwell et al. [23]. Dowswell et al. [22] reported a risk ratio of severe pain during labour between TENS and placebo/routine care of 0.67 (95% CI, 0.32, 1.40, random effects model) based on pooling of 79 events in the TENS arm and 68 events in the placebo/routine care arm, whereas Bedwell et al. [23] a risk ratio of 0.77 (95% CI, 0.60, 1.00, *p* = 0.05). Again, we suspect the discrepancies in values to be due to the use of a fixed effect model in the latter analysis. This demonstrates the problem of discrepancies in effect size estimates reported within and between investigators, although this did not affect conclusions.

In 2020, Thuvarakan et al. [26] published a systematic review of 26 randomized controlled trials (3348 parturients) and a meta-analysis of 700 parturients in the TENS arm and 626 parturients in the control arm that found a small but statistically significant reduction in pain intensity during TENS with the risk ratio for participants experiencing moderate (>30%) or a strong reduction in pain intensity (>50%) as 1.52 (95% CI, 1.35, 1.70) in favour of TENS, although the quality of studies was low. When judged against our criteria, this analysis provided sufficient data to support the efficacy of TENS.

Despite widespread use of TENS for pain during the early stages of childbirth, the NICE recommend that TENS should not be offered to women in established labour, although it may be beneficial in the early stages of labour [145].

**Table 8:** Summary of reviews that include evaluation of TENS for pain associated with childbirth (i.e., labour pain). The column ‘Authors’ Conclusion’ contains statements taken from reports.

Ref.	Title	Condition	Acute/Chronic Pain	Review Type	Number of TENS Studies	MA	Authors’ Conclusion	Authors’ Judgement	Our Judgement	Comment
Thuvarakan et al. [26]	Transcutaneous electrical nerve stimulation as a pain-relieving approach in labor pain: A systematic review and Meta-Analysis of randomized controlled trials	Labour pain	Acute	SR	26	Y	The forest plot showed a small, but statistically significant efficacy of TENS on the reduction of pain intensity. However, it is not clear if the results were affected by the poor quality of the studies. This systematic review is the first that shows the application of TENS has significant efficacy in lowering labor pain	+	+	
Melo et al. [146]	Non-pharmacological resources: performance of physiotherapy in labor, a systematic review	Labour pain	Acute	SR	3	N	The studies suggest that the physiotherapy techniques investigated, for the most part, contributed in a beneficial way to relieving the pain of parturients…However, some findings have demonstrated inconclusive results about the effectiveness of techniques such as TENS, acupuncture, walking and breathing exercises	?	?	
Liddle et al. [147]	Interventions for preventing and treating low-back and pelvic pain during pregnancy.	Labour pain—low-back and pelvic pain	Acute	CR	1	N	There was low-quality evidence from one study by Keskin et al. [148]; *n* = 79 analysed) that TENS improved pain and functional disability significantly more than usual prenatal care	?	?	
Mafetoni et al. [149]	Non-pharmacological methods for pain releife during labour: Integrative review. Revista Mineira de Enfermagem, 18, 513–520.	Labour pain	Acute	SR	4	N	The use of TENS, for example, took place in the beginning of the first phase of labor, increasing pain tolerance; walking and/or the practice of keep the parturient in vertical position, showed to be an important strategy for pain relief, although it has been described that pain scores are higher according to the evolution of cervical dilation.	+	?	
Jones et al. [25]	Pain management for women in labour: an overview of systematic reviews.	Labour pain	Acute	OSR	22 1 CR	N	There is insufficient evidence to make judgements on whether or not hypnosis, biofeedback, sterile water injection, aromatherapy, TENS, or parenteral opioids are more effective than placebo or other interventions for pain management in labour]	?	?	A Cochrane overview of SRs not focussed on TENS
Mello et al. [150]	Transcutaneous electrical stimulation for pain relief during labor: a systematic review and meta-analysis	Labour pain	Acute	SR	9	Y	The use of TENS had no impact on mother or child and no influence on labor. According to the results of this review, there is no evidence that TENS reduces the use of additional analgesia.	−	?	
Dowswell et al. [22]	Transcutaneous electrical nerve stimulation (TENS) for pain management in labour (Review)	Labour pain	Acute	CR	18	Y	There is only limited evidence that TENS reduces pain in labour and it does not seem to have any impact (either positive or negative) on other outcomes for mothers or babies	?	?	This review was discussed in a subsequent report by Bedwell et al. [23] in which effect size estimates differed but did not affect the outcome
Simkin et al. [151]	Update on nonpharmacologic approaches to relieve labor pain and prevent suffering	Labour pain	Acute	SR	3	N	TENS provides modest pain relief benefits and is a satisfying option for most women who use it. Its efficacy in relieving back pain deserves further study	+	?	
Carroll et al. [144]	Transcutaneous electrical nerve stimulation does not relieve labor pain: updated systematic review	Labour pain	Acute	SR	10	Y	The findings of this review suggest that TENS has no significant effect on pain in labour	−	?	Update of Carroll et al. [152] and data also reported in McQuay et al. [3]
Reeve et al. [98]	Transcutaneous electrical nerve stimulation (TENS): a technology assessment.	Labour pain	Acute	SR	12	N	The bulk of evidence in labour and delivery indicates that TENS is not effective	−	?	One report containing 3 separate SRs

Key: OSR = overview of systematic reviews; SR = systematic review; CR = Cochrane review; MR = mixed review; Y = yes; *n* = no; The column ‘Authors’ judgement’: + = evidence tending to favour TENS, − = evidence tending not to favour TENS, ? = evidence tending to be conflicting, inconclusive or insufficient to make a judgement; The column ‘Our Judgement’: + = Sufficient evidence to judge—TENS beneficial; − = Sufficient evidence to judge—TENS not beneficial; +/− = Sufficient evidence to judge—inconclusive; ? = Insufficient evidence to judge.

### 3.5.6. Dysmenorrhea (Seven Reviews) and Pelvic Pain (Four Reviews)

There were 11 reviews that evaluated TENS for dysmenorrhea and/or pelvic pain (Table 9). There were seven reviews on dysmenorrhea (one of these was a Consensus Guideline [58]) and four reviews on a mixture of types of pelvic pain (some included dysmenorrhea). There were three hybrid reviews on TENS for dysmenorrhea [58,59,60]. In 2002, Proctor et al. [153] published a Cochrane review on TENS for primary dysmenorrhoea that included seven studies and claimed that high-frequency TENS was found beneficial, but there was insufficient evidence to determine the effectiveness of low-frequency TENS. In 2021, Arik et al. [70] published a systematic review and meta-analysis that included only four studies and concluded that TENS may be beneficial to alleviate pain in primary dysmenorrhea. It is interesting that the more recent review included fewer studies. We judged there to be insufficient data to make a judgement from all of the reviews on dysmenorrhea to date.

The most robust systematic review for chronic pelvic pain was published by Cottrell et al. [71] in 2020 and concluded that TENS was beneficial for women with chronic pelvic pain secondary to dysmenorrhea. Cottrell et al. [71] reported the overall effect size for data extracted for various neuromodulation treatments including percutaneous tibial nerve stimulation and transcutaneous interferential electrical stimulation that included 2 RCTs on TENS (87 participants), but did not report the effect size for TENS per se. Cottrell et al., conducted a separate analysis that pooled data from various neuromodulation techniques that included 4 non-RCTs on TENS (131 participants), but did not report the effect estimate for TENS per se. The 95% confidence intervals for the effect size estimates of all TENS studies were in favour of TENS and did not cross the line of no difference, although according to our criteria there was insufficient data to make a judgement about efficacy. In 2018, Franco et al. [154] published a Cochrane review of non-pharmacological interventions for treating chronic prostatitis/chronic pelvic pain syndrome that included 2 studies on TENS, although the authors judged evidence to be inconclusive.

In summary, there are insufficient high-quality RCTs to judge the efficacy of TENS for dysmenorrhea or chronic pelvic pain.

### 3.5.7. Fibromyalgia (Six Reviews)

There were six reviews on fibromyalgia (Table 10). The most recent systematic review without meta-analysis was published in 2019 by Megia Garcia et al. [155] who claimed that TENS was beneficial for reducing pain in fibromyalgia, especially when added to therapeutic exercise, based on an evaluation of eight studies.

A Cochrane review of eight studies by Johnson et al. [156] was inconclusive due to insufficient high-quality evidence. A meta-analysis of five studies published by Salazar et al. [81] in 2017 concluded that electrical stimulation (using TENS or electroacupuncture) relieved pain in patients with fibromyalgia, although there were fewer than 200 participants were pooled and the sensitivity analysis suggested that TENS showed no effect. When judged against our criteria, none of these systematic reviews had sufficient data to make a judgement. Fibromyalgia is considered to be a chronic primary pain. The NICE guidelines for chronic primary pain do not recommend that TENS should be offered [157].

**Table 9:** Summary of reviews that include evaluation of TENS for pain associated with dysmenorrhea and pelvic pain. The column ‘Authors’ Conclusion’ contains statements taken from reports.

Ref.	Title	Condition	Acute/Chronic Pain	Review Type	Number of TENS Studies	Meta-Analysis	Authors’ Conclusion	Authors’ Judgement	Our Judgement	Comment
**Pelvic pain**										
Cottrell et al. [71]	Benefits and Harms of Electrical Neuromodulation for Chronic Pelvic Pain: A Systematic Review	Pelvic pain	Chronic	SR	12	Y	TENS has been shown to be an effective treatment for women with chronic pelvic pain secondary to dysmenorrhea and is free from adverse events, with the advantage that it can be self-applied and cost effective	+	?	Review included studies on dysmenorrhoea
Franco et al. [154]	Non-pharmacological interventions for treating chronic prostatitis/chronic pelvic pain syndrome	Pelvic pain	Chronic	CR	2	Y	We were uncertain about the effects of…TENS,…	?	?	
Cheong et al. [158]	Non-surgical interventions for the management of chronic pelvic pain	Pelvic pain	Chronic	CR	0	N	No statement of conclusion for TENS	?	?	
Cohen et al. [159]	Therapeutic intervention for chronic prostatitis/chronic pelvic pain syndrome (CP/CPPS): a systematic review and meta-analysis.	Pelvic pain	Chronic	SR	0	N	No statement of conclusion for TENS	?	?	
**Dysmenorrhea**										
Arik et al. [70]	The effect of TENS for pain relief in women with primary dysmenorrhea: A systematic review and meta-analysis	Dysmenorrhea	Acute	SR	4	Y	TENS is a safe and well tolerated electrophysical therapy that may be effective for relieving pain in primary dysmenorrhea	+	?	
Burnett et al. [58]	No. 345-Primary Dysmenorrhea Consensus Guideline.	Dysmenorrhea	Acute	MR	1 1 SR	N	High-frequency TENS should be considered as a complementary treatment or in women unable or unwilling to use conventional therapy (II-1B)…	+	?	
Igwea et al. [160]	TENS and heat therapy for pain relief and quality of life improvement in individuals with primary dysmenorrhea: A systematic review	Dysmenorrhea	Acute	SR	6	N	TENS and heat therapy show potential as adjunct remedies in the management of primary dysmenorrhea, but rigorous high-quality trials are still needed to make conclusive recommendation	?	?	
Kannan et al. [161]	Some physiotherapy treatments may relieve menstrual pain in women with primary dysmenorrhea: a systematic review	Dysmenorrhea	Acute	SR	1	N	Physiotherapists could consider using heat, TENS, and yoga in the management of primary dysmenorrhea	+	?	
Latthe et al. [59]	Dysmenorrhea	Dysmenorrhea	Acute	MR	1	N	High-frequency TENS may reduce pain compared with sham TENS but seems to be less effective than ibuprofen	+	?	
Proctor and Farquhar [60]	Dysmenorrhea	Dysmenorrhea	Acute	MR	2 SRs	N	High-frequency TENS reduces pain compared with placebo TENS (moderate-quality evidence). We don’t know whether low-frequency TENS reduces pain compared with placebo TENS (low-quality evidence). The effectiveness of TENS is unclear compared with NSAIDs…(very low-quality evidence).	+	?	
Proctor et al. [153]	Transcutaneous electrical nerve stimulation for primary dysmenorrhoea (Review)	Dysmenorrhoea	Acute	CR	7	Y	High-frequency TENS was found to be effective for the treatment of dysmenorrhoea by a number of small trials. The minor adverse effects reported in one trial require further investigation. There is insufficient evidence to determine the effectiveness of low-frequency TENS in reducing dysmenorrhoea	+	?	Subsequently updated as a MR by Proctor and Farquhar [60]

Key: OSR = overview of systematic reviews; SR = systematic review; CR = Cochrane review; MR = mixed review; Y = yes; *n* = no; The column ‘Authors’ judgement’: + = evidence tending to favour TENS, − = evidence tending not to favour TENS, ? = evidence tending to be conflicting, inconclusive or insufficient to make a judgement; The column ‘Our Judgement’: + = Sufficient evidence to judge—TENS beneficial; − = Sufficient evidence to judge—TENS not beneficial; +/− = Sufficient evidence to judge—inconclusive; ? = Insufficient evidence to judge.

**Table 10:** Summary of reviews that include evaluation of TENS for pain associated with fibromyalgia. The column ‘Authors’ Conclusion’ contains statements taken from reports.

Ref.	Title	Condition	Acute/Chronic Pain	Review Type	Number of TENS Studies	MA	Authors’ Conclusion	Authors’ Judgement	Our Judgement	Comment
Megia Garcia et al. [155]	Analgesic effects of transcutaneous electrical nerve stimulation (TENS) in patients with fibromyalgia A systematic review	Fibromyalgia	Chronic	SR	8	N	Treatment with TENS is effective for reducing pain in people with fibromyalgia. In addition, the inclusion of TENS in therapeutic exercise programs seems to have a greater effect than practicing therapeutic exercise in isolation. Further studies are needed to investigate the optimization of the parameters of the TENS and a greater consensus among the variables used.	+	?	
Honda et al. [162]	Effects of Physical-Agent Pain Relief Modalities for Fibromyalgia Patients: A Systematic Review and Meta-Analysis of Randomized Controlled Trials	Fibromyalgia	Chronic	SR	1	N	TENS significantly reduced visual analogue scale scores.…Effect of electromagnetic therapy and TENS for the treatment of fibromyalgia on pain intensity was observed.	+	?	
Ibanez-Vera et al. [163]	Passive physiotherapy for the treatment of the syndrome of fibromyalgia. A systematic review	Fibromyalgia	Chronic	SR	4	N	The quality of their subjects with fibromyalgia seems to be improving with…TENS,…with a limited number of studies	+	?	
Johnson et al. [156]	Transcutaneous electrical nerve stimulation (TENS) for fibromyalgia in adults (Review)	Fibromyalgia	Chronic	CR	8	N	There was insufficient high-quality evidence to support or refute the use of TENS for fibromyalgia	?	?	
Salazar et al. [81]	Electric Stimulation for Pain Relief in Patients with Fibromyalgia: A Systematic Review and Meta-analysis of Randomized Controlled Trials	Fibromyalgia	Chronic	SR	6	Y	Our meta-analyses showed that electrical stimulation (electroacupuncture + TENS) in comparison with a control group seems be effective for pain relief in patients with fibromyalgia. Additionally, when we performed sensitivity analysis of the type of intervention, electroacupuncture presented favorable results toward the experimental group regarding pain relief, while TENS showed no effect.	?	?	Not sure whether MD relative to baseline in MA was in cm or mm
Ricci et al. [164]	The use of electrothermal and phototherapeutic methods for the treatment of fibromyalgia syndrome: a systematic review	Fibromyalgia	Chronic	SR	1	N	[One study] had positive outcomes after applying TENS for pain control, depression and quality of life in FMS patients, despite the fact that the sample of this study was not representative	?	?	

Key: OSR = overview of systematic reviews; SR = systematic review; CR = Cochrane review; MR = mixed review; Y = yes; *n* = no; The column ‘Authors’ judgement’: + = evidence tending to favour TENS, − = evidence tending not to favour TENS, ? = evidence tending to be conflicting, inconclusive or insufficient to make a judgement; The column ‘Our Judgement’: + = Sufficient evidence to judge—TENS beneficial; − = Sufficient evidence to judge—TENS not beneficial; +/− = Sufficient evidence to judge—inconclusive; ? = Insufficient evidence to judge.

### 3.5.8. Specific Shoulder Conditions (Five Reviews)

There were five systematic reviews and we judged them all to have insufficient evidence (Table 11). In 2016, Page et al. [165] published a Cochrane review of electrotherapy modalities for rotator cuff disease that included eight studies on TENS that was inconclusive based on a descriptive analysis and concluded that without meta-analysis. Each of the other systematic reviews only included one study of TENS.

### 3.5.9. Cancer and Its Treatment (Five Reviews)

There were five reviews and we judged them all to have insufficient evidence (Table 12). There were two overviews of systematic reviews [166,167], and both were inconclusive basing their conclusions on the findings of the most recent Cochrane review by Hurlow et al. [168] published in 2012 that included two very small studies on TENS. Thus, there are insufficient high-quality RCTs to judge the efficacy of TENS for cancer and its treatment.

### 3.5.10. Peripheral Diabetic Neuropathy (Five Reviews)

There were five reviews on painful peripheral diabetic neuropathy (Table 13). The most recent review published Zeng et al. [169] was published in 2020 and included seven studies and a meta-analysis on that pooled data from various peripheral electrical stimulation techniques that failed to find differences with controls. However, the authors claimed that their subgroup analysis demonstrated “…*a large effect for one of its subgroups (electrical peripheral techniques, predominantly TENS)*…”, although the effect size for TENS per se was not reported and pooled samples did not reach our threshold for sufficient data to make a judgement. Overall, none of the reviews provided sufficient data to have confidence in judgements.

### 3.5.11. Tendinitis/Tendinopathy (Four Reviews)

There were four reviews on tendinitis/tendinopathy at the elbow or shoulder and we judged them all to have insufficient evidence (Table 14). The largest review was published in 2016 by Desmeules et al. [170] and included six studies on TENS for rotator cuff tendinopathy with evidence judged by the reviewers as insufficient and inconclusive.

### 3.5.12. Post-Stroke Pain (4 Reviews)

There were four reviews and we judged them all to have insufficient evidence (Table 15). A Cochrane review published in 2000 by Price and Pandyan [72] on electrical stimulation for preventing and treating post-stroke shoulder pain included four studies on TENS and did not confirm or refute that TENS around the shoulder reduced pain after stroke. The most recent review was published in 2016 by Chen et al. [171] and included only one study on TENS and concluded that the strength of evidence for benefit was ‘poor’.

### 3.5.13. Spinal Cord Injury (Four Reviews)

There were four reviews and we judged them all to have insufficient evidence (Table 16). In 2016, Harvey et al. [172] published a systematic review on the effectiveness of 22 commonly administered physiotherapy interventions for people with spinal cord injury and found only two studies on TENS. Harvey et al. [172] judged the strength of evidence as low yet concluded that TENS was ‘clearly effective’. In 2014, Boldt et al. [173] published a Cochrane review on non-pharmacological interventions for chronic pain in people with spinal cord injury was inconclusive.

**Table 11:** Summary of reviews that include evaluation of TENS for pain specific shoulder pain. The column ‘Authors’ Conclusion’ contains statements taken from reports.

Ref.	Title	Condition	Acute/Chronic Pain	Review Type	Number of TENS Studies	Meta-Analysis	Authors’ Conclusion	Authors’ Judgement	Our Judgement	Comment
Hawk et al. [174]	Systematic Review of Nondrug, Nonsurgical Treatment of Shoulder Conditions	Shoulder Impingement	?	SR	1	N	Evidence was inconclusive because of the scarcity of studies	?	?	
Haik et al. [175]	Effectiveness of physical therapy treatment of clearly defined subacromial pain: a systematic review of randomised controlled trials	Subacromial pain	Both	SR	1	N	Microwave diathermy and TENS do not seem to be beneficial in SAPS [subacromial pain] treatment. The evidence is still low due to the low number of participants and studies available in the literature	−	?	
Page et al. [176]	Electrotherapy modalities for rotator cuff disease (Review)	Rotator cuff disease	Both	CR	8	N	We are uncertain whether TENS is superior to placebo, and whether any electrotherapy modality provides benefits over other active interventions (e.g., glucocorticoid injection) because of the very low quality of the evidence. due to the high risk of performance and detection bias (downgraded by two points) and imprecision (downgraded by one point)	?	?	
Page et al. [177]	Electrotherapy modalities for adhesive capsulitis (frozen shoulder)	Adhesive capsulitis	Both	CR	1	N	Overall, based on very low-quality evidence, we are uncertain whether a combination of therapeutic ultrasound, TENS and hot packs is an effective adjunct to exercise	?	?	
Johansson et al. [178]	A combination of systematic review and clinicians’ beliefs in interventions for subacromial pain	Subacromial pain	UC	SR	1	N	…there is no available evidence for efficacy of TENS for patients with subacromial pain	?	?	

Key: OSR = overview of systematic reviews; SR = systematic review; CR = Cochrane review; MR = mixed review; Y = yes; *n* = no; The column ‘Authors’ judgement’: + = evidence tending to favour TENS, − = evidence tending not to favour TENS, ? = evidence tending to be conflicting, inconclusive or insufficient to make a judgement; The column ‘Our Judgement’: + = Sufficient evidence to judge—TENS beneficial; − = Sufficient evidence to judge—TENS not beneficial; +/− = Sufficient evidence to judge—inconclusive; ? = Insufficient evidence to judge.

**Table 12:** Summary of reviews that include evaluation of TENS for pain associated with cancer and its treatment. The column ‘Authors’ Conclusion’ contains statements taken from reports.

Ref.	Title	Condition	Acute/Chronic Pain	Review Type	No. TENS Studies	MA	Authors’ Conclusion	Authors’ Judgement	Our Judgement	Comment
Wu et al. [166]	Effectiveness of acupuncture and related therapies for palliative care of cancer: overview of systematic reviews	Cancer	Both	OSR	0	N	No statement of conclusion for TENS	?	?	
Bao et al. [167]	Complementary and Alternative Medicine for Cancer Pain: An Overview of Systematic Reviews.	Cancer pain	Chronic	OSR	1 CR	N	Based on available evidence, we could find that…TENS,…might have beneficial effects on adult cancer pain…results were inconsistent for…TENS…plus cancer treatment	?	?	
Hökkä et al. [179]	A systematic review: non-pharmacological interventions in treating pain in patients with advanced cancer	Cancer	Chronic	SR	1	N	With just one limited study, it is not possible to draw conclusions about the safety and potential of TENS to reduce pain	?	?	
Hurlow et al. [168]	Transcutaneous electric nerve stimulation (TENS) for cancer pain in adults (Review)	Cancer pain	Chronic	CR	2	N	…the results of this updated systematic review remain inconclusive due to a lack of suitable RCTs	?	?	Updated review of Robb et al. [180]
Pan et al. [181]	Complementary and Alternative Medicine in the Management of Pain, Dyspnea, and Nausea and Vomiting Near the End of Life: A Systematic Review	Cancer pain	Chronic	SR	1	N	Case series and a small RCT suggest that TENS may provide short-term pain relief in dying patients or in patients with intractable cancer pain. TENS may provide short-term pain relief in patients with intractable or advanced cancer pain	+	?	

Key: OSR = overview of systematic reviews; SR = systematic review; CR = Cochrane review; MR = mixed review; Y = yes; *n* = no; The column ‘Authors’ judgement’: + = evidence tending to favour TENS, − = evidence tending not to favour TENS, ? = evidence tending to be conflicting, inconclusive or insufficient to make a judgement; The column ‘Our Judgement’: + = Sufficient evidence to judge—TENS beneficial; − = Sufficient evidence to judge—TENS not beneficial; +/− = Sufficient evidence to judge—inconclusive; ? = Insufficient evidence to judge.

**Table 13:** Summary of reviews that include evaluation of TENS for pain associated with peripheral diabetic neuropathy. The column ‘Authors’ Conclusion’ contains statements taken from reports.

Ref.	Title	Condition	Acute/Chronic Pain	Review Type	Number of TENS Studies	Meta-Analysis	Authors’ Conclusion	Authors’ Judgement	Our Judgement	Comment
Zeng et al. [169]	Non-invasive neuromodulation effects on painful diabetic peripheral neuropathy: a systematic review and meta-analysis	Diabetic peripheral neuropathy	Chronic	SR	7	Y	We found a consistent medium to large effect size on pain reduction by central techniques, but no significant effects for the overall peripheral techniques, although we found a large effect for one of its subgroups (electrical peripheral techniques, predominantly TENS)	+	?	
Stein et al. [65]	Electrical stimulation and electromagnetic field use in patients with diabetic neuropathy: systematic review and meta-analysis	Diabetic peripheral neuropathy	Both	SR	5	Y	We found that TENS improved pain relief in patients with diabetic neuropathy, while no such improvement was observed with the use of electromagnetic field treatment. The limited number of studies…demonstrate the need for further randomized clinical trials.	+	?	
Dubinsky et al. [115]	Assessment: Efficacy of transcutaneous electric nerve stimulation in the treatment of pain in neurologic disorders (an evidence-based review)	Diabetic peripheral neuropathy	Chronic	SR	3	N	TENS should be considered in the treatment of painful diabetic neuropathy (Level B)	+	?	Conducted two analyses in same report—this is data for peripheral diabetic neuropathy
Jin et al. [66]	Effect of transcutaneous electrical nerve stimulation on symptomatic diabetic peripheral neuropathy: A meta-analysis of randomized controlled trials	Diabetic peripheral neuropathy	Chronic	SR	3	Y	TENS therapy may be an effective and safe strategy in treatment of symptomatic diabetic peripheral neuropathy	+	?	
Pieber et al. [182]	Electrotherapy for the treatment of painful diabetic peripheral neuropathy: a review	Diabetic peripheral neuropathy	Chronic	SR	5	N	…the effects of TENS are consistent. The beneficial effects of prolonged use have been reported in three large studies and one small study.—TENS may be recommended for the treatment of PN.	+	?	

Key: OSR = overview of systematic reviews; SR = systematic review; CR = Cochrane review; MR = mixed review; Y = yes; *n* = no; The column ‘Authors’ judgement’: + = evidence tending to favour TENS, − = evidence tending not to favour TENS, ? = evidence tending to be conflicting, inconclusive or insufficient to make a judgement; The column ‘Our Judgement’: + = Sufficient evidence to judge—TENS beneficial; − = Sufficient evidence to judge—TENS not beneficial; +/− = Sufficient evidence to judge—inconclusive; ? = Insufficient evidence to judge.

**Table 14:** Summary of reviews that include evaluation of TENS for tendinitis/tendinopathy. The column ‘Authors’ Conclusion’ contains statements taken from reports.

Ref.	Title	Condition	Acute/Chronic Pain	Review Type	Number of TENS Studies	Meta-Analysis	Authors’ Conclusion	Authors’ Judgement	Our Judgement	Comment
Dion et al. [183]	Are passive physical modalities effective for the management of common soft tissue injuries of the elbow? A systematic review by the Ontario Protocol for Traffic Injury Management (OPTIMa) Collaboration	Tendinitis/Soft tissue injuries—elbow	UC	SR	2	N	…TENS provides no added benefit to patients with lateral epicondylitis. We found evidence from one low risk of bias RCT that TENS is not effective for the management of lateral epicondylitis	−	?	
Wu et al. [184]	Comparative Effectiveness of Nonoperative Treatments for Chronic Calcific Tendinitis of the Shoulder: A Systematic Review and Network Meta-Analysis of Randomized Controlled Trials	Tendinitis—Shoulder, Chronic Calcific	Chronic	SR	1	Y	Compared with low-energy focused extracorporeal shockwave therapy, TENS, and ultrasound therapy, H-FSW is the best therapy for providing functional recovery	?	?	
Desmeules et al. [170]	Efficacy of transcutaneous electrical nerve stimulation for rotator cuff tendinopathy: a systematic review	Tendinopathy—rotator cuff	UC	SR	6	N	…no conclusions can be drawn on the efficacy of TENS for the treatment of rotator cuff tendinopathy	?	?	We categorised this study as tendinopathy rather than specific shoulder
Dingemanse et al. [185]	Evidence for the effectiveness of electrophysical modalities for treatment of medial and lateral epicondylitis: a systematic review	Lateral epicondylitis	Chronic	SR	2	N	…evidence of no difference in the effect of electrotherapy versus placebo was found	?	?	(Update of Dingemanse et al. [185])

Key: OSR = overview of systematic reviews; SR = systematic review; CR = Cochrane review; MR = mixed review; Y = yes; *n* = no; The column ‘Authors’ judgement’: + = evidence tending to favour TENS, − = evidence tending not to favour TENS, ? = evidence tending to be conflicting, inconclusive or insufficient to make a judgement; The column ‘Our Judgement’: + = Sufficient evidence to judge—TENS beneficial; − = Sufficient evidence to judge—TENS not beneficial; +/− = Sufficient evidence to judge—inconclusive; ? = Insufficient evidence to judge.

**Table 15:** Summary of reviews that include evaluation of TENS for post stroke pain. The column ‘Authors’ Conclusion’ contains statements taken from reports.

Ref.	Title	Condition	Acute/Chronic Pain	Review Type	Number of TENS Studies	Meta-Analysis	Authors’ Conclusion	Authors’ Judgement	Our Judgement	Comment
Chen et al. [171]	The antalgic effects of non-invasive physical modalities on central post-stroke pain: a systematic review	Post-stroke pain	Chronic	SR	1	N	…the strength for its efficacy was poor and it was only effective for some of central post-stroke pain patients	?	?	
Ramos-Valero et al. [186]	Physiotherapy treatments for patients with shoulder pain after stroke. A systematic review	Post stroke pain—shoulder	Both	SR	1	N	These results indicate that this [TENS] technique does not only deal with the symptoms…	−	?	
Barreca et al. [57]	Interventions for the paretic upper limb of stroke survivors:	Post stroke pain	Chronic	MR	4 1 SR	N	…careful handling, electrical stimulation, movement with elevation, strapping, and the avoidance of overhead pulleys could effectively reduce or prevent pain in the paretic upper limb	+	?	This systematic review focussed on motor impairment after stroke
Price and Pandyan [72]	Electrical stimulation for preventing and treating post-stroke shoulder pain	Post-stroke pain—shoulder	Both	CR	4	Y	The evidence…does not confirm or refute that electrical stimulation around the shoulder after stroke influences reports of pain, but there do appear to be benefits for passive humeral lateral rotation	?	?	

Key: OSR = overview of systematic reviews; SR = systematic review; CR = Cochrane review; MR = mixed review; Y = yes; *n* = no; The column ‘Authors’ judgement’: + = evidence tending to favour TENS, − = evidence tending not to favour TENS, ? = evidence tending to be conflicting, inconclusive or insufficient to make a judgement; The column ‘Our Judgement’: + = Sufficient evidence to judge—TENS beneficial; − = Sufficient evidence to judge—TENS not beneficial; +/− = Sufficient evidence to judge—inconclusive; ? = Insufficient evidence to judge.

**Table 16:** Summary of reviews that include evaluation of TENS for spinal cord injury. The column ‘Authors’ Conclusion’ contains statements taken from reports.

Ref.	Title	Condition	Acute/Chronic Pain	Review Type	Number of TENS Studies	Meta-Analysis	Authors’ Conclusion	Authors’ Judgement	Our Judgement	Comment
Harvey et al. [172]	The effectiveness of 22 commonly administered physiotherapy interventions for people with spinal cord injury: a systematic review	Spinal cord injury	Chronic	SR	2	Y	…four interventions were clearly effective: fitness, hand and wheelchair training as well as TENS; however, the strength of evidence was not high	+	?	
Boldt et al. [173]	Non-pharmacological interventions for chronic pain in people with spinal cord injury	Spinal cord injury—chronic	Chronic	CR	1	N	…Insufficient evidence…Trials using…TENS…provided no evidence that these interventions reduce chronic pain.	?	?	
Mehta et al. [187]	Neuropathic Pain Post Spinal Cord Injury Part 1: Systematic Review of Physical and Behavioral Treatment	Spinal cord injury—neuropathic pain	Chronic	SR	2	N	…there is conflicting evidence that TENS treatment reduces neuropathic pain post spinal cord injury	?	?	
Fattal et al. [188]	What is the efficacy of physical therapeutics for treating neuropathic pain in spinal cord injury patients?	Spinal cord injury—neuropathic	Unclear	SR	2	N	No statement of conclusion for TENS	?	?	

Key: OSR = overview of systematic reviews; SR = systematic review; CR = Cochrane review; MR = mixed review; Y = yes; *n* = no; The column ‘Authors’ judgement’: + = evidence tending to favour TENS, − = evidence tending not to favour TENS, ? = evidence tending to be conflicting, inconclusive or insufficient to make a judgement; The column ‘Our Judgement’: + = Sufficient evidence to judge—TENS beneficial; − = Sufficient evidence to judge—TENS not beneficial; +/− = Sufficient evidence to judge—inconclusive; ? = Insufficient evidence to judge.

### 3.5.14. Procedural Pain (Four Reviews)

There were four reviews and we judged them all to have insufficient evidence (Table 17). Cochrane reviews evaluating TENS for oocyte retrieval [189], pain during orthodontic treatment and amniocentesis or chorionic villus sampling [190] failed to find any studies. A review on TENS for discomfort during shockwave lithotripsy [191] included only one study.

### 3.5.15. Neuropathic Pain (Three Reviews)

There were three reviews on neuropathic pain in adults and we judged them all to have insufficient evidence (Table 18). In 2007, the European Federation of Neurological Societies (EFNS) published guidelines on neurostimulation therapy for neuropathic pain based on a systematic evaluation of nine studies on TENS and claimed that high-frequency TENS may be better than placebo and worse than electroacupuncture [20]. Interestingly, TENS was not in scope when the EFNS updated these guidelines in 2016 [21]. The most robust review to date was published in 2017 by Gibson et al. [13] that evaluated TENS for neuropathic pain in adults with a descriptive analysis of 15 studies that was inconclusive.

Undertaking reviews based on symptomology rather than medical diagnoses can challenging. For example, studies of painful conditions traditionally considered as nociceptive (non-neuropathic) may include participants with neuropathic pain elements, yet search strategies used in systematic reviews tend to exclude such conditions. Furthermore, studies of conditions traditionally considered as neuropathic, which are included in reviews on neuropathic pain, may include participants who do not present with neuropathic pain and therefore reviewers need to ensure that eligibility criteria take account of this, perhaps by including criteria that all participants exceeded a threshold for the presence of symptoms of neuropathic pain through screening.

### 3.5.16. Multiple Sclerosis (Three Reviews)

There were three reviews and we judged them all to have insufficient evidence (Table 19). However, multiple sclerosis presents with a variety of painful symptoms resulting from multiple causes.

Amatya et al. [192] evaluated TENS for low back pain whereas Sawant et al. [79] and Jawahar et al. [193] evaluated TENS on central neuropathic pain associated with abnormal sensibility.

The Cochrane review by Amatya et al. [192] published in 2018 on non-pharmacological interventions for chronic pain in multiple sclerosis, included one study evaluating TENS for low back pain [37] that was judged to be very low-level evidence of benefit. Sawant et al. [79] published a systematic review and meta-analysis in 2015 that included four studies and claimed to provide Grade 2 level evidence that TENS was beneficial and safe for central pain. Interestingly, Sawant et al. [79] included three small pilot studies from the same investigating team evaluating TENS for low back pain [37,194,195], raising issues about violating unit of analysis criteria.

**Table 17:** Summary of reviews that include evaluation of TENS for procedural pain. The column ‘Authors’ Conclusion’ contains statements taken from reports.

Ref.	Title	Condition	Acute/Chronic Pain	Review Type	Number of TENS Studies	Meta-Analysis	Authors’ Conclusion	Authors’ Judgement	Our Judgement	Comment
Kwan et al. [189]	Pain relief for women undergoing oocyte retrieval for assisted reproduction.	Procedural pain	Acute	CR	0	N	No statement of conclusion for TENS	?	?	Update of Kwan et al. [196]
Fleming et al. [197]	Non-pharmacological interventions for alleviating pain during orthodontic treatment	Orthodontic treatment—pain	Both	CR	0	N	No statement of conclusion for TENS	?	?	
Ngee-Ming et al. [191]	Complementary approaches to decreasing discomfort during shockwave lithotripsy (SWL)	Procedural pain	Acute	SR	1	N	…methods such as acupuncture, TENS and music offer an avenue to these benefits	+	?	
Mujezinovic et al. [190]	Analgesia for amniocentesis or chorionic villus sampling	Procedural pain	Acute	CR	0	N	No statement of conclusion for TENS	?	?	

Key: OSR = overview of systematic reviews; SR = systematic review; CR = Cochrane review; MR = mixed review; Y = yes; *n* = no; The column ‘Authors’ judgement’: + = evidence tending to favour TENS, − = evidence tending not to favour TENS, ? = evidence tending to be conflicting, inconclusive or insufficient to make a judgement; The column ‘Our Judgement’: + = Sufficient evidence to judge—TENS beneficial; − = Sufficient evidence to judge—TENS not beneficial; +/− = Sufficient evidence to judge—inconclusive; ? = Insufficient evidence to judge.

**Table 18:** Summary of reviews that include evaluation of TENS for neuropathic pain. The column ‘Authors’ Conclusion’ contains statements taken from reports.

Ref.	Title	Condition	Acute/Chronic Pain	Review Type	Number of TENS Studies	Meta-Analysis	Authors’ Conclusion	Authors’ Judgement	Our Judgement	Comment
Gibson et al. [13]	Transcutaneous electrical nerve stimulation (TENS) for neuropathic pain in adults (Review)	Neuropathic pain—various	Chronic	CR	15	Y	…we were unable to confidently state whether TENS is effective for pain control in people with neuropathic pain. The very low quality of evidence means we have very limited confidence in the effect estimate reported; the true effect is likely to be substantially different	?	?	Robust review
Pittler et al. [198]	Complementary therapies for neuropathic and neuralgic pain: systematic review	Neuropathic pain—various	UC	SR	3	N	…evidence can be classified as encouraging and warrants further study for…electrostimulation	?	?	Included transcutaneous or percutaneous electrical nerve stimulation
Cruccu et al. [20]	EFNS guidelines on neurostimulation therapy for neuropathic pain	Neuropathic pain—various	UC	SR	9	N	High-frequency TENS may be better than placebo (level C) although worse than electroacupuncture (level B)	+	?	

Key: OSR = overview of systematic reviews; SR = systematic review; CR = Cochrane review; MR = mixed review; Y = yes; *n* = no; The column ‘Authors’ judgement’: + = evidence tending to favour TENS, − = evidence tending not to favour TENS, ? = evidence tending to be conflicting, inconclusive or insufficient to make a judgement; The column ‘Our Judgement’: + = Sufficient evidence to judge—TENS beneficial; − = Sufficient evidence to judge—TENS not beneficial; +/− = Sufficient evidence to judge—inconclusive; ? = Insufficient evidence to judge.

**Table 19:** Summary of reviews that include evaluation of TENS for multiple sclerosis. The column ‘Authors’ Conclusion’ contains statements taken from reports.

Ref.	Title	Condition	Acute/Chronic Pain	Review Type	Number of TENS Studies	MA	Authors’ Conclusion	Authors’ Judgement	Our Judgement	Comment
Amatya et al. [192]	Non-pharmacological interventions for chronic pain in multiple sclerosis	Multiple sclerosis—chronic pain	Chronic	CR	2	N	There is very low-level evidence for the use of non-pharmacological interventions for chronic pain such as TENS,…in pain intensity of persons with multiple sclerosis	?	?	Update of Amatya et al. [199]
Sawant et al. [79]	Systematic review of efficacy of TENS for management of central pain in people with multiple sclerosis	Multiple sclerosis—central pain	Chronic	SR	4	Y	TENS is a safe and effective non-pharmacological alternative in the management of central pain in people living with multiple sclerosis. These findings are consistent with GRADE 2 level of evidence	+	?	
Jawahar et al. [193]	Central neuropathic pain in multiple sclerosis	Multiple sclerosis—neuropathic pain	Chronic	SR	2	N	TENS may be effective in reducing central neuropathic pain…	+	?	

Key: OSR = overview of systematic reviews; SR = systematic review; CR = Cochrane review; MR = mixed review; Y = yes; *n* = no; The column ‘Authors’ judgement’: + = evidence tending to favour TENS, − = evidence tending not to favour TENS, ? = evidence tending to be conflicting, inconclusive or insufficient to make a judgement; The column ‘Our Judgement’: + = Sufficient evidence to judge—TENS beneficial; − = Sufficient evidence to judge—TENS not beneficial; +/− = Sufficient evidence to judge—inconclusive; ? = Insufficient evidence to judge.

### 3.5.17. Painful Spasticity (Three Reviews)

There were three reviews and we judged them all to have insufficient evidence (Table 20). In 2016, Mills and Dossa [200] published a systematic review and descriptive analysis of 14 studies that they claimed provided level 1 and 2 evidence that TENS improves spasticity-related outcomes, especially when TENS was used in combination with exercise and task-related training. In 2019, Fernandez-Tenorio et al. [201] published a systematic review of 10 studies that provided evidence that TENS may be beneficial for painful spasticity. A Cochrane review evaluating TENS for spasticity following traumatic brain injury was inconclusive [202].

### 3.5.18. Post-Amputation Pain (Three Reviews)

There were three reviews we judged them all to have insufficient evidence (Table 21). A Cochrane review published in 2015 by Johnson et al. [203] found no RCTs and only two small non RCT studies, and two non-Cochrane systematic reviews included only three small studies.

### 3.5.19. Conditions with Two Reviews

We found two reviews for each of the following conditions and we judged them all to have insufficient evidence: rheumatoid arthritis, headache or migraine, carpal tunnel syndrome, and fracture pain (Table 22).

We were surprised at how few reviews had been conducted on TENS for rheumatoid arthritis and that they were published nearly two decades ago; one review claimed evidence supported beneficial effects [204] and a Cochrane review was inconclusive [205]. Interestingly, NICE guidelines recommend that patients have access to specialist physiotherapy to learn about the short-term pain relief provided by methods such as TENS [206]. A systematic review of four studies on TENS for the treatment of migraine published in 2018 by Tao et al. [207] included a meta-analysis that was claimed to provide low quality evidence that TENS may be beneficial and well-tolerated treatment for migraine.

### 3.5.20. Conditions with One Review

We found one review for a variety of painful conditions (Table 23), and we judged them all to have insufficient evidence of benefit. Of note was a systematic review on physiotherapy for pain and disability in adults with complex regional pain syndrome (CRPS) types I and II that included six studies on TENS, although reviewers concluded that evidence was absent or unclear [208].

**Table 20:** Summary of reviews that include evaluation of TENS for painful spasticity. The column ‘Authors’ Conclusion’ contains statements taken from reports.

Ref.	Title	Condition	Acute/Chronic Pain	Review Type	Number of TENS Studies	MA	Authors’ Conclusion	Authors’ Judgement	Our Judgement	Comment
Fernandez-Tenorio et al. [201]	Transcutaneous electrical nerve stimulation for spasticity: A systematic review	Spasticity—painful	Both	SR	10	N	We recommend TENS as a treatment for spasticity due to its low cost, ease of use, and absence of adverse reactions	+	?	
Synnot et al. [202]	Interventions for managing skeletal muscle spasticity following traumatic brain injury (Review)	Muscle spasticity—traumatic brain injury	Acute	CR	1	N	No statement of conclusion for TENS	?	?	
Mills et al. [200]	Transcutaneous Electrical Nerve Stimulation for Management of Limb Spasticity: A Systematic Review	Spasticity	Both	SR	14	N	There was level 1 and 2 evidence for TENS improving spasticity-related outcome measures…Better responses in outcome measures…when TENS was used in combination with active therapy (e.g., exercise and task-related training)	+	?	

Key: OSR = overview of systematic reviews; SR = systematic review; CR = Cochrane review; MR = mixed review; Y = yes; *n* = no; The column ‘Authors’ judgement’: + = evidence tending to favour TENS, − = evidence tending not to favour TENS, ? = evidence tending to be conflicting, inconclusive or insufficient to make a judgement; The column ‘Our Judgement’: + = Sufficient evidence to judge—TENS beneficial; − = Sufficient evidence to judge—TENS not beneficial; +/− = Sufficient evidence to judge—inconclusive; ? = Insufficient evidence to judge.

**Table 21:** Summary of reviews that include evaluation of TENS for post amputation pain. The column ‘Authors’ Conclusion’ contains statements taken from reports.

Ref.	Title	Condition	Acute/Chronic Pain	Review Type	Number of TENS Studies	MA	Authors’ Conclusion	Authors’ Judgement	Our Judgement	Comment
Johnson et al. [203]	Transcutaneous electrical nerve stimulation (TENS) for phantom pain and stump pain following amputation in adults (Review)	Amputation—phantom and stump pain	Chronic	CR	2	N	There were no RCTs to judge the effectiveness of TENS for the management of phantom pain and stump pain	?	?	There were two non RCTs
Hu et al. [209]	The effectiveness of acupuncture/TENS for phantom limb syndrome: A systematic review of controlled clinical trials	Amputation—phantom pain	Chronic	SR	3	N	There is some evidence for the use of acupuncture and TENS for the treatment of phantom limb pain, but insufficient high-quality evidence is available	?	?	
Halbert et al. [210]	Evidence for the Optimal Management of Acute and Chronic Phantom Pain: A Systematic Review	Amputation—phantom pain	Chronic	SR	3	N	No statement of conclusion for TENS	?	?	

Key: OSR = overview of systematic reviews; SR = systematic review; CR = Cochrane review; MR = mixed review; Y = yes; *n* = no; The column ‘Authors’ judgement’: + = evidence tending to favour TENS, − = evidence tending not to favour TENS, ? = evidence tending to be conflicting, inconclusive or insufficient to make a judgement; The column ‘Our Judgement’: + = Sufficient evidence to judge—TENS beneficial; − = Sufficient evidence to judge—TENS not beneficial; +/− = Sufficient evidence to judge—inconclusive; ? = Insufficient evidence to judge.

**Table 22:** Summary of reviews that include evaluation of TENS for various painful conditions where there are only two reviews. The column ‘Authors’ Conclusion’ contains statements taken from reports.

Ref.	Title	Condition	Acute/Chronic Pain	Review Type	Number of TENS Studies	MA	Authors’ Conclusion	Authors’ Judgement	Our Judgement	Comment
**Rheumatoid Arthritis**										
Brosseau et al. [205]	Transcutaneous electrical nerve stimulation (TENS) for the treatment of rheumatoid arthritis in the hand (Cochrane review)	Rheumatoid arthritis—hand	Chronic	CR	3	N	There are conflicting effects of TENS on pain outcomes in patients with RA. AL-TENS is beneficial for reducing pain intensity and improving muscle power scores over placebo while, conversely, C-TENS resulted in no clinical benefit on pain intensity compared with placebo. However, C-TENS resulted in a clinical benefit on patient assessment of change in disease over AL-TENS	?	?	Journal version also available in Brosseau et al. [62]
Ottawa Panel [204]	Ottawa Panel Evidence-Based Clinical Practice Guidelines for Electrotherapy and Thermotherapy Interventions in the Management of Rheumatoid Arthritis in Adults	Rheumatoid Arthritis	Chronic	SR	3	N	The Ottawa Panel recommends the use of…TENS…for the management of rheumatoid arthritis. Low-frequency TENS applied to the hand and wrist versus no stimulation, level I (RCT): grade A for pain at 3 weeks (clinically important benefit). High-frequency TENS applied to the hand and wrist versus placebo, level I (RCT): grade C for pain and joint tenderness, same day (no benefit).	+	?	
**Headache or migraine**										
Tao et al. [207]	Effectiveness of transcutaneous electrical nerve stimulation for the treatment of migraine: a meta-analysis of randomized controlled trials	Migraine	Chronic	SR	4	Y	This meta-analysis suggests that TENS may serve as an effective and well-tolerated alternative for migraineurs. However, low quality of evidence prevents us from reaching definitive conclusions RCTs	+	?	Some interventions not standard TENS
Bronfort et al. [211]	Non-invasive physical treatments for chronic/recurrent headache	Headache	Chronic	CR	2	N	There is preliminary evidence that a combination of TENS and electrical neurotransmitter modulation is inferior to biofeedback and superior to relaxation for reduction of headache pain…evidence from one trial). There is limited evidence that a regimen of auto-massage, TENS, and stretching is superior to acupuncture for pain relief 4 to 9 weeks post-treatment. (evidence from one trial).	?	?	
**Carpal tunnel syndrome**										
Huisstede et al. [212]	Carpal Tunnel Syndrome: Effectiveness of Physical Therapy and Electrophysical Modalities. An Updated Systematic Review of Randomized Controlled Trials	Carpal Tunnel	Chronic	SR	2	N	…there is moderate evidence that interferential current therapy is more effective than a night splint or TENS in the short term	−	?	
Peters et al. [213]	Rehabilitation following carpal tunnel release	Carpal tunnel	Unclear	CR	0	N	No statement of conclusion for TENS	?	?	
**Fracture Pain**										
Perillo et al. [214]	Pre-hospital femoral neck fracture management: A review of the literature	Bone fracture	Acute	SR	1	N	No statement of conclusion for TENS	?	?	One study found TENS effective in treating pain in the prehospital environment
Abou-Setta et al. [90]	Comparative Effectiveness of Pain Management Interventions for Hip Fracture: A Systematic Review	Hip fracture	Acute	SR	2	Y	…based on limited evidence, [TENS] seem to be safe and may result in clinically meaningful reductions in pain	?	?	

Key: OSR = overview of systematic reviews; SR = systematic review; CR = Cochrane review; MR = mixed review; Y = yes; *n* = no; The column ‘Authors’ judgement’: + = evidence tending to favour TENS, − = evidence tending not to favour TENS, ? = evidence tending to be conflicting, inconclusive or insufficient to make a judgement; The column ‘Our Judgement’: + = Sufficient evidence to judge—TENS beneficial; − = Sufficient evidence to judge—TENS not beneficial; +/− = Sufficient evidence to judge—inconclusive; ? = Insufficient evidence to judge.

### 3.6. Outcomes for Adverse Events

Generally, systematic reviews did not pre-specify a protocol to evaluate adverse events. Those that did (e.g., all Cochrane reviews) found that most studies captured adverse events spontaneously (ad hoc) and did not have pre-specified protocols for gathering nor analysing adverse events and/or the safety of using TENS. There were no serious adverse events reported in the included reviews, and adverse events that were reported were infrequent and of minimal severity, such as mild skin irritation or discomfort from electrical currents. We judged that evidence was inconclusive for harm, but tending toward TENS being safe, with negligible adverse events.

### 3.7. Synopsis of Characteristics and Outcomes

A synopsis of the analyses of characteristics and outcomes of included reviews is summarised in Table 24. In summary, the methodological quality of many reviews is good, especially those conducted by the Cochrane Collaboration, but unfortunately these reviews reveal a paucity of studies, and/or studies that have insufficient samples sizes, high risk of bias, and/or are poorly communicated. The majority of meta-analyses did not meet our threshold for sufficient pooled data points to have confidence in the precision of effect size estimates. There was much variability in meta-analytical procedures for pain intensity including; the type of estimate reported e.g., SMD, MD, RR; the timepoint used e.g., during or post TENS; whether the estimate was absolute or relative to baseline; and whether fixed or random effects models were employed. There was evidence of violation of unit of analyses principles including estimates that had double counted study data. There were instances of a paucity of systematic reviews for common painful conditions e.g., rheumatoid arthritis, and instances of persistent publication of systematic reviews despite a paucity of primary studies e.g., non-specific low back pain.

**Table 23:** Summary of reviews that include evaluation of TENS for various painful conditions where there is only one review. The column ‘Authors’ Conclusion’ contains statements taken from reports.

Ref.	Title	Condition	Acute/Chronic Pain	Review Type	Number of TENS Studies	MA	Authors’ Conclusion	Authors’ Judgement	Our Judgement	Comment
Deussen et al. [215]	Relief of pain due to uterine cramping/involution after birth	Uterine cramping/involution after birth	Acute	CR	3	N	Very low-certainty evidence means we are uncertain if TENS is better than no TENS for adequate pain relief as reported by the women	?	?	Update of Deussen et al. [216]
Pal et al. [217]	Transcutaneous electrical nerve stimulation (TENS) for pain management in sickle cell disease (Review)	Sickle cell pain	Acute	CR	1	N	Since we have only included one small and very low-quality trial, with a high risk of bias across several domains, we are unable to conclude whether TENS is harmful or beneficial for managing pain in people with sickle cell disease	?	?	
De Andres et al. [218]	Vulvodynia—An Evidence-Based Literature Review and Proposed Treatment Algorithm	Vulvodynia	Chronic	SR	3	N	No statement of conclusion for TENS	?	?	
Liao et al. [219]	Efficacy of Non-invasive Stellate Ganglion Blockade Performed Using Physical Agent Modalities in Patients with Sympathetic Hyperactivity-Associated Disorders: A Systematic Review and Meta-Analysis.	Sympathetic Hyperactivity-Associated Disorders	Unclear	SR	3	N	Non-invasive Stellate Ganglion Blockade performed using PAMs [including TENS] effectively relieves pain of various etiologies, making it a valuable addition to the contemporary pain management armamentarium	?	?	
Oor et al. [220]	A systematic review of the treatment for abdominal cutaneous nerve entrapment syndrome	Nerve entrapment syndrome—abdominal cutaneous	Chronic	SR	0	N	No statement of conclusion for TENS	?	?	
Smart et al. [208]	Physiotherapy for pain and disability in adults with complex regional pain syndrome (CRPS) types I and II.	Complex regional pain syndrome	Both	CR	6	N	Evidence of the effectiveness of multimodal physiotherapy, electrotherapy and manual lymphatic drainage for treating people with CRPS types I and II is generally absent or unclear	+	?	
Mansilla et al. [221]	Efficacy of transcutaneous electrical stimulation in trigeminal neuralgia. Eficacia de la estimulación eléctrica transcutánea en la neuralgia del trigémino. Rehabilitacion, 50, 81–86.	Trigeminal neuralgia	Chronic	SR	2	N	On the basis of published studies, TENS contributes positively to pain relief and functional improvement in patients affected by trigeminal pain. A larger number of studies are needed to…recommend its use	+	?	In Portuguese
Kovacs et al. [222]	Surgery versus conservative treatment for symptomatic lumbar spinal stenosis: a systematic review of randomized controlled trials.	Spinal stenosis	Chronic	SR	1	N	One RCT on TENS as a comparator and uses TENS but is in combination with ultrasound and exercise. Not possible to isolate TENS	?	?	
McKneely et al. [223]	A systematic review of the effectiveness of physical therapy interventions for temporomandibular disorders.	temporomandibular disorders	Unclear	SR	2	N	…further research is warranted before dismissing any effect of TENS	?	?	

Key: OSR = overview of systematic reviews; SR = systematic review; CR = Cochrane review; MR = mixed review; Y = yes; *n* = no; The column ‘Authors’ judgement’: + = evidence tending to favour TENS, − = evidence tending not to favour TENS, ? = evidence tending to be conflicting, inconclusive or insufficient to make a judgement; The column ‘Our Judgement’: + = Sufficient evidence to judge—TENS beneficial; − = Sufficient evidence to judge—TENS not beneficial; +/− = Sufficient evidence to judge—inconclusive; ? = Insufficient evidence to judge.

## 4. Discussion

Pain mechanisms are complex, resulting in uncertainty in finite diagnoses. Pain acts to protect the integrity of tissue and does not act as a marker of tissue damage, i.e., hurt does not always mean harm. In medicine, great effort is given to identifying input to the brain in the form of ‘pain generators’ such as nociception (nociceptive pain) or neuropathy (neuropathic pain) and associated sensitisation and bioplasticity (nociplastic pain). However, contemporary neuroscience acknowledges that pain is an output of the brain based on perceptual inference and influenced by a wide variety of biopsychosocial factors. In other words, ‘everything matters when it comes to pain’, including feelings, activities, stress, sleep, anxiety, unemployment, social situation, and self-identity to name but a few. Pain medicine has been slow to deliver a biopsychosocial model at the point of care, and there is continued uncertainty whether to select treatment according to symptoms or medical diagnosis.

It is acknowledged that physical activity and psychological interventions delivered within a self-management strategy is optimal for pain management. The goal is to improve activities of daily living and quality of life through pain education, lifestyle adjustment and healthy living. Surgery and medication are often not the answer because they can be harmful and efficacy in the long-term is doubtful. Neuromodulation techniques such as TENS are used within this framework to alleviate the sensations of pain, muscle tension and spasm. This provides a variety of indirect benefits including enhanced function, improved psychological well-being, better sleep, and medication reduction. TENS is widely accepted by patients because it is in-expensive, it can be self-administered, and it has few adverse events and minimal toxicity. TENS has the potential to be used for any type of acute or chronic pain, such as post-operative pain, labour pain, neuropathic pain, and non-specific musculoskeletal pain. The ease of use of TENS makes it ideal for a variety of care settings including community care, primary care, secondary care (in-patient and outpatient hospital settings), tertiary care (e.g., hospice settings), workplace settings (for occupational-related pain) and sport-settings (for sports-related pain).

### 4.1. Main Findings

To our knowledge, this is the first comprehensive appraisal of all published reviews that have used a systematic approach to find primary studies assessing the clinical efficacy of TENS for any type of acute and chronic pain in adults. Our appraisal is the largest to date, and included 169 reviews, of which 154 were systematic reviews and 37 were published by the Cochrane Collaboration. Our appraisal reveals an overwhelming quantity of redundant primary and secondary research that spans over three decades, resulting in stagnation of evidence and uncertainty about efficacy. Nevertheless, we believe that our appraisal provides ‘suggestive evidence’ that pain intensity is lower during or immediately after TENS compared with control interventions for a variety of conditions. Scrutiny of tallies and effect size estimates did not suggest that there were any major differences between pain duration (i.e., acute versus chronic) nor types of pain (e.g., musculoskeletal, post-operative, labour, neuropathic etc.). Our appraisal exposes shortcomings in research to date, including inconsistencies in methodologies, analyses and findings. We will consider the limitations and strengths of our appraisal before discussing the implications of the findings and issues arising for clinical practice and future research.

### 4.2. Limitations of Our Appraisal

Potential limitations of our appraisal process include:

- Absence of an analysis of adverse events
- Absence of a quality assessment of included reviews
- Absence of an analysis of TENS technique, dose or timing of outcomes on judgements

We will discuss each of these in turn.

#### 4.2.1. Absence of an Analysis of Adverse Events

We were unable to appraise adverse events because of a paucity of information in reviews. Few reviews had pre-specified protocols to analyse adverse event data. When information was available it was usually a brief narrative of adverse events collected spontaneously within trials. The absence of descriptions of adverse events within trial reports and reviews implies absence of adverse events of any consequence, although we cannot be entirely certain of this

#### 4.2.2. Absence of a Quality Assessment of Included Reviews

We pre-specified that we would not formally assess the quality of systematic reviews because this has already been undertaken in overviews of systematic reviews. Gibson et al. [6], in their overview of eight Cochrane reviews, judged systematic review methodology to be good, but the quality of RCTs in reviews were very low. In other words, systematic review findings are only as good as the quality of studies assessed. We believe that adequacy of sample size for pooled data is the critical factor when judging meta-analyses findings for TENS, and our appraisal accounted for this when judging review evidence. Thus, we do not believe that undertaking a formal quality assessment of review would alter the outcome of our appraisal or add further insight into the nature of the evidence at our disposal.

#### 4.2.3. Absence of an Analysis of TENS Technique, Dose or Timing of Outcomes on Judgements

It was not our intention to undertake a granular analysis of TENS technique, dose or timing of outcomes on outcome. In 2021, we published a comprehensive review of factors influencing the effects of TENS on pain that found no robust evidence of a relationship between specific electrical characteristics of TENS and clinically meaningful outcomes in patients with different types of pain [235]. Evidence suggested that a strong non-painful TENS sensation at, or close to the site of pain, was the active ingredient for TENS and patients adjust the qualities of this sensation according to personal preference, which may vary within and between treatments. We argued that it may be more appropriate to view TENS as a ‘blunt tool’ to generate a pleasant sensory experience to ease pain rather than a ‘precise tool’ enabling selection of specific electrical characteristics for specific types of pain. In other words, TENS should be considered as providing ‘sensory soothing’, akin to rubbing, warming and cooling the skin. Thus, we do not believe TENS technique, dose or precise timing of outcomes have influenced the gross-level findings of our appraisal.

### 4.3. Challenges Encountered Conducting Our Descriptive Analysis

We encountered operational challenges during screening of reports and whilst extracting, categorising and tallying data. We will discuss each of these in turn.

#### 4.3.1. Screening Reports for Inclusion

Screening reports proved challenging. There were instances of multiple citations of reviews with subtle differences in publication dates and/or authorship teams; and multiple reports of the same systematic review and meta-analysis, including some Cochrane reviews published as shorter or extended reports in journals. We gave careful consideration to our approach to screen reports for inclusion in our appraisal to reduce the incidence of ‘double counting’, as described in the Section 2 and Supplementary Materials. Some Cochrane reviews were cited as ‘Withdrawn’ and without an accompanying explanation (e.g., Nnoaham and Kumbang [236]). Originally, Cochrane reviews could be withdrawn for a variety of reasons, including retraction of the review due to errors or a change in focus of updated reviews, e.g., the review being split into multiple reviews. Cochrane have recently updated their policy so that reviews are only withdrawn in ‘exceptional circumstances’ when concern arises about the conduct or reporting, such as serious error(s) in the review process, scientific misconduct, or a breach of Cochrane’s conflict of interest policy. We considered each withdrawn review on a case-by-case basis. For example, we included a review by Nnoaham and Kumbang that was withdrawn in 2014 [236] as it met our eligibility criteria, had not been subsequently updated, and had not been withdrawn for reasons associated with reporting errors or conduct.

#### 4.3.2. Extracting, Categorising and Tallying Data

Selecting data to extract from reviews and meta-analyses was challenging due to unclear and inconsistent reporting of trial arm sample sizes, values for overall effect size estimates, and the number of studies and participants in reviews versus meta-analyses. Some reviews and meta-analyses had a mixture of RCTs and non-RCTs. Many TENS interventions were administered in combination with other treatments including rescue medication, leading to ‘contamination’ of outcome data. Nevertheless, we do not believe that this impacted on the accuracy of our analysis of characteristics, outcomes or conclusion.

We used two investigators to independently extract, count and spot check the accuracy of data in our spreadsheet. Nevertheless, the size and complexity of the data extraction, coding and counting is likely to have resulted in the introduction of an occasional random error. We do not believe that this will have any significant impact on the accuracy of tallies in our descriptive analyses. The intention of our analysis was to offer insights to the characteristics of the systematic review literature as a whole rather than minutia.

We experienced challenges categorising types of pain, especially in relation to medical diagnoses. For example, Desmeules et al. [170] published a systematic review that evaluated rotator cuff tendinopathy which we categorised as tendinopathy, although it could equally have been included within our analysis of shoulder pain or musculoskeletal pain. We are confident that potential violations of categorisation were negligible. Nevertheless, the appropriateness of classifying painful conditions at a granular level, especially when evaluating treatments for symptomatic relief of pain via neuromodulation, is not without complication because medical conditions may present with a variety of painful symptoms resulting from multiple causes. For example, Sawant et al. [79] and Jawahar et al. [193] evaluated TENS for central neuropathic pain in individuals with multiple sclerosis, whereas Amatya et al. [192] evaluated TENS for chronic pain in individuals with multiple sclerosis without reference to central neuropathic pain and all three reviews included an RCT by Warke et al. [37] that evaluated chronic low-back pain in a multiple sclerosis population. It is a matter for debate whether chronic low back pain is a direct or indirect result of multiple sclerosis; was centrally or peripherally driven; was of neuropathic origin; and/or presented with symptoms that are considered to be characteristic in quality to neuropathic pain. We are of the view the primary mechanism of pain relief during TENS is via neuromodulation and therefore outcome would not depend to any great extent on specific pain mechanisms.

Readers may disagree with our categorisation and organisation of types of pain for some of our included reviews. Our approach was pragmatic, taking into consideration the number of available reviews and types of pain frequently described by clinicians. We considered categorising conditions according to the ICD-11 system for chronic pain, yet this can be problematic [237,238]. For example, non-specific chronic low back pain is considered by most clinicians as a musculoskeletal condition and managed in clinical settings associated with secondary musculoskeletal pain such as osteoarthritis, yet it is categorised in ICD-11 as chronic primary pain rather than secondary musculoskeletal pain. Some chronic primary pains are not similar in character to non-specific chronic low back pain e.g., complex regional pain syndrome. We used a robust approach to prevent ‘double counting’ so we do not believe that the way we organised reporting of type of pain affected the outcome of our descriptive analyses.

Finally, our approach to assigning authors’ conclusions was subjective and biased toward a dichotomous judgement of benefit or no benefit, if at all possible. We often encountered illogical statements such as ‘…TENS may be beneficial, but evidence was inconclusive…’ which could just as easily have been reverse framed as ‘…TENS may be not beneficial, but evidence is inconclusive…’. Ideally, the statement should be framed ‘It is not possible to determine efficacy because evidence is inconclusive’. As it was not our intention to tally definitive outcome of efficacy but rather to explore the structure, tone and direction of statements as reflected by the authors’ sense of efficacy, we assigned ‘…TENS may be beneficial, but evidence was inconclusive…’ as ‘Benefit’; and ‘…TENS is not beneficial, but evidence is inconclusive…’ as ‘Not Benefit’. Despite this, we still recorded a high quantity of ‘Inconclusive’ outcomes. Importantly, our analysis reveals that many conclusions were framed using imprecise, contradictory or illogical language, with a bias towards evidence of possible benefits rather than no benefit.

Overall, the operational challenges faced whilst undertaking this appraisal provide useful insights into factors that influence outcomes of reviews. In our opinion, the limitations discussed above do not impact to any significant degree on the gross findings from our appraisal. We emphasise that the intention of our appraisal was to overview the characteristics and outcomes of systematic reviews evaluating clinical efficacy but not to determine efficacy per se.

### 4.4. Strengths of Our Appraisal

The strength of our appraisal is that it is the first to adopt a systematic, comprehensive and analytical approach to evaluate the full extent of systematic review literature on TENS. Thus, our appraisal can be used as a ‘one-stop resource’ for patients, clinicians, funders and policy makers. Our approach is transparent and our operational aide memoires (Supplementary Materials) enable replication of our methods and analyses. We pre-specified gold standard criteria for sufficient data on which to base judgements about overall estimates of effect size of meta-analyses. However, our intention was to describe and appraise the literature rather than undertake a formal evaluation of primary source data. Thus, we pre-specified that we would not attempt to extract primary source data for meta-analysis because we are already conducting a systematic review and meta-analysis that will do so [15].

### 4.5. Comments on the Body of Evidence

#### 4.5.1. Unnecessary/Redundant Systematic Reviews

Our appraisal revealed instances of multiple reports of the same systematic review and revealed a proliferation of unnecessary reviews in the preceding three decades. A variety of factors are likely responsible including pressures for academics and clinicians to publish research for career progression, the low running costs of undertaking ‘desk based’ reviews, and an ever-increasing array of journals in which to publish. Our appraisal also revealed a high incidence of unnecessary systematic reviews, with more systematic reviews than RCTs for a number of painful conditions (e.g., non-specific chronic low back pain). Some reviews included forest plots of *n* = 1, which could give a false impression of meta-analysis to the gullible reader. The proliferation of unnecessary, misleading, conflicting and inconclusive systematic reviews and meta-analyses has been recognised as a serious problem in medicine and health care, often confusing rather than clarifying benefits and harms of treatments [239]. This is particularly apparent in the body of literature on TENS, and it has resulted in conflicting clinical guidelines and much confusion for practitioners and patients.

#### 4.5.2. Variability in the Reporting and Execution of Reviews

There was variability in the way that review methodology and findings were communicated. Cochrane reviews provided the greatest depth, detail and consistency of reporting. Reports of reviews undertaken by some learned societies lacked sufficient detail to replicate methodology, reducing confidence in operationalization of review methodology process and subsequent conclusions (e.g., Dubinsky et al. [115], rebuttal by Johnson and Walsh [116]). That said, even well written, systematic reviews do not necessarily assure a well conducted evaluation, as most reports did not provide specific details about operational aspects of conducting the review. Most reports suggested that investigators followed standard systematic review methodology, although we revealed inconsistency in included studies, risk of bias judgements, clinical outcomes, measurement timepoints and the nature of the effect size estimated between reviews of identical pain conditions. We suspect this is due to operational variations when conducting the review.

Specific operational aspects of undertaking reviews remain absent from review reports due to constraints on editorial space. Few reports had Supplementary Materials, and those that did tended to provide details of numerical data supporting analysis and/or details about the characteristics of studies. Few, if any, provided operational aide memoires to facilitate critique and replication of methodological steps taken during review. We recommend that reviewers should develop operational aide memoires specific to their reviews (e.g., screening and risk of bias judgments) and based on ‘gold standard’ guidance (e.g., from the Cochrane Handbook). This will facilitate consistency of decision making within review teams, replication of decision-making processes by other review teams, and open debate about operational procedures. These aide memoires can be published as Supplementary Materials as we have done for this appraisal (Supplementary Materials).

#### 4.5.3. Exaggeration of Process and Findings

There was evidence of overstatement of all aspects of the review process in reports. For example, Zhu et al. [75] stated in the Abstract that there were six RCTs involving 529 patients in their meta-analysis, suggesting a modest amount of pooled data. However, in reality there were three separate meta-analyses each consisting of only two RCTs, and none had a total sample of pooled data greater than 210 participants (i.e., VAS TENS vs. control, two RCTs, *n* = 51 vs. 51; VAS at 3 weeks TENS vs. control, 2 RCTs, *n* = 103 vs. 102; post-operative morphine consumption TENS vs. Control, 2 RCTs, *n* = 46 vs. 43). For clarity, we recommend that reviewers should include n values for the trial arms of the largest pooled data set in report Abstracts and summary statements of findings.

Also, as described previously, we found that many reviewers tended to frame inconclusive findings in a positive tone, e.g., ‘might be effective, despite a paucity of data’ rather than a negative tone, e.g., ‘might be ineffective, despite a paucity of data’, or non-committal, e.g., ‘insufficient data to judge’ or ‘sufficient data that is conflicting’. A minority of conclusions were framed inconclusive findings in a negative tone, e.g., ‘no evidence of benefit’. We recommend that authors pay more attention to the precision of language when constructing concluding statements, as this can have a major impact on the take-home message. No evidence of benefit is not the same as evidence of no benefit.

#### 4.5.4. Inadequacy of RCT Design

All reports of systematic reviews stated that the quality of RCTs was low and that larger better designed RCTs were needed in the future. Our appraisal found RCTs to be simplistic, parallel group or cross-over in design. Reviewers persistently criticized inadequate sample sizes, lack of ‘blinding’ of comparator interventions, and heterogeneous TENS technique, dose, and regimen. Previously, we have commented on long-standing failure to address methodological deficiencies in TENS trials and have published attributes, criteria and operational solutions for undertaking an ideal TENS RCT [240]. We have recommended the use of enriched enrolment randomised withdrawal trials to evaluate the efficacy of TENS in real world situations [15,235].

### 4.6. Stagnation of Knowledge over Three Decades

Our appraisal provides interesting insights into the evolution of systematic review evidence over three decades. In the early 1970s Long et al., concluded that “*…the initial success that we have gained to date suggests that cutaneous electrical stimulation will be a significant advance in our ability to treat chronic pain*” ([2], p. 267). The authors of the earliest systematic reviews published in the 1990s concluded “*The use of TENS in chronic pain may well be justified but it has not been seen*” ([3], p. 49)…“*There is a requirement for a randomised trial to address the issue.…Without it, a potentially valuable intervention may be underused, or a useless intervention may continue in use*” ([3], p. 49). “*There is insufficient evidence to draw any conclusions about the effectiveness of transcutaneous electrical nerve stimulation (TENS) for the treatment of chronic pain in adults*…*Large multi-centre randomised controlled trials of TENS in chronic pain are urgently needed.*” Carroll et al., 2001 ([97], p. 2). In 2020, Gibson et al. [6] concluded “*We were therefore unable to conclude with any confidence that, in people with chronic pain, TENS is harmful, or beneficial for pain control, disability, health-related quality of life, use of pain-relieving medicines, or global impression of change*” ([6], p. 2). “…*there is an urgent need to undertake large RCTs to examine its effectiveness*”([6], p. 9).

The stagnation of evidence is the result of a lack of appetite to change the approach to evaluating TENS efficacy. Our appraisal is unique because of the extensive scope of the review (i.e., all types of pain) and the comprehensive nature of the search of literature and subsequent criterion-based judgements of efficacy based on systematic reviews with sufficient pooled data for meta-analysis.

### 4.7. The Efficacy of TENS

Our tally of authors’ conclusions found that the majority of systematic reviews were inconclusive. Only a small proportion of reviews included a meta-analysis of pooled data and most did not pool sufficient data to be confident of claims of benefit. These findings are consistent with the most recent Cochrane reviews on acute [83] and chronic pain [6]. Both were inconclusive. The Cochrane review on acute pain was limited in scope, only assessing TENS as a standalone treatment resulting in the exclusion of a large quantity of studies on post-operative pain [83]. The most recent Cochrane review evaluating TENS for chronic pain included 8 Cochrane reviews and a descriptive analysis of 51 RCTs, without meta-analysis because they judged there to be considerable heterogeneity associated with clinical conditions, treatment protocols and study methodology including inadequate sample sizes [6]. Thus, the vast majority of systematic reviews spanning half a century have added little to knowledge and if anything, have fuel long-standing uncertainty about TENS efficacy.

Our appraisal is the first to graphically summarise all available effect size estimates of pain intensity (continuous) data and suggests that pain intensity is lower during TENS compared with controls, as most confidence intervals of overall effect size did not bisect the line of no difference. Most meta-analyses failed to reach our threshold for sufficient data to have confidence in the precision of the estimate (i.e., pooled analysis of ≥500 events). Thus, we were unable to undertake any formal summary analysis of the effect sizes of all meta-analyses because inconsistency in calculations to estimate effect size in reviews hindered any meaningful comparison of equivalent outcomes (e.g., see Table 2 and Table 3, Figure 11). Nevertheless, there were two meta-analyses with sufficient data and both were in favour of TENS; for chronic musculoskeletal pain [10] and for labour pain [26]. We also identified one meta-analysis with sufficient data to suggest that there was lower post-operative analgesic consumption during TENS compared with control [11].

In summary, we believe that our appraisal provides persuasive rather than compelling evidence that pain intensity is lower during or immediately after TENS compared with control interventions for acute and chronic pain. As a consequence, we believe that practitioners and policy makers should offer TENS as a treatment option for symptomatic relief of pain.

### 4.8. Implications for Clinical Practice and Future Research

TENS is a complex intervention and users need to personalise their treatment strategy according to their personal needs [241]. This involves learning how to select beneficial electrode positions and electrical characteristics (pulse amplitude, frequency, and pattern) based on their pain at that moment. Thus, users need to learn how to optimise benefits and minimize problems through trial and error and in line with good practice guidelines [1,242,243].

Recently, the NICE guidelines for the assessment of chronic pain and management of chronic primary pain recommended that TENS should not be offered [157]. The NICE excluded an evaluation of TENS for non-specific low back pain because separate guidance had already been published for the management of non-specific low back pain in 2016 [244]. This meant that data from studies evaluating TENS for non-specific low back pain were absent from the estimation of the overall effect size, which was calculated from only two studies, both on fibromyalgia. Although inclusion of the non-specific chronic low back pain studies in the meta-analysis would be unlikely to have changed the precision or confidence in effect size estimate, it does highlight the difficulty of categorising pain according to medical condition.

Our appraisal revealed 169 reviews that focused on pain associated with a variety of specific medical conditions with the majority of the common pain diagnoses covered. There was no evidence in our appraisal that the efficacy of TENS varied according to medical diagnoses, suggesting that TENS effects are generic, irrespective of the type of pain. We found no strong evidence that efficacy depended on specific electrical characteristics of TENS. Nevertheless, this would need to be confirmed in a meta-analysis that explored clinical heterogeneity (e.g., various types of pain and treatment protocols), and also statistical (i.e., I^2^) and methodological (e.g., high risk of bias) heterogeneity, using sensitivity and/or subgroup analyses. We have published a protocol for such a meta-analysis that will use a GRADE approach to account for the influence of study limitations (risk of bias), publication bias and inconsistency, indirectness and imprecision on confidence and certainty in the effect size estimate [15].

We also recommend that more ecologically valid RCTs are needed to evaluate TENS in ‘real-life’ settings such as self-administering TENS at home for chronic pain, capturing what patients choose to use rather than what practitioners or investigators prescribe. Participants should be actively engaged in the ‘design’ of their treatment schedule and choice of outcome measures meaningful to their needs. An enriched enrolment randomised withdrawal trial design meets these requirements and we have described in detail the characteristics of such a trial [235].

## 5. Conclusions

Our intention is that this appraisal of all available systematic reviews and meta-analyses of RCTs evaluating the effect of TENS on pain intensity will serve as a useful reference source for practitioners, researchers and commissioners. Our appraisal charts the research evidence underpinning long-standing uncertainty about clinical efficacy. Our appraisal reveals examples of meta-analyses with ‘sufficient data’ demonstrating benefit. There were no examples of meta-analyses with ‘sufficient data’ demonstrating no benefit. Therefore, we recommend that TENS should be considered as a treatment option.

However, when taken as a whole, the systematic review evidence is not compelling because of a considerable quantity of systematic reviews with ‘insufficient data’ contributing to meaningless and confusing literature that cloud the issue. We recommend a meta-analysis that pools all available RCT data, irrespective of type of pain, to estimate effect size for pain intensity. Going forward, more ecologically valid clinical trials of TENS are required using enriched enrolment with randomised withdrawal trial designs.

Ultimately, we hope that our appraisal catalyses a reflection on this situation and the need for a stepwise change in the way we evaluate treatments like TENS, where confident judgements about efficacy seem elusive.

## Figures and Tables

**Table 1:** Inconsistency in studies included in systematic reviews on non-specific back pain published within the previous 10 years. Studies where data was extracted to estimate effect size estimates for pain intensity (continuous data) in previous systematic reviews are identified.

Systematic Review.	Number of Studies Included in Review	Facci et al.,(2011) [28]	Ratajczak et al.,(2011) [29]	Sahin et al.,(2011) [30]	Alizadeh et al.,(2009) [31]	Itoh et al.,(2009) [32]	Barker et al.,(2008) [33]	Kofotolis et al.,(2008) [34]	Thompson et al.,(2008) [35]	Shimoji et al.,(2007) [36]	Warke et al.,(2006) [37]	Jarzem et al.,(2005) [38]	Jarzem et al.,(2005) [39]	Topuz et al.,(2004) [27]	Yokoyama et al.,(2004) [40]	Hsieh and Lee (2002) [41]	Tsukayama et al.,(2002) [42]	Cheing et al.,(1999) [43]	Grant et al.,(1999) [44]	Ghoname et al.,(1999) [45]	Moore and Shurman (1997) [46]	Marchand et al.,(1993) [47]	Gemignani et al.,(1991) [48]	Deyo et al.,(1990) [49]	Lehmann et al.,(1986) [50]
Resende et al. [51]	9	I		I	I	I DE		I DE						I DE				I DE				I DE		I DE	
Wu et al. [14]	12					I DE		I DE	I DE	I DE	I DE	I		I DE	I DE	I DE	I DE			I DE	I DE				
Jauregui et al. [52]	13	I DE	I DE			I DE	I DE						I	I DE	I DE			I DE			I DE	I DE	I DE	I DE	I DE
Van Middelkoop et al. [53]	7	N/A	N/A	N/A								I DE	I DE	I DE	I DE				I DE	I DE				I DE	

Key: I = included in systematic review, DE = data extracted, N/A = study not published after the publication of the review. Green represents study included in the review and pain intensity (continuous) data extracted, Orange represents study included in the review, but pain intensity (continuous) data was not extracted, Red represents study was not included in the review and pain intensity (continuous) data was not extracted. Observations: Itoh et al., (2009) [32] used interferential current therapy not TENS; Thompson et al., (2008) [35] used Transcutaneous Spinal Electroanalgesia (TSE) not TENS; Shimoji et al., (2007) [36] used bidirectional modulated sine waves rather than TENS, van Middelkoop et al. [53] stated that six TENS studies were included although they also included an additional study that examined TENS and categorised under exercise interventions rather than TENS intervetions.

**Table 5:** Summary of reviews that include evaluation of TENS for musculoskeletal pain. The column ‘Authors’ Conclusion’ contains statements taken from reports.

Ref.	Title	Condition	Acute/Chronic Pain	Review Type	Number of TENS Studies	MA	Authors’ Conclusion	Authors’ Judgement	Our Judgement	Comment
**Various chronic musculoskeletal pain** **(9 reviews)**										
Ely et al. [101]	Transcutaneous electrical acupoint stimulation for people with chronic musculoskeletal pain: an exploratory review	Musculoskeletal pain—chronic	Chronic	SR	20	N	People with chronic musculoskeletal pain may achieve pain relief using transcutaneous electric acupoint stimulation but the existing evidence is limited, and high-quality clinical evidence is required to establish efficacy	?	?	We suspect that this is the full report of Ely et al. [102]
Nunes et al. [103]	Effectiveness of physical and rehabilitation techniques in reducing pain in chronic trapezius myalgia: A systematic review and meta-analysis	Myalgia—chronic trapezius	Chronic	SR	1	N	There was very poor evidence that TENS therapy and manual therapy are effective in treating chronic trapezius myalgia	?	?	
Almeida et al. [104]	Conservative interventions for treating exercise-related musculotendinous, ligamentous and osseous groin pain	Exercise-related musculotendinous, ligamentous and osseous groin pain	UC	CR	2	N	The available evidence from the randomized trials is insufficient to advise on any specific conservative modality for treating exercise related groin pain.	?	?	The authors could not isolate effect of TENS because the included studies delivered TENS as part of multimodal physiotherapy treatment
Bellini et al. [105]	Physical therapy applied to pathologies of rehabilitative interest	Musculoskeletal pain	Chronic	SR	5	N	TENS is recommended for the treatment of tibio-femoral osteoarthritis, its effectiveness is questionable for carpal tunnel syndrome and not recommended for the treatment of chronic low-back pain	+	?	
Vernon et al. [106]	Chiropractic management of myofascial trigger points and myofascial pain syndrome: a systematic review of the literature	Myofascial pain	Both	SR	6	N	There is moderately strong evidence that TENS may be effective in providing immediate relief at trigger point. The evidence level is B.	+	?	Evaluated commonly used treatments
Johnson et al. [10]	Efficacy of electrical nerve stimulation for chronic musculoskeletal pain: A meta-analysis of randomized controlled trials	Musculoskeletal pain—chronic	Chronic	SR	29	Y	These results indicate that electrical nerve stimulation is an effective treatment modality for chronic musculoskeletal pain and that previous, equivocal results may have been due to underpowered studies.	+	+	There were 32 RCTS of which 29 were on TENS and the rest on peripheral nerve stimuation
Rickards et al. [107]	Myofascial trigger point pain	Myofascial	UC	SR	7	N	TENS appears to have an immediate effect in decreasing pain intensity in myofascial trigger point pain of the neck and upper back. However, there are insufficient data to provide the evidence of effectiveness for TENS beyond immediately after treatment.	+	?	
O’Connor et al. [108]	The effectiveness of physiotherapeutic interventions in the management of delayed onset muscle soreness: A systematic review	Muscle soreness—post exercise	Acute	SR	3	N	The evidence did not support the use of static stretching, cryotherapy, acupuncture, pulsed ultrasound, TENS, interferential therapy, and microcurrent electrical stimulation	?	?	The authors stated that the evidence was conflicting
Philadelphia Panel [109]	Philadelphia Panel Evidence-Based Clinical Practice Guidelines on Selected Rehabilitation Interventions for Shoulder Pain	Shoulder pain—non-specific	Both	SR	1	N	…a lack of evidence regarding efficacy	?	?	
**Non-specific back pain 21 reviews**										
Nascimento et al. [110]	Effectiveness of interventions for non-specific low back pain in older adults. A systematic review and meta-analysis	Back pain—chronic low	Chronic	SR	1	N	No statement of conclusion for TENS	?	?	The authors commented that a previous study showed a higher effectiveness of percutaneous electrical nerve stimulation (PENS) to decrease pain compared to TENS at short-term follow-up.
Resende et al. [51]	Meta-analysis of transcutaneous electrical nerve stimulation for relief of spinal pain	Back and/or neck pain—chronic	Chronic	SR	9	Y	…inconclusive evidence of TENS benefits in low back pain patients because the quality of the studies was low, and adequate parameters and timing of assessment were not uniformly used or reported.	?	?	Nine RCTs with seven data sets included for meta-analysis (655 participants)
Wu et al. [14]	Literature Review and Meta-Analysis of Transcutaneous Electrical Nerve Stimulation in Treating Chronic Back Pain	Back pain—Chronic	Chronic	SR	12	Y	These results suggest that TENS does not improve symptoms of lower back pain but may offer short-term improvement of functional disability.	−	?	
Bredow et al. [111]	Non-specific chronic low back pain (NSCLBP): Which conservative therapy shows an evident effectiveness—A review of the current literature	Back pain—chronic non-specific (low)	Chronic	MR	1 SR	N	In a Cochrane analysis by Khadilkar et al. [100] regarding the use of TENS there is no evidence regarding the treatment of Non-specific chronic low back pain	?	?	Article in German No RCTS were included
Jauregui et al. [52]	A Meta-Analysis of Transcutaneous Electrical Nerve Stimulation for Chronic Low Back Pain	Back—chronic low	Chronic	SR	12	Y	Treatment of chronic low back pain with TENS demonstrated significant pain reduction. The application of TENS may lead to less pain medication usage and should be incorporated into the treatment armamentarium for chronic low back pain	+	?	
Ehrenbrusthoff et al. [112]	Physical therapy management of older adults with chronic low back pain: A systematic review	Back—chronic low	Chronic	SR	0	N	No statement of conclusion for TENS	?	?	Book chapter
van Middelkoop et al. [53]	A systematic review on the effectiveness of physical and rehabilitation interventions for chronic non-specific low back pain	Back pain—chronic low	Chronic	SR	6	Y	The data provided low quality evidence (serious limitations, heterogeneity) that there is no statistically significant difference on post-treatment pain intensity and disability between TENS and sham-TENS	−	?	
Chou [113]	Low back pain (chronic)	Back pain—chronic low	Chronic	SR	3	N	Compared with placebo: We don’t know whether TENS is more effective at reducing pain in people with chronic low back pain (very low-quality evidence). Compared with sham TENS plus massage: TENS plus massage may be no more effective at reducing pain in people with chronic low back pain (low-quality evidence).	?	?	Update of Hall and McIntosh [114]
Dubinsky et al. [115]	Assessment: Efficacy of transcutaneous electric nerve stimulation in the treatment of pain in neurologic disorders (an evidence-based review)	Back—chronic low	Chronic	SR	4	N	TENS is not recommended for the treatment of chronic low back pain (Level A)	−	?	Conducted two analyses in same report—this is data for back pain only. See rebuttal by Johnson and Walsh [116]
Gaid et al. [117]	The role of transcutaneous electric nerve stimulation (TENS) for the management of chronic low back pain	Back—chronic low	Chronic	SR	3	N	…evidence supporting the use TENS as a short-term effective treatment modality for chronic low back pain. Evidence of a longer-term effect is equivocal.	+	?	
Gutiérrez et al. [118]	Evidence of the analgesic effect of physiotherapy in the low backpain syndrome	Low Back Pain	Both	SR	4	N	…controversial evidence regarding the use of laser and TENS in sub-acute and chronic low back pain	?	?	
Machado et al. [86]	Analgesic effects of treatments for non-specific low back pain: a meta-analysis of placebo-controlled randomized trials	Back pain—chronic low	Both	SR	4	Y	No statement of conclusion for TENS	?	?	No numerical data of effect size but the forest plot revealed the upper confidence interval was in favour of TENS and did not bisect the line of no difference
Khadilkar et al. [100]	Transcutaneous electrical nerve stimulation (TENS) versus placebo for chronic low-back pain (Review)	Back—chronic low	Chronic	CR	5	N	…evidence from the small number of placebo-controlled trials does not support the use of TENS in the routine management of chronic low back pain	−	?	Updates of Khadilkar et al. [119] and Milne et al. [120]
Poitras et al. [87]	Evidence-informed management of chronic low back pain with transcutaneous electrical nerve stimulation, interferential current, electrical muscle stimulation, ultrasound, and thermotherapy	Back pain—chronic low	Chronic	SR	4	Y	Globally, high and low-frequency TENS appears to have an immediate impact on pain intensity, with results favoring high-frequency TENS	+	?	The North American Spine Society sponsored this special focus issue.
Chou [61]	Nonpharmacologic Therapies for Acute and Chronic Low Back Pain: A Review of the Evidence for an American Pain Society/American College of Physicians Clinical Practice Guideline	Back pain—acute and chronic	Both	MR	4 6 SRs	N	Other non-invasive therapies (back schools, interferential therapy, low-level laser therapy, lumbar supports, TENS, traction, and ultrasonography) have not been shown to be effective for either chronic or subacute or acute low back pain	−	?	
Keller et al. [63]	Effect sizes of non-surgical treatments of non-specific low-back pain	Back pain—chronic low	Chronic	SR	2	Y	TENS and manipulation had small effect sizes	?	?	
Brosseau et al. [62]	Efficacy of the transcutaneous electrical nerve stimulation for the treatment of chronic low back pain—A meta-analysis.	Back pain—chronic low	Chronic	SR	5	Y	The results of the meta-analysis present no evidence to support the use or non-use of TENS alone in the treatment of chronic low back pain	?	?	
Pengel et al. [121]	Systematic review of conservative interventions for subacute low back pain	Back pain—subacute low	Both	SR	3	N	…there is evidence that…other treatments (e.g., manipulation, exercise, TENS) may be effective	+	?	
Philadelphia Panel [64]	Philadelphia Panel Evidence-Based Clinical Practice Guidelines on Selected Rehabilitation Interventions for Low Back Pain	Back pain—low, non-specific	Both	SR	5	Y	…a lack of evidence regarding efficacy of TENS for Acute LBP (<4 Weeks), Level I (RCT), Grade C for Pain or Function (No Benefit Demonstrated)	?	?	Detailed analysis
Flowerdew and Gadsby [122]	A review of the treatment of chronic low back pain with acupuncture-like transcutaneous electrical nerve stimulation and transcutaneous electrical nerve stimulation	Back pain–chronic low	Chronic	SR	6	Y	There is limited statistical evidence that ALTENS and TENS reduce pain and improve function in patients with chronic low back pain, at least in the short term	+	?	
Gadsby et al. [123]	Low back pain	Back pain—low	Chronic	SR	6	Y	…clear evidence that conventional TENS and acupuncture-like TENS reduce pain and increase range of motion of patients with chronic low back pain	+	?	
**Low Back Pain (Acute) 2 reviews**										
Binny et al. [85]	Transcutaneous electric nerve stimulation (TENS) for acute low back pain: systematic review	Back pain, low, acute	Acute	SR	3	Y	…is insufficient [evidence] to support or dismiss the use of TENS for acute low back pain	?	?	
McIntosh and Hall [124]	Low back pain (acute)	Back pain—low	Acute	SR	0	N	No statement of conclusion for TENS	?	?	
**Non-specific neck pain 8 reviews**										
Martimbianco et al. [125]	Transcutaneous electrical nerve stimulation (TENS) for chronic neck pain	Neck pain—chronic	Chronic	SR	7	N	…there was very low-certainty evidence from two trials about the effects of conventional TENS when compared to sham TENS at short-term	?	?	This is described as a ‘split’ from Kroeling 2013’—we include because different team and over 5 years elapsed since original
(Kroeling et al., 2013)	Electrotherapy for neck pain (Review)	Neck Pain	UC	CR	10	N	TENS…might be more effective than placebo (very low quality evidence)	+	?	
Binder et al. [126]	Clinical Evidence Neck pain	Neck pain—various…	Both	SR	1	N	We don’t know whether…TENS,…are better or worse than other treatments at reducing [various types of acute and chronic neck pain]	?	?	
Jensen et al. [56]	Neck pain	Neck pain—non-specific	UC	OSR	0	N	symptom relief this condition can be treated with TENS	+	?	
Vernon et al. [127]	A systematic review of conservative treatment for acute neck pain not due to whiplash	Neck pain—not whiplash	Acute	SR	1	N	One trial 47 provides some evidence that TENS treatment is beneficial over a 3-week interval	?	?	
Philadelphia Panel [128]	Philadelphia Panel Evidence-Based Clinical Practice Guidelines on Selected Rehabilitation Interventions for Neck Pain	Neck pain—non-specific	Both	SR	1	N	…a lack of evidence regarding efficacy…TENS for acute neck pain	?	?	
Kjellman et al. [129]	A critical analysis of randomised clinical trials on neck pain and treatment efficacy. A review of the literature	Neck pain	Both	SR	3	N	[No statement of conclusion for TENS]	U	?	No conclusion on TENS
Aker et al. [130]	Conservative management of mechanical neck pain: systematic overview and meta-analysis	Mechanical neck pain	Both	SR	2	No	…no treatments [including TENS] have been studied in enough detail to assess either efficacy or effectiveness	?	?	Article is a summary of Gross et al. [131]

Key: OSR = overview of systematic reviews; SR = systematic review; CR = Cochrane review; MR = mixed review; Y = yes; *n* = no; The column ‘Authors’ judgement’: + = evidence tending to favour TENS, − = evidence tending not to favour TENS, ? = evidence tending to be conflicting, inconclusive or insufficient to make a judgement; The column ‘Our Judgement’: + = Sufficient evidence to judge—TENS beneficial; − = Sufficient evidence to judge—TENS not beneficial; +/− = Sufficient evidence to judge—inconclusive; ? = Insufficient evidence to judge.

**Table 24:** Synopsis of the analyses of characteristics and outcomes of included reviews.

Condition	Quantity of Included Reviews	Comment—Quality of Reviews	Comment—Quantity and Quality of RCT Data	Judgement—Analgesic Efficacy
Mixtures of painful conditions	15	One OCR, CRs and some SRs of high methodological quality Strongest review: Chronic pain—Gibson et al. [6]; Acute pain—Johnson et al. [83]	Well over 200 different studies on TENS for any type of pain cited in the included reviews. Majority of studies have small (inadequate) sized samples Strongest RCT: Dailey et al. [224]	+
Musculoskeletal pain including non-specific low back or neck pain	40 reviews including 23 reviews on non-specific back pain	CRs and some SRs are high methodological quality Strongest review: Johnson and Martinsson [10] with Wu et al. [14] and Jauregui et al. [52] also notable	Some moderately sized studies well designed studies but most RCTs have small (inadequate) sample sizes. More reviews than studies for back and for neck pain Majority of RCTs have small samples Strongest RCTs: Warke et al. [37], Deyo et al. [49]	++
Osteoarthritis	11 reviews	CRs and some SRs are high methodological quality Strongest review: Chen et al. [77] and Rutjes et al. [78]	Some moderately sized studies well designed studies but most RCTs have small (inadequate) sample sizes Strongest RCT: Atamaz et al. [225] Palmer et al. [226]	+
Post-operative pain	13 reviews	Some SRs are high methodological quality Strongest review: Zhou et al. [88] and Bjordal et al. [11]	Some moderately sized studies well designed studies but most RCTs have small (inadequate) sample sizes Strongest RCT: Rakel et al. [227]	++
Labour pain	10 reviews	CRs and some SRs are high methodological quality Strongest reviews: Thuvarakan et al. [26] and Dowswell et al. [22]	Some moderately sized studies well designed studies but most RCTs have small (inadequate) sample sizes Strongest RCT: Baez-Suarez et al. [228], Tsen et al. [229], Thomas et al.,1988 [230]	+
Dysmenorrhea and Pelvic pain	12 reviews	Some SRs are high methodological quality Strongest review: Cottrell et al. [71] and Arik et al. [70]	Few moderately sized studies well designed studies but most RCTs have small (inadequate) sample sizes Strongest RCT: Bai et al. [231]	+
Fibromyalgia	6 reviews	CRs and some SRs are high methodological quality Strongest review: Johnson et al. [156] and Megia Garcia et al. [155]	One very high quality moderately sized study Strongest RCT: Dailey et al. [224]	+
Cancer and its treatment	5 reviews	CRs and some SRs are high methodological quality Strongest review: Hurlow et al. [168]	No suitable RCTs	?
Specific shoulder conditions	5 reviews	One CR of high methodological quality Strongest reviews: Page et al.—rotator cuff disease [176]	Few studies and most RCTs have small (inadequate) sample Strongest RCT: Baskurt et al. [232]	+
Peripheral diabetic neuropathy	5 reviews	Some SRs are high methodological quality Strongest review: Zeng et al. [169] and Stein et al. [65]	Some moderately sized studies well designed studies but most RCTs have small (inadequate) sample sizes Strongest RCT: Bulut et al. [233]	+
Other painful conditions	47 reviews	CRs and some SRs are high methodological quality Strongest reviews: Gibson et al.—neuropathic pain [13], Fernandez-Tenorio et al.—spasticity [201]	Some moderately sized studies well designed studies but most RCTs have small (inadequate) sample sizes Strongest RCT: Amer-Cuenca et al. [234]—procedural pain	?
Overall	169 reviews	CRs and some SRs are high methodological quality	Strongest RCT: Dailey et al. [224]	+

Key: OSR overview of systematic reviews, CR Cochrane review, SR systematic review, RCT randomised controlled trial. Judgement for benefit: likelihood of clinically meaningful relief of pain: ++ = Reasonable evidence of benefit, + = Inconclusive but tending toward benefit, ? = Inconclusive, − = Inconclusive but tending toward no benefit, −− Reasonable evidence of no benefit.

## Data Availability

Extracted data is available on request from M.I.J.

## Supplementary Materials

The following are available online at https://www.mdpi.com/article/10.3390/medicina57101060/s1, and includes Search Terms: Operational Aide Memoires and a Table of Excluded Records with Reasons [245,246,247,248,249,250,251,252,253,254,255,256,257,258,259,260,261,262,263,264,265,266,267,268,269,270,271,272,273,274,275,276,277,278,279,280,281,282,283,284,285,286,287,288,289,290,291,292,293,294,295,296,297,298,299,300,301,302,303,304,305,306,307,308,309,310,311,312,313,314,315,316,317,318,319,320,321,322,323,324,325,326,327,328,329,330,331,332,333,334,335,336,337,338,339,340,341,342,343,344,345,346,347,348,349,350,351,352,353,354,355,356,357,358].
