# Supplementary material for: Does TENS Reduce the Intensity of Acute and Chronic Pain? A Comprehensive Appraisal of the Characteristics and Outcomes of 169 Reviews and 49 Meta-Analyses
Source: Medicina (Kaunas). 2021 Oct 4;57(10):1060. doi: 10.3390/medicina57101060 (PMC8539683; doi:10.3390/medicina57101060)
Supplement: Supplementary file 1 [file medicina-57-01060-s001.zip › medicina-1347923-supplementary.pdf]

Supplementary Material

Contents

1. Search Terms: Systematic Reviews 2
2. Operational Aide Memoires..... 2
2.1. Eligibility Criteria for Inclusion of Systematic Reviews—Aide Memoire for Eligibility Screening 2
2.1.1. Gross Screening of Report Titles and Abstract 2
2.1.2. Fine Grain Screening of Full Reports..... 2
2.2. Assessing Quality..... 2
2.3. Categorising Efficacy 3
3. Table S1 - Excluded Records with Reasons 3

1. Search Terms: Systematic Reviews
MEDLINE Search Strategy for systematic reviews:
1. EXP Transcutaneous Electric Nerve Stimulation/
2 TENS.ti,ab
3 TNS.ti,ab
4 ENS.ti,ab
5 transcutaneous electric* nerve stimulation.ti,ab.
6 transcutaneous nerve stimulation.ti,ab
7 electric* nerve stimulation.ti,ab
8 electrostimulation therap*.ti,ab
9 electro-stimulation therap*.ti,ab.
10 electric* nerve therap*.ti,ab
11 electroanalgesi*.ti,ab
12 transcutaneous electric* stimulation.ti,ab.
13 TES.ti,ab
14 or/1-13
15 Pain
16 Systematic review. Pt.
17 Meta-analysis.pt.
18 16 OR 17
19 14 AND 15 AND 18

2. Operational Aide Memoires

2.1. Eligibility Criteria for Inclusion of Systematic Reviews—Aide Memoire for Eligibility Screening

2.1.1. Gross Screening of Report Titles and *A*bstract
Do not carry forward if title/abstract indicates …
1. Definitely NOT acute or chronic pain
2. Definitely NOT adult participants aged 18 years or above
3. Definitely NOT on TENS
    - carry forward if on electrotherapy and then extract RCTs on TENS
    - carry forward if uncertain whether SR focussed on 'standard TENS' (e.g. TENS characteristics (type of currents), type and location of electrodes (acupoints, single probe electrode etc.) and/or type of device (i.e. TENS-like)

Obtain full report
2.1.2. Fine Grain Screening of Full Reports
Exclude if full report indicates …
1. Definitely NOT acute or chronic pain
2. Definitely NOT adult participants aged 18 years or above
3. Definitely NOT on TENS
    - carry forward if on electrotherapy and extract RCTs on TENS – include reports with TENS in scope but fail to identify any TENS SRs
    - carry forward if uncertain whether SR focussed on 'standard TENS' (e.g. TENS characteristics (type of currents), type and location of electrodes (acupoints, single probe electrode etc.) and/or type of device (i.e. TENS-like)

Lock included SRs

2.2. Assessing Quality

Quality of evidence stated by review authors

2.3. Categorising Efficacy

We categorised as efficacy as:

- sufficient evidence in favour of TENS (+)
    - pooled analysis of ≥500 events or at least one RCT with ≥200 participants in each arm of the trial
- sufficient evidence in favour of control/placebo (-)
    - pooled analysis of ≥500 events or at least one RCT with ≥200 participants in each arm of the trial
- sufficient evidence that is conflicting/inconclusive (=)
    - no analysis of pooled data and at least two RCTs with ≥200 participants in each arm of the trial that are conflicting
- insufficient evidence to make a judgement (?)
    - pooled analysis of <500 events or no RCTs with ≥200 participants in each arm of the trial

3. Table S1 - Excluded Records with Reasons

Key; Review Type: CR = Cochrane review, SR = Non-Cochrane systematic review (systematic search), DR = Non-Cochrane descriptive review; OSR = Non-Cochrane overview of systematic reviews, OCR = Overview of Cochrane reviews, MA; Meta-analysis of a pain outcome with an effect size estimate provided

Coding for exclusion

- TENS not in scope
- TENS was not evaluated as a primary comparator i.e. TENS was a possible comparator rather than the primary treatment
- TENS in scope but not evaluating pain intensity
- Not evaluating 'standard TENS'
- Not a review
- A review but not using a systematic search for RCTs
- Duplicate of analysis of a review already included
- Analysis subsequently updated – update included in our review
- Not evaluating clinical pain – healthy human participants

Table S1. Excluded Records with Reasons

Authors	Ref.	Title	*Reason for Exclusion*
Almeida et al.	[245]	Effects of Transcutaneous Electrical Nerve Stimulation on Proinflammatory Cytokines: Systematic Review and Meta-Analysis.	TENS in scope but not evaluating pain intensity
Amatya et al.	[199]	Nonpharmacological interventions for spasticity in multiple sclerosis.	Analysis subsequently updated – update included in our review by Amatya et al. [192]
Amer-Cuenca et al.	[234]	Pain relief by applying TENS during un-sedated colonoscopy: a randomized double-blind placebo-controlled trial	Not a review
Anim-Somuah et al.	[246]	Epidural versus non-epidural or no analgesia for pain management in labour	TENS not in scope
Arendt et al.	[247]	Nonpharmacologic labor analgesia	A review but not using a systematic search for RCTs
Barkatsa et al.	[248]	Physiotherapy interventions for the management of whiplash injuries.	A review but not using a systematic search for RCTs
Bedwell et al.	[23]	The use of transcutaneous electrical nerve stimulation (TENS) for pain relief in labour: a review of the evidence	Duplicate of analysis of a review already included – this review discusses the CR by Dowswell et al. [22]
Bennett et al.	[240]	Methodological quality in randomised controlled trials of transcutaneous electric nerve stimulation for pain: low fidelity may explain negative findings	TENS in scope but not evaluating pain intensity
Binder et al.	[249]	Comment on Dubinsky et al. [115]	Not a review
Bjordal et al.	[250]	Evidence based use of electrophysical agents for managing musculoskeletal pain	Not a review
Briones-Areán et al.	[251]	Effectiveness of physiotherapy in shoulder impingement syndrome.	TENS not in scope
Brosseau et al.	[252]	Efficacy of transcutaneous electrical nerve stimulation (TENS) for rheumatoid arthritis: a systematic review.	Not a review - Commentary on Cochrane review Brosseau et al. [205]
Cadalso et al.	[253]	Efficacy of Electrical Stimulation of the Occipital Nerve in Intractable Primary Headache Disorders: A Systematic Review with Meta-Analyses.	TENS not in scope – Occipital nerve stimulation
Cameron et al.	[254]	Transcutaneous Electrical Nerve Stimulation (TENS) for dementia	TENS in scope but not evaluating pain
Campo-Prieto et al.	[255]	Effectiveness of mirror therapy in phantom limb pain: A literature review	TENS not in-scope
Carroll et al.	[152]	Transcutaneous electrical nerve stimulation in labour pain: a systematic review (in *BJOG*)	Analysis subsequently updated – update included in our review by Carroll et al. [144]
Chan et al.	[256]	Postherpetic neuralgia: Review of treatment modalities	Not using a systematic search for RCTs
Chaparro et al.	[257]	Opioids compared to placebo or other treatments for chronic low-back pain	TENS not in scope
Chen et al.	[258]	Does the pulse frequency of transcutaneous electrical nerve stimulation (TENS) influence hypoalgesia? A systematic review of studies using experimental pain and healthy human participants	Not evaluating clinical pain – healthy human participants
Chesterton et al.	[259]	Transcutaneous electrical nerve stimulation for the management of tennis elbow: a pragmatic randomized controlled trial: the TATE trial	Not a review
Choi et al.	[260]	Acupuncture and related interventions for the treatment of symptoms associated with carpal tunnel syndrome.	TENS not in scope
Claydon et al.	[261]	Dose-specific effects of transcutaneous electrical nerve stimulation (TENS) on experimental pain: a systematic review	Not evaluating clinical pain – healthy human participants
Claydon et al.	[262]	The hypoalgesic efficacy of TENS parameters on experimental pain models in healthy humans: a systematic review	Not evaluating clinical pain – healthy human participants

Cruccu et al.	[21]	EFNS guidelines on neurostimulation therapy for neuropathic pain	TENS not in scope –TENS was in scope in previous version and we have included this earlier version in our review Cruccu et al. [20]
Deussen et al.	[216]	Analgesia for relief of pain due to uterine cramping/involution after birth (Review)	Analysis subsequently updated – update included in our review by Deussen et al. [215]
Dingemanse et al.	[263]	Epicondylitis	This was the e-pub version citation of the paper that was excluded as a duplicate at full text screening stage – we included the final review by Dingemanse et al. [185]
Doyle et al.	[264]	Interventions for sensory impairment in the upper limb after stroke.	TENS in scope but not evaluating pain intensity
Ducic et al.	[265]	A systematic review of peripheral nerve interventional treatments for chronic headaches.	TENS not in scope
Eberhart et al.	[266]	Transient neurologic symptoms after spinal anesthesia. A quantitative systematic overview (meta-analysis) of randomized controlled studies].	TENS not in scope
Eccleston et al.	[267]	Interventions for the reduction of prescribed opioid use in chronic non-cancer pain.	TENS in scope but not evaluating pain intensity – the review only included studies where there was prescribed opioid use - pain intensity was a secondary outcome
Ely et al.	[268]	Transcutaneous electrical nerve stimulation (TENS) at acupuncture points for the management of chronic pain: a narrative review	Not using a systematic search for RCTs - Abstract only
Fargas-Babjak et al.	[269]	Acupuncture, transcutaneous electrical nerve stimulation, and laser therapy in chronic pain	Not a review
Foletti et al.	[270]	Neurostimulation technology for the treatment of chronic pain: A focus on spinal cord stimulation	TENS not in scope
Freynet et al.	[271]	Evidence-based physiotherapy in thoracic surgery after pulmonary resection by thoracotomy	Duplicate of analysis of a review already included - French version of the report by Freynet et al. [142]
Fu et al. et al.	[272]	A mixed treatment comparison on efficacy and safety of treatments for spasticity caused by multiple sclerosis: a systematic review and network meta-analysis	TENS in scope but not evaluating pain intensity - spasticity not pain
Gabler et al.	[273]	Comparison of Transcutaneous Electrical Nerve Stimulation and Cryotherapy for Increasing Quadriceps Activation in Patients with Knee Pathologies	TENS in scope but not evaluating pain intensity
Gadsby et al.	[274]	CLBP	Not a review – Statement of review withdrawn from Cochrane
Gadsby et al.	[275]	CLBP	Not a review – Statement of review withdrawn from Cochrane
Gross et al.	[276]	Manipulation and mobilisation for neck pain contrasted against an inactive control or another active treatment (Review)	TENS was not evaluated as a primary comparator i.e. TENS was a possible comparator rather than the primary treatment
Hall et al.	[277]	Low back pain (acute)	Analysis subsequently updated by McIntosh and Hall [124] – update included in our review
Hall et al.	[114]	Low back pain (chronic)	Analysis subsequently updated by Chou [113] – update included in our review
Handy et al.	[278]	Meta-analysis examining the effectiveness of electrical stimulation in improving functional use of the upper limb in stroke patients	TENS in scope but not evaluating pain intensity
Hawker et al.	[279]	Osteoarthritis year 2010 in review: non-pharmacologic therapy	TENS not in scope
Ho et al.	[280]	Sphenopalatine ganglion: block, radiofrequency ablation and neurostimulation - a systematic review	TENS not in scope
Hoffman et al.	[281]	Commentary - Review: transcutaneous electrical nerve stimulation is not effective for chronic low-back pain	Not a review - Commentary on review by Milne et al. [120]
Hu et al.	[282]	The effectiveness of acupuncture or TENS for phantom limb syndrome. II: A narrative review of case studies	Not a review
Hunsinger et al.	[283]	Adverse event reporting in nonpharmacologic, noninterventional pain clinical trials: ACTTION systematic review	TENS in scope but not evaluating pain intensity

Jamtvedt et al.	[284]	Choice of treatment modalities was not influenced by pain, severity or co-morbidity in patients with knee osteoarthritis	TENS in scope but not evaluating pain intensity
Jayasekara et al.	[285]	Acute pain	Not a review - Commentary on Cochrane review by Walsh et al. that was subsequently updated by Johnson et al. [83]
Johnson et al.	[286]	Transcutaneous Electrical Nerve Stimulation (TENS): a review	Not a systematic search for RCTs
Johnson et al.	[287]	The clinical effectiveness of TENS in pain management	Not a systematic search for RCTs
Johnson et al.	[288]	Transcutaneous electrical nerve stimulation (TENS) and TENS-like devices: do they provide pain relief?	Not a systematic search for RCTs
Johnson et al.	[289]	Transcutaneous electrical nerve stimulation (TENS) as an adjunct for pain management in perioperative settings: A critical review	Not a systematic search for RCTs
Johnson et al.	[290]	Transcutaneous electrical nerve stimulation for the management of painful conditions: Focus on neuropathic pain.	Not a systematic search for RCTs
Jordan et al.	[291]	Interventions to improve adherence to exercise for chronic musculoskeletal pain in adults	TENS not in scope
Kang et al.	[292]	Therapeutic methods for knee osteoarthritis: randomized controlled trial and systemic evaluation	TENS not in scope
Khadilkar et al.	[119]	Transcutaneous electrical nerve stimulation (TENS) for chronic low-back pain (Review)	Analysis subsequently updated – update included in our review by Khadilkar et al. [100]
Khadilkar et al.	[293]	Transcutaneous electrical nerve stimulation for the treatment of chronic low back pain: a systematic review	Analysis subsequently updated - This is a journal version (Spine 2005; 30(23):2657-66) of the Cochrane review published by Khadilkar et al. [119]
Khalil et al.	[294]	Treatment for meralgia paraesthetica	TENS not in scope – conservative measures were in scope but defined as 'Conservative advice may include avoidance of external compressive and traumatic factors' rather than electrophysical agents.
Khan et al.	[295]	Non-pharmacological interventions for spasticity in adults: An overview of systematic reviews	TENS in scope but not evaluating pain intensity - spasticity not pain
Kirpalani et al.	[296]	Comparison of 2 methods of non-invasive treatment between transcutaneous electrical stimulation and pulsed electromagnetic field stimulation as replacement of invasive manual acupuncture	Not a review
Klomp et al.	[297]	Inhaled analgesia for pain management in labour.	TENS was not evaluated as a primary comparator i.e. TENS was a possible comparator rather than the primary treatment
Koes et al.	[298]	Spinal manipulation and mobilisation for back and neck pain	TENS was not evaluated as a primary comparator i.e. TENS was a possible comparator rather than the primary treatment
Koes et al.	[299]	Spinal manipulation for low back pain. An updated systematic review of randomized clinical trials	TENS was not evaluated as a primary comparator i.e. TENS was a possible comparator rather than the primary treatment
Knotkova et al.	[12]	Neuromodulation for chronic pain	Not using a systematic search for RCTs
Koopman et al.	[300]	Treatment for postpolio syndrome.	TENS not in scope
Kosseim et al.	[301]	Implementing evidence-based physiotherapy practice for treating children with low back pain: are we there yet?	Not using a systematic search for RCTs
Kroeling et al.	[302]	Electrotherapy for neck disorders (Review)	Analysis subsequently updated – update included in our review by Kroeling et al. [132]
Kus and Yeldan	[303]	Strengthening the quadriceps femoris muscle versus other knee training programs for the treatment of knee osteoarthritis	TENS was not evaluated as a primary comparator i.e. TENS was a possible comparator rather than the primary treatment
Kwan et al.	[196]	Pain relief for women undergoing oocyte retrieval for assisted reproduction (Review)	TENS was a Cochrane review - analysis subsequently updated and included in our review Kwan et al.[189]
Lee et al.	[304]	Some Non-FDA Approved Uses for Neuromodulation: A Review of the Evidence.	TENS not in scope

Lenza et al.	[305]	Surgical versus conservative interventions for treating fractures of the middle third of the clavicle.	TENS was not evaluated as a primary comparator i.e. TENS was a possible comparator rather than the primary treatment - focus of review was surgical interventions compared with conservative treatments - no TENS RCTs were found
Lin et al.	[306]	Rehabilitation for ankle fractures in adults.	TENS in scope but not evaluating pain intensity
Lindsley et al.	[307]	Non-surgical interventions for acute internal hordeolum	TENS not in scope
Mahmood et al.	[308]	Effect of Transcutaneous Electrical Nerve Stimulation on Spasticity in Adults with Stroke: A Systematic Review and Meta-analysis	TENS in scope but not evaluating pain intensity - spasticity not pain
Marcolino et al.	[309]	Effects of transcutaneous electrical nerve stimulation alone or as additional therapy on chronic post-stroke spasticity: systematic review and meta-analysis of randomized	TENS in scope but not evaluating pain intensity - spasticity not pain
Martí-Carvajal et al.	[310]	Interventions for treating painful sickle cell crisis during pregnancy	TENS not in scope
McQuay et al.	[3]	Systematic review of outpatient services for chronic pain control. Chapter 8 Transcutaneous electrical nerve stimulation	Duplicate of analysis of a review already included by Carroll et al.[144] which was an update of Carrol et al. [152] and reports on acute pain from McQuay et al. [4]
Milne et al.	[120]	Transcutaneous electrical nerve stimulation (TENS) for chronic low back pain	Analysis subsequently updated – update included in our review by Khadilkar et al. [100]
Miller et al.	[311]	Manual therapy and exercise for neck pain: Clinical treatment pocket notes.	Not a systematic search for RCTs
Moisset et al.	[312]	Neuromodulation techniques for acute and preventive migraine treatment: a systematic review and meta-analysis of randomized controlled trials	Not evaluating 'standard TENS' - supraorbital TENS
Mollon et al.	[313]	Electrical stimulation for long-bone fracture-healing: a meta-analysis of randomized controlled trials	TENS in scope but not evaluating pain intensity
Monaghan et al.	[314]	Surface neuromuscular electrical stimulation for quadriceps strengthening pre and post total knee replacement	TENS not in scope
Muller et al.	[315]	Apropos the meta-analyses, randomization and postoperative pain relief with transcutaneous nerve stimulation.	Not a review - Commentary on review Carroll et al. [5]
Mulvey et al.	[316]	Phantom pain and stump pain following amputation in adults	Analysis subsequently updated – update included in our review by Johnson et al. [203]
Mulvey et al.	[317]	Transcutaneous Electrical Nerve Stimulation for Phantom Pain and Stump Pain in Adult Amputees	Not a review – open label study
Mulvey et al.	[318]	Transcutaneous electrical nerve stimulation (TENS) for phantom pain and stump pain following amputation in adults: an extended analysis of excluded studies from a Cochrane systematic review	Analysis subsequently updated – this is an extended report of Mulvey et al. [316] which was subsequently updated and included in our review by Johnson et al. [203]
Munirama et al.	[319]	A systematic review and meta-analysis of ultrasound versus electrical stimulation for peripheral nerve location and blockade	TENS in scope but not evaluating pain intensity
Nnoaham et al.	[236]	Withdrawn: TENS for chronic pain	Not a review – Statement that review withdrawn from Cochrane
Novak et al.	[320]	How clinically relevant is a meta-analysis of electrical nerve stimulation when based on heterogeneous disease states?	Not a review - Commentary on review Johnson and Martinson [10]
O'Connell et al.	[321]	Non-invasive brain stimulation techniques for chronic pain	TENS not in scope – analysis subsequently updated
O'Connell et al.	[322]	Non-invasive brain stimulation techniques for chronic pain.	TENS not in scope – updated review of O'Connell et al. [321]
O'Gallagher et al.	[323]	Systemic treatment for blepharokerato conjunctivitis in children	TENS not in scope
Pelland et al.	[324]	Electrical stimulation for the treatment of rheumatoid arthritis (Review)	Analysis subsequently updated – update included in our review by Brosseau et al. [205]
Peters et al.	[213]	Rehabilitation following carpal tunnel release	TENS not in scope
Poropat et al.	[325]	Enteral nutrition formulations for acute pancreatitis.	TENS not in scope nor evaluating pain intensity

Price et al.	[326]	Electrical stimulation for preventing and treating post-stroke shoulder pain: a systematic Cochrane review	Duplicate of analysis of a review already included by Price and Pandyan [72] – this is a journal report in *Clinical Rehabilitation* of Cochrane review with identical analysis but one less RCT
Proietti Cecchini et al.	[327]	Emerging therapies for chronic migraine.	TENS not in scope
Redgrave et al.	[328]	Safety and tolerability of Transcutaneous Vagus Nerve stimulation in humans	TENS not in scope – using vagal TENS rather than standard TENS
Reed et al.	[329]	Review of acute and chronic pain published studies	Not using a systematic search for RCTs
Renner et al.	[330]	Pain control in first trimester surgical abortion.	TENS not in scope
Richards et al.	[331]	Neuromodulators for pain management in rheumatoid arthritis	TENS not in scope
Riker	[266]	Efficacy of TENS in the treatment of pain in neurologic disorders (an evidence-based review)	Not a review – Comment on Dubinsky et al. [115]
Robb et al.	[180]	Transcutaneous electric nerve stimulation (TENS) for cancer pain in adults (Review)	Analysis subsequently updated – update included in our review by Hurlow et al. [168]
Robb et al.	[332]	A Cochrane Systematic Review of Transcutaneous Electrical Nerve Stimulation for Cancer Pain	This is a duplicate of analysis of a review already included by Robb et al. [180] published in *Journal of Pain & Symptom Management*
Rojahn et al.	[333]	Transcutaneous Electrostimulation for Osteoarthritis of the Knee.	Not a review - Commentary on Rutjes et al. [78]
Rome et al.	[334]	Interventions for preventing and treating stress fractures and stress reactions of bone of the lower limbs in young adults	TENS in scope but not evaluating pain intensity
Rosted et al.	[335]	Use of stimulation techniques in pain treatment	TENS not in scope
Saarto et al.	[336]	Antidepressants for neuropathic pain – Cochrane review	TENS not in scope
Saulle et al.	[337]	Supervised dosing with a long-acting opioid medication in the management of opioid dependence	TENS not in scope
Scott et al.	[338]	Managing low back pain in the primary care setting: The know-do gap	Not using a systematic search for RCTs
Singh et al.	[339]	Complementary and Alternative Medicine in Cancer Pain Management: A Systematic Review	TENS not in scope
Sluka et al.	[340]	What Makes Transcutaneous Electrical Nerve Stimulation Work? Making Sense of the Mixed Results in the Clinical Literature.	Not a review – Commentary
Smith et al.	[341]	Massage, reflexology and other manual methods for pain management in labour	TENS not in scope
Stuiver et al.	[342]	Conservative interventions for preventing clinically detectable upper limb lymphoedema in patients who are at risk of developing lymphoedema after breast cancer therapy	TENS in scope but not evaluating pain intensity
Tashani and Johnson	[343]	TENS. A Possible Aid for Pain Relief in Developing Countries?	Not using a systematic search for RCTs – narrative review
Tirlapur et al.	[344]	Nerve stimulation for chronic pelvic pain and bladder pain syndrome: A systematic review	TENS not in scope
Twycross et al.	[345]	Paediatric nurses' postoperative pain management practices in hospital based non-critical care settings: A narrative review	Not using a systematic search for RCTs
Tzortziou Brown et al.	[346]	Professional interventions for general practitioners on the management of musculoskeletal conditions.	TENS not in scope
Veves et al.	[347]	Painful diabetic neuropathy: epidemiology, natural history, early diagnosis, and treatment options.	TENS not in scope
Walsh et al.	[348]	The evolution of TENS	Not using a systematic search for RCTs
Walsh et al.	[349]	Transcutaneous electrical nerve stimulation for acute pain (2009)	Analysis subsequently updated by Walsh et al. 2015 [355]
Walsh et al.	[350]	Transcutaneous electrical nerve stimulation for acute pain (2015)	Analysis subsequently updated – update included in our review by Johnson et al. [83]
Weiner et al.	[351]	Complementary and alternative approaches to the treatment of persistent musculoskeletal pain.	TENS not in scope
Wiffen	[352]	Pain and palliative care in The Cochrane Library	Not a review
Wiffen	[353]	Pain and palliative care in The Cochrane Library	Not a review
Wiffen	[354]	Pain and palliative care in The Cochrane Library	Not a review

Wiffen	[355]	Pain and palliative care in The Cochrane Library	Not a review
Wiffen	[356]	Carbamazepine for chronic neuropathic pain and fibromyalgia in adults.	TENS not in scope
Zhang et al.	[357]	OARSI recommendations for the management of hip and knee osteoarthritis, Part II: OARSI evidence-based, expert consensus guidelines	Secondary report - Did not include the analysis of efficacy. Analysis of efficacy presented in Part I by Zhang et al. [55]
Zakrzewska et al.	[358]	Neurosurgical interventions for the treatment of classical trigeminal neuralgia	TENS not in scope
